# A systematic review of brain health in adults with chronic pain

**DOI:** 10.1111/anae.70021

**Published:** 2025-10-14

**Authors:** Angeline Lee, Sara Al‐Dahwi, Thomas Angell, Abinaya Arulalagan, Ryan Bloxsom, Harry Clarkson, Rose Faure, Soutiam Goodarzi, Minshu Gupta, Adithya Kale, Kin Lam, Freyia Mahon‐Daly, Chloe Parry, Vidushi Pradhan, Finlay Ryan‐Phillips, Suraj Shah, Serina Sidhu, John Smiddy, Aparna Sridhar, Syed F. Tahmid, Robert Wight, Nia Roberts, Anya Topiwala

**Affiliations:** ^1^ Big Data Institute, Nuffield Department of Population Health University of Oxford Oxford UK; ^2^ Oxford University Medical School, Medical Sciences Division University of Oxford Oxford UK; ^3^ Health Education Thames Valley Oxford UK; ^4^ Health Education England London UK; ^5^ Wexham Park Hospital, Frimley Health NHS Foundation Trust Slough UK; ^6^ Bodleian Health Care Libraries, University of Oxford Oxford UK

**Keywords:** brain age, brain health, chronic pain, dementia, grey matter volumes

## Abstract

**Introduction:**

Recent research has linked chronic pain with an increased risk of clinical dementia diagnosis. Yet structural and functional brain changes associated with chronic pain and their potential role in accelerating brain ageing have not been characterised comprehensively. Understanding these effects is crucial to developing targeted prevention and management strategies.

**Methods:**

We conducted a systematic review of all English language articles in MEDLINE and Embase. Studies were eligible if they compared neuroimaging, clinical, biological, cognitive or mental health outcomes in adults with chronic pain to healthy controls. Following screening, data were extracted and the risk of bias was assessed.

**Results:**

Of 5805 identified studies, 365 met the inclusion criteria. Most were cross‐sectional studies with small sample sizes; conducted in middle‐aged populations in China or the USA; had moderate to high risk of bias; and represented > 30 distinct pain phenotypes. Magnetic resonance imaging was the most common method for assessing brain health. Key findings in patients with chronic pain included: lower grey matter volumes and reduced fractional anisotropy; evidence of accelerated brain ageing including older brain age and higher white matter hyperintensities; mixed results in resting state functional connectivity; increased power densities and connectivity on electroencephalography; and higher levels of serum brain‐derived neurotrophic factor. The most consistently affected brain regions across magnetic resonance imaging studies were the insula; anterior and posterior cingulate; thalamus; hippocampus; primary motor cortex; and cerebellum.

**Discussion:**

Adults with chronic pain exhibit widespread alterations in brain health compared with healthy controls. Several observed features overlap with biomarkers of Alzheimer's disease and other forms of neurodegeneration. These findings highlight the need for larger, well‐designed studies incorporating clearly defined pain phenotypes, multimodal imaging and causal inference methods to clarify the role of chronic pain in brain ageing and dementia risk.

## Plain Language Summary

We reviewed all studies written in English on adults with long‐term (chronic) pain and brain health. We searched two large databases up to 1 November 2024. Studies had to compare people with chronic pain to people without pain. We collected their results and checked each study for quality. Some research has shown that people with chronic pain have a higher chance of a dementia diagnosis in later life. We do not fully know how chronic pain links to brain changes or to faster brain ageing. By bringing many studies together, we aimed to map the main brain changes linked to chronic pain to improve care and find targets for treatment. We found 365 suitable studies to compare. Most were small studies done at a single time point in middle‐aged adults, often from China or the United States, and many had moderate to high risk of being unfair (biased) because of the way the studies were done. Magnetic resonance imaging (MRI) brain scans were the most common way to measure brain health. On MRI scans, people with chronic pain often had patterns that were like the patterns seen in people with Alzheimer's disease: less grey brain tissue, weaker white brain tissue, and signs of older brain age in places such as the hippocampus (the memory centre of the brain). There were also changes in brain chemical levels and brain activity levels in people with chronic pain compared to those without chronic pain. The findings suggest wide brain changes in people with chronic pain. Larger, better‐designed studies are needed to understand these links more clearly.

## Introduction

Chronic pain, defined as pain that persists or recurs for longer than 3 months [1], affects over one in three adults in the UK [2]. Recent evidence indicates that individuals with chronic pain are at increased risk of developing all‐cause dementia [3, 4, 5, 6]. Both pain and dementia rank among the leading global causes of disability [7, 8], and their co‐occurrence poses a significant and growing public health challenge. These two highly debilitating [9, 10] and costly [11] conditions are especially concerning given the longevity of younger patients, where the absence of disease‐modifying treatments may result in devastating consequences for individuals and wider society.

Pain in older people is extremely common, affecting over 62% of those aged > 75 y [12] and between 60% and 80% of those with dementia [13]. However, it represents a clinical challenge as there are limited strategies available to manage pain in this population across the biopsychosocial spectrum, compared with other patient populations [13]. While observational studies have linked chronic pain to dementia risk, the nature of this relationship remains poorly understood. Clinical dementia diagnosis is a late‐stage marker and pathophysiological changes can precede clinical symptoms by many years [14]. Therefore, there is an urgent need for research examining the association between chronic pain and early markers of brain health, before clinical symptoms of cognitive decline appear.

Brain health is defined as the preservation of optimal brain integrity, cognitive and mental function at a given age, in the absence of overt brain diseases [15]. By investigating a range of brain health outcomes, beyond clinical dementia, we can gain a more nuanced understanding of how pain affects the brain and potentially contributes to pathophysiological processes underlying neurodegeneration.

We previously conducted a scoping review to identify outcome measures used to assess brain health [16]. This identified 210 outcome measures used at least twice that were divided into five categories: imaging; biological; clinical; mental health; and cognitive testing. Among these, magnetic resonance imaging (MRI) was the most frequently used method, featuring in 71% of studies (514/727 studies) [16]. Table 1 summarises the outcomes used most commonly to assess brain health and the common patterns seen in Alzheimer's disease, which is the most prevalent (60% of all dementias) and most studied dementia subtype [36].

**Table 1:** Outcomes used commonly to assess brain health in research and changes for these outcomes seen in Alzheimer's disease.

Common outcome measures assessing brain health	Changes seen in Alzheimer's disease
**Imaging**	
**Structural MRI:**	
Grey matter volumes	Grey matter volume loss (mostly temporal regions) [17]
Hippocampus volume	Hippocampus volume loss [17]
White matter hyperintensities	Higher white matter hyperintensities [18]
**Functional MRI:**	
Cerebral blood flow	Decreased cerebral blood flow [19]
Resting state functional connectivity	Mixed increased and decreased resting state functional connectivity in salience and default mode networks [20, 21]
Task based functional connectivity	Increased and decreased activation during tasks [22]
**Diffusion MRI:**	
Mean diffusivity	Higher mean diffusivity [22]
Fractional anisotropy	Lower fractional anisotropy [22]
**Other imaging outcomes:**	
Brain age gap	Higher brain age gap [23]
Brain atrophy and lesion index	Higher brain atrophy and lesion index [24]
PET amyloid	Higher amyloid β levels on FDG‐PET [21]
PET τ	Higher τ protein levels on FDG‐PET [21]
Transcranial Doppler ultrasound	Lower arterial mean flow velocity and increased pulsatility index on transcranial Doppler ultrasound [25]
Transcranial magnetic stimulation	Lower cortical excitability during transcranial magnetic stimulation [26]
fNIRS	Reduced functional connectivity during task performance during fNIRS [21]
**Cognitive testing**	
‐ Trail making test ‐ Mini Mental State Examination ‐ Stroop test ‐ Rey Auditory verbal learning test ‐ Montreal cognitive assessment ‐ Digit span ‐ Digit symbol substitution test ‐ Verbal fluency ‐ Wechsler Adult Intelligence Scale ‐ California Verbal Learning Test	Most of the listed tests are used routinely to diagnose Alzheimer's disease. Poorer performance seen across all the cognitive tests
**Biological**	
BDNF	Lower BDNF levels [27]
ApoE4 genotype	Higher incidence of ApoE4 genotype [28], higher serum/CSF Aβ42, neurofilament light and τ [28, 29]
Neurofilament light in CSF	Lower BDNF levels [27]
**Clinical**	
EEG	Slowing of brain oscillatory activity (higher power in low‐frequency bands and lower power in high‐frequency bands) on EEG [21]
LIBRA	Increased LIBRA [30]
Mindreader	Slowing of brain oscillatory activity (higher power in low‐frequency bands and lower power in high‐frequency bands) on EEG [21]
**Mental health**	
Barratt impulsiveness scale	Increased impulsivity [31]
Geriatric depression scale	Variable sensitivity and specificity of tools in cognitively impaired populations [32, 33, 34, 35]
Patient health questionnaire‐9	
Hospital Anxiety Depression Scale	
Beck depression inventory	

MRI, magnetic resonance imaging; PET, positron emission tomography; FDG, fludeoxyglucose; fNIRS, functional near infrared spectroscopy; BDNF, brain‐derived neurotrophic factor; CSF, cerebrospinal fluid; EEG, electroencephalography; LIBRA, LIfestyle for BRAin health risk score.

This systematic review aimed to evaluate whether adults with chronic pain differ from healthy controls across a broad set of brain health outcomes including imaging; cognitive; biological; clinical; and mental domains. We hypothesised that individuals with chronic pain exhibit altered brain health consistent with patterns observed in early neurodegeneration and dementia.

## Methods

The protocol for this systematic review was published on PROSPERO [37]. MEDLINE and Embase databases were searched using the following criteria: English language articles published between inception and 1 November 2024. Details of the search strategy are in online Supporting Information Appendix S1.

Articles were included if studies compared adults (aged > 18 y) with chronic pain (any location, lasting ≥ 3 months), with a comparator group of healthy controls without chronic pain. Studies needed to consider any of the top 15 imaging, 10 cognitive or five mental health, clinical and biological outcomes (Table 1) derived from a previously published scoping review on brain health [16]. These were chosen to capture all brain health measurement domains in chronic pain. A higher number of outcomes was chosen from imaging and cognitive domains to reflect the higher frequency of these outcomes used in brain health literature. Studies were not included if they considered: acute pain (< 3 months duration); people with congenital insensitivity to pain; studies where pain was being administered to participants including noxious or innocuous stimuli (e.g. thermal, mechanical, chemical, electrical or incisional pain paradigms); and studies using task‐based functional MRI or task‐based electroencephalography (EEG) as the primary outcome. After title and abstract screening, a preliminary review of the first 100 included abstracts found that task‐based studies were too heterogeneous in their methods to be compared, and we decided not to include task‐based functional MRI or EEG studies. Resting state functional MRI studies that measured amplitude of low frequency fluctuations and regional homogeneity were added to the protocol.

Descriptions of the methods used for each selected variable are below and in Table 1. Structural MRI studies provide information on brain volumes including measures of cortical thickness and white matter hyperintensities, which are diffuse high‐signal shadows in white matter associated with a number of neurological diseases [38]. Resting state functional connectivity studies measure the degree of synchrony of blood oxygen level dependent time‐series between different brain regions at rest [39]. Amplitude of low frequency fluctuations and regional homogeneity are measures of regional brain activity that provide information about local neural activity of the brain at rest [39]. Brain age gap refers to the difference between a machine learning‐predicted brain age of an individual, typically derived from neuroimaging data, and their chronological age. Higher values suggest the brain appears older than expected for its age, potentially reflecting accelerated brain ageing or underlying neuropathology [40]. Cerebral blood flow to various brain regions can be derived from resting‐state functional MRI studies [41]. Diffusion tensor imaging is a technique of assessing microstructural white matter integrity, using voxel‐wise values such as fractional anisotropy, which is a scalar quantity that measures the directionality of the diffusion signal at any given voxel [42]. Transcranial magnetic stimulation is a method of estimating motor cortical or corticospinal tract excitability by applying a magnetic coil outside the scalp [43]. Brain‐derived neurotrophic factor (BDNF) is a neurotrophic factor that supports neuronal survival and maturation in the nervous system [44]. Serum τ and Aβ are the main constituents of amyloid plaque [45]. Resting state EEG studies measure electrical activity, specifically oscillatory activity from post‐synaptic potentials occurring in the neocortex at rest using electrodes placed on the scalp. Electroencephalography waves are organised in five main frequency bands: δ (0.5–4 Hz); θ (4–7 Hz); α (8–13 Hz); β (13–30 Hz); and γ (> 30 Hz) [46].

Search results were uploaded into Covidence (Veritas Health Innovation, Melbourne, Australia). Duplicates were removed both by Covidence and manually during the search process. Screening involved two independent reviewers. An independent third reviewer was used to resolve disagreement, with discussion where required. Data extraction was performed using a customised template that was developed based on a preliminary review of the first 50 included studies at full‐text review (online Supporting Information Appendix S2). Study location was determined from the paper or, if this was not mentioned or an international cohort was used, the country of the first author's institution was entered as a study location. Study age groups were extracted as each cohort's mean reported age. The main focus of analysis was the primary outcome measure between pain groups and healthy controls. If brain regions were implicated in imaging studies, reviewers listed relevant regions in the ‘brain regions’ category.

On preliminary analysis of the first 50 studies, most were cross‐sectional. Therefore, the National Institute of Health toolkit for assessing cross‐sectional studies was used to analyse study quality [47, 48]. For the overall quality rating, answers to each of the 11 questions in the assessment were recoded as ‘Yes’ = 1; ‘No’ = ‐1; or ‘Cannot determine/not available or not reported’ = 0. If the sum of these scores was ≤ the overall study quality was rated ‘poor’; 1–5 it was rated ‘fair’; and ≥ 6 it was rated ‘good’. Risk of bias assessment was conducted by two independent reviewers at study level, and consensus was resolved by a third reviewer. Reasons for exclusion at the level of full‐text screening were recorded. Resulting data were exported for further analysis using Microsoft Excel (Microsoft Corp., Redmond, WA, USA) and R Statistical Software (version 4.2.2; R Core Team, Vienna, Austria).

Results were synthesised and presented based on brain health outcomes measured. Chronic pain conditions were grouped into 15 phenotypes based on location or pain condition. Where the main study finding was that group differences did not reach statistical significance, the study was classed as ‘no difference’. For brain region analysis, individual locations were checked for duplication or misspellings and analysed with descriptions of laterality (right, left and bilateral) included and excluded.

## Results

**Figure 1:**
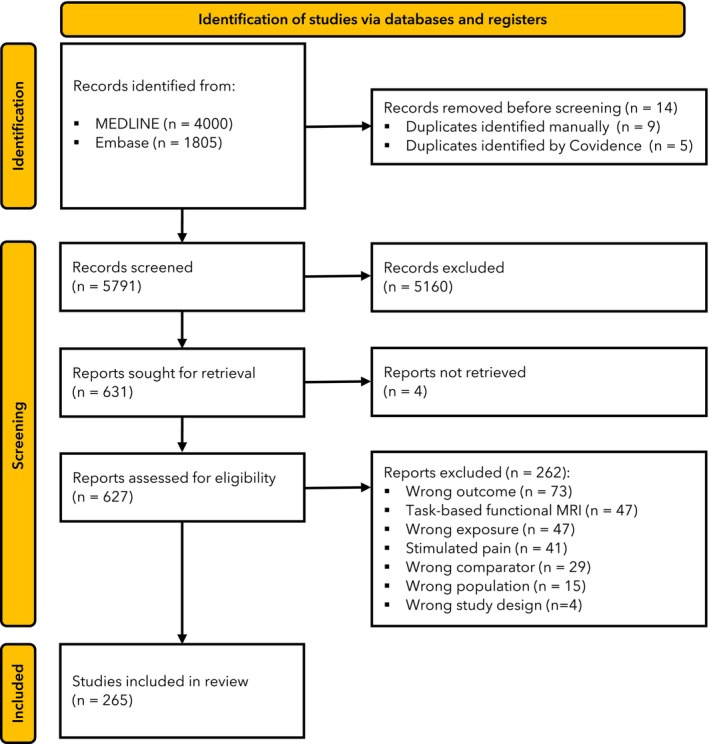
Study flow diagram. MRI, magnetic resonance imaging.

From 5805 identified studies, a full text review was completed for 627 and 365 studies were included (Fig. 1). Publication date ranged from 2004 to 2024, with most published after 2019 (194 studies, 54.5%, online Supporting Information Figure S1). The number of studies increased almost 40‐fold within 20 years. Most studies were conducted in China (96, 26.3%), the USA (77, 21.1%) and Germany (35, 9.6%) (online Supporting Information Figure S2 and Table S1). One study was from Africa (Egypt) and 11 were from South America (10 from Brazil and one from Argentina). Most were cross‐sectional studies, where between‐group outcomes were compared at a single time point (325 studies, 90.1%). There were 35 (9.6%) cohort studies, 4 (1.1%) case–control studies and 1 randomised controlled trial (0.3%).

There was a wide range of pain locations studied (online Supporting Information Table S2). Chronic back pain was the most studied pain phenotype (55 studies, 15.1%), of which most (52/55) investigated chronic lower back pain. The second most studied phenotype was fibromyalgia (40 studies, 11.0%) and the third was studies that considered multisite pain or different groups of pain such as knee, hip, and back pain (39 studies, 9.9%). Pain phenotypes were determined using a range of methods spanning formal diagnostic criteria to self‐report within each phenotype category. For example, of the 15 studies considering complex regional pain syndrome (CRPS), two included upper limb CRPS only, two included type 1 CRPS only and one included patients with CRPS with a pain severity > 20 out of 100 on a visual analogue scale.

Study sample sizes ranged from 5 per group (pain and healthy controls) to 10,984 per group, with the median group size per study of 30 for the pain group and 29 for the control group. Between studies, the number in the control group ranged from 266 fewer to 98,755 more than the pain group. Controls were usually age‐ and sex‐matched to the pain group.

Participant age tended toward a middle‐aged population, with 153 studies (41.9%) investigating participants with a mean age of 31–50 y. There were 87 studies (23.8%) that considered a mean age of 51–70 y; five (1.4%) that investigated individuals aged > 71 y; and 23 (6.3%) that investigated individuals aged 18–30 y. The remaining 96 studies (26.3%) investigated participants with a mixture of ages, mainly 31–70 y (43 studies, 11.8%).

Of 37 possible outcomes included in the search strategy the following were used as primary outcomes in the included studies: structural, functional or diffusion imaging; brain age gap; transcranial magnetic stimulation; serum τ; serum BDNF; and EEG.

Imaging measures were the most common primary method of measuring brain health (324 studies, 88.8%), of which 127 (34.8%) used only the amplitude of low‐frequency fluctuations or regional homogeneity measured during resting state functional MRI, and 31 studies (8.5%) used resting state functional MRI measures in combination with other MRI measures. Structural MRI parameters, specifically grey matter volumes including cortical thickness measurements, were the second most used outcome measure for measuring brain health: 86 studies (23.6%) used it alone; and 47 studies (12.8%) used it in combination with other MRI measures. Three studies (0.8%) investigated white matter hyperintensities exclusively. Diffusion MRI was the third most used modality (49 studies, 13.4%), specifically fractional anisotropy and mean diffusivity. Transcranial magnetic stimulation was used in four studies (1.1%), and cerebral blood flow derived from fMRI was used in eight (2.2%). Other non‐imaging primary brain health outcomes were: EEG (27 studies, 7.4%); BDNF levels (11 studies, 3.0%); cognitive testing (one study, 0.3%); the Beck depression score (one study, 0.3%); and serum τ and amyloid β levels (one study, 0.3%).

Cognitive and mental health tests were used as secondary outcome measures in 10 (2.7%) and 76 (20.8%) studies, respectively. The Mini Mental status examination was the cognitive measure used most (six studies, 1.6%) and the Beck Depression Inventory was the mental health measure used most (48 studies, 13.2%).

Median (IQR [range]) quality scores of the included studies were 3 (1–4 [‐3–10]). Fifty‐eight studies (15.9%) were poor; 276 (75.6%) were fair; and 31 were good (8.5%) (online Supporting Information Table S3). Main areas that reduced study quality related to insufficient information to determine whether the participation rate of eligible persons was at least 50% of the potential study population; subjects were recruited from similar populations in the same period; and whether outcome assessors were blinded to the exposure status of participants. One study (0.3%) reported blinding radiologists to the clinical status of participants. Most studies did not justify their sample size or examine different levels of pain related to the outcome and did not adjust results for potential confounding variables. Overall, there was a moderately high risk of bias from all included studies, as 91.5% (334) were rated ‘fair’ or ‘poor’.

Magnetic resonance imaging findings for brain health in chronic pain are detailed in online Supporting Information Tables S4–S26 by pain phenotype and outcome measure. Grey matter volume differences between chronic pain and healthy controls were examined in 132 studies (36.2%). Of these, 89 (67.4%) found that patients with chronic pain had smaller grey matter volumes across different regions of the brain; 27 (20.5%) found mixed results with smaller and larger volumes in different regions within the brain for chronic pain patients; 12 (9.0%) found larger volumes in patients with chronic pain; and five (3.8%) found no difference between the two groups. From the 127 studies that found a difference in grey matter volumes between groups, 279 specific locations in the brain were identified for group differences separated by laterality (left, right or bilateral) or 97 specific locations once laterality was removed (online Supporting Information Figure S3). The 10 most common areas implicated were the anterior cingulate cortex (42 studies, 33.1%); insula (39 studies, 31.0%); thalamus (27 studies, 21.4%); primary motor cortex (22 studies, 17.3%); hippocampus (22 studies, 17.3%); amygdala (18 studies, 14.2%); cerebellum (17 studies, 13.4%); primary somatosensory cortex (16 studies, 12.6%); temporal lobe (16 studies, 12.6%); and anterior insula (14 studies, 11.0%).

White matter hyperintensity burden alone was investigated by three studies [49, 50, 51]; all showed higher white matter hyperintensities in the chronic pain group and one study showed no white matter hyperintensities in the control group [49]. Lesions were located mainly across the frontal and parietal lobes near the thalamus and cingulate.

Resting state functional MRI was used in 160 studies (43.8%), of which 71 (44.4%) found a mixture of increased and decreased functional connectivity, regional homogeneity or amplitude of low frequency fluctuations in different brain regions between patients with chronic pain and controls. Patients in the chronic pain group showed higher connectivity in 45 studies (28.1%) and lower connectivity in 39 (24.4%). In five studies (3.1%), no difference was seen between the chronic pain and control groups. Resting state brain activity differed in 234 locations in the brain before separating for laterality (left, right or bilateral) and 112 locations after laterality was removed (online Supporting Information Figure S4). The top 10 brain regions most implicated in these studies were the anterior cingulate cortex (29 studies, 34.5%); insula (27 studies, 32.1%); thalamus (22 studies, 26.2%); posterior cingulate cortex (19 studies, 22.7%); sensorimotor network (13 studies, 15.5%); medial prefrontal cortex (13 studies, 15.5%); default mode network (12 studies, 14.3%); periaqueductal grey (12 studies, 14.3%); hippocampus (11 studies, 13.1%); and prefrontal cortex (10 studies, 11.9%).

Magnetic resonance imaging‐derived brain age and brain age gap were measured in seven studies (1.9%). All studies derived brain age using data from structural MRI parameters. Brain age or brain age gap was higher in the chronic pain group for patients with: chronic lower back pain (1.8 months older per chronological year of life); trigeminal neuralgia (6.5 y older); osteoarthritis (9.8 y older); mixed pain cohorts (1.7 y older); and high impact chronic knee pain (3.25 y older than low impact chronic knee pain) [52, 53, 54, 55, 56, 57]. One study found no difference in predicted brain age where patients had chronic non‐cancer pain [58]. One study observed increased brain age in females compared to males across both the pain group and control group (between 5 and 10 y older across different pain phenotypes) [56]. One study not only compared those patients with chronic knee pain with a control group but considered the impact of using non‐pharmacological methods of analgesia for those with chronic pain on brain health [55]. They observed a higher brain age in patients with chronic knee pain who did not use non‐pharmacological methods of analgesia regularly, such as massage (2.8 y older).

Eight studies (2.2%) investigated cerebral blood flow. Two studies found no difference in cerebral blood flow between groups [59, 60]; one study found a reduction in cerebral blood flow in specific regions such as the default mode network [61]; three studies found an increase in cerebral blood flow in regions such as the thalamus and insula [62, 63, 64]; and three studies found increased cerebral blood flow in certain areas of the brain and a reduction in others within patients in the chronic pain group compared with controls [65, 66, 67].

Four studies (1.1%) investigated transcranial magnetic stimulation and showed mixed results. One study showed no difference between patients with chronic pain and controls [68]; one found increased rest motor thresholds on both sides in patients with fibromyalgia [69]; one found lower centre of gravity in the primary motor cortex in patients with back pain [70]; and one found a reduction of intracortical inhibition in patients with neuralgia in the contralateral hemisphere to the pain [71].

Considering non‐imaging findings for brain health in chronic pain, one study investigated differences in cognitive outcomes between chronic pain and control groups [72]. It found that people with burning mouth syndrome had statistically significant impairments in most cognitive domains including trail making tests and Mini Mental State Examination compared with controls. Twelve studies (3.3%) investigated differences in biological markers such as serum τ and Aβ and serum BDNF between patients with chronic pain and controls across a range of pain conditions. One study found higher serum τ and Aβ in patients with fibromyalgia compared with controls [73]; and (of the remaining 11 studies that investigated serum BDNF levels in serum) one found no difference in BDNF levels between groups [74]; one found lower BDNF levels in patients with chronic pain [75]; and nine found higher levels of BDNF in chronic pain groups compared with control groups.

Twenty‐seven studies (7.4%) considered EEG as the primary study outcome. One study found no difference between chronic pain and control groups [76]; nine found reductions in power density, connectivity or decreases in β, α, θ or δ ranges in the chronic pain groups; 13 found increases in power densities, connectivity or increases in α, γ, δ and β activity ranges in the chronic pain groups; and four found mixed results, with higher activity in some ranges and lower activity in others in patients with chronic pain. No included studies utilised outcomes from the mental health domain as the primary outcome.

Regarding phenotyping pain, 119 distinct pain conditions or combinations were studied in the 365 included studies. We categorised studies into 15 groups based on pain phenotype, pain location or condition. These included: abdominal pain; back pain; CRPS; diabetic neuropathy; facial pain; fibromyalgia; headache; musculoskeletal pain; mixed pain groups; neck and shoulder pain; neuropathic pain; pelvic pain; postherpetic neuralgia; somatoform pain disorder; and ‘other’ types of pain that included cancer pain, sickle cell disease‐associated pain and Parkinson's disease‐associated pain.

**Figure 2:**
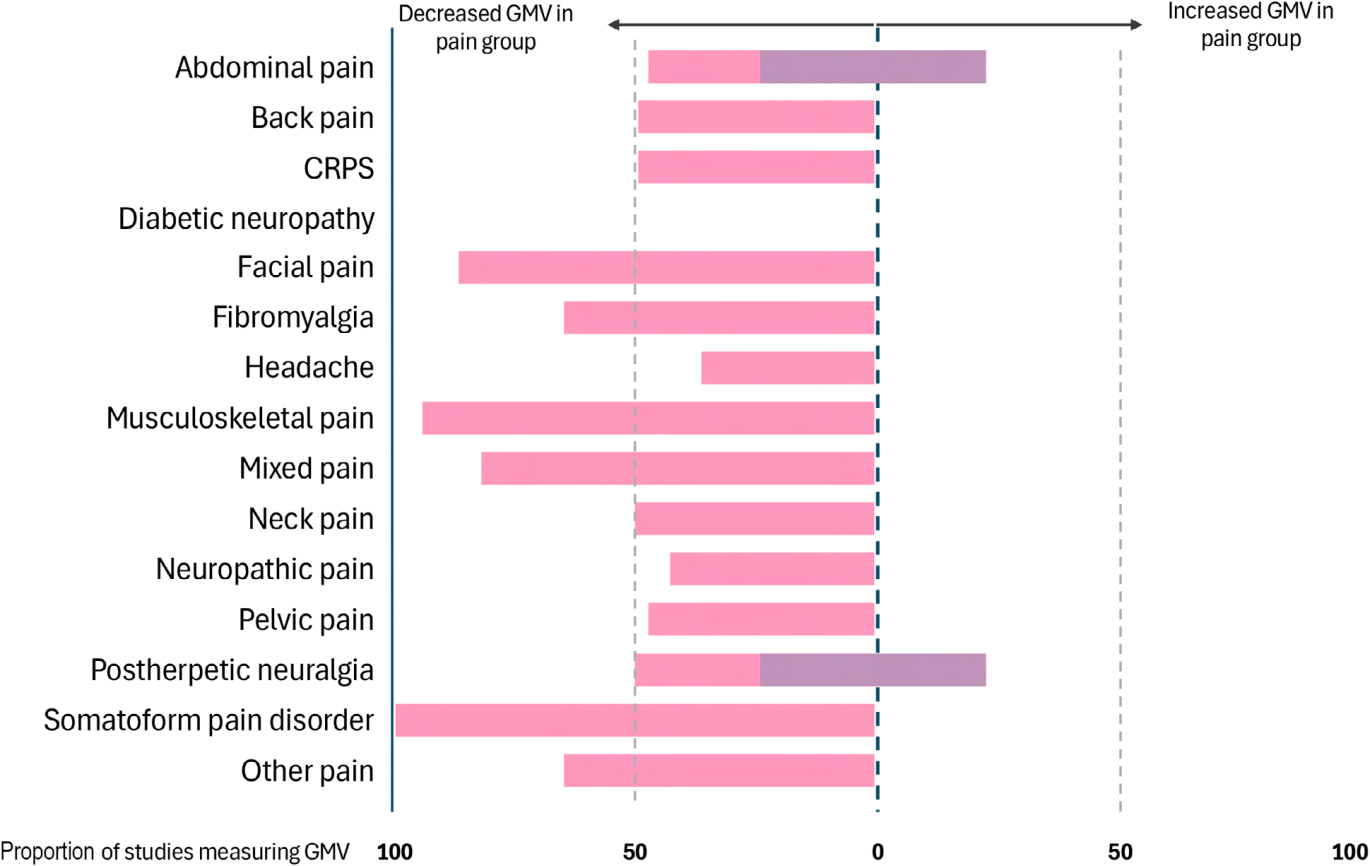
The direction of grey matter volume difference between individuals with chronic pain and controls that was found by the highest proportion of studies within each pain phenotype. Pink bars, decreased grey matter volumes; purple bars, mixed increased and decreased grey matter volumes. GMV, grey matter volume; CRPS, complex regional pain syndrome.

**Figure 3:**
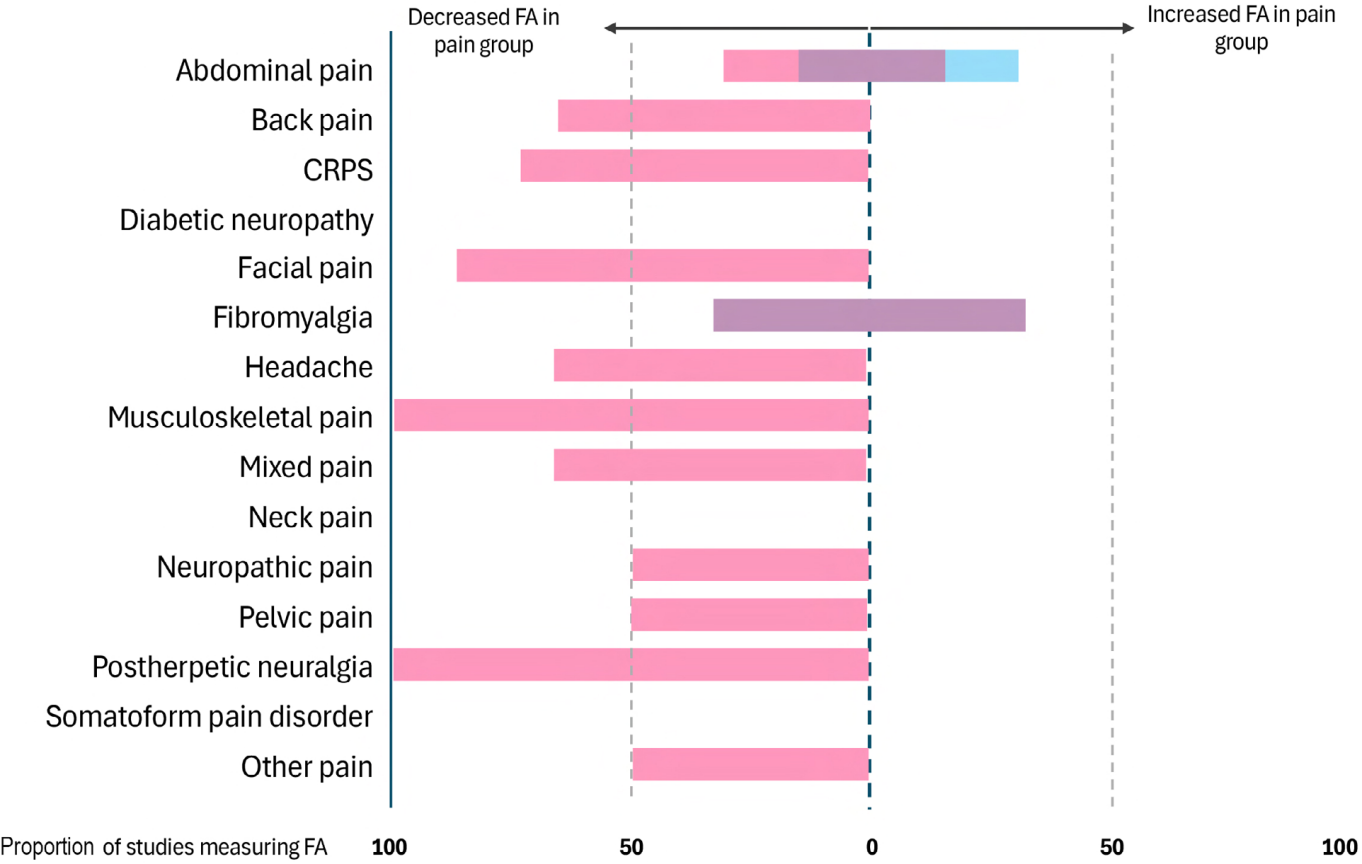
The direction of fractional anisotropy difference between individuals with chronic pain and controls that was found by the highest proportion of studies within each pain phenotype. Pink bars, decreased fractional anisotropy; purple bars, mixed increased and decreased fractional anisotropy; blue bars, increased fractional anisotropy. FA, fractional anisotropy; CRPS, complex regional pain syndrome.

**Figure 4:**
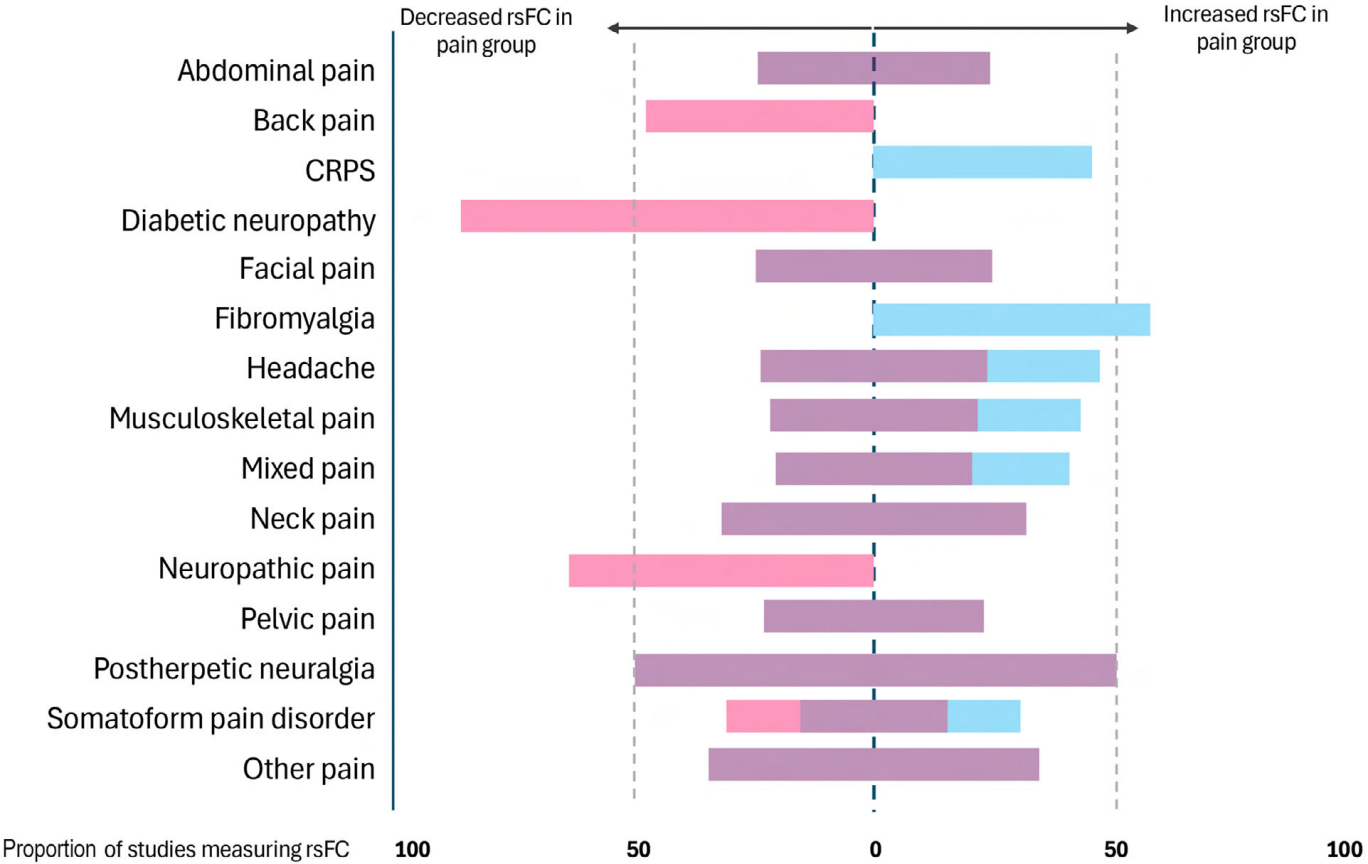
The direction of resting state functional connectivity between individuals with chronic pain and controls that was found by the highest proportion of studies within each pain phenotype. Pink bars, decreased resting state functional connectivity; purple bars, mixed increased and decreased resting state functional connectivity; blue bars, increased resting state functional connectivity. rsFC, resting state functional connectivity; CRPS, complex regional pain syndrome.

For structural MRI studies, smaller grey matter volumes were found in the chronic pain group compared with healthy controls in most studies. In abdominal pain and postherpetic neuralgia, an equal number of studies found mixed grey matter volume findings (larger grey matter volumes in some regions and smaller grey matter volumes in others) (Fig. 2). Fractional anisotropy was found to be lower in the chronic pain group compared with the control group in all conditions except fibromyalgia and mixed pain where it was mixed higher and lower, and abdominal pain and ‘other’ pain where there was one study in each direction (Fig. 3). Resting state functional MRI results were more mixed, with higher connectivity in some brain regions was seen in abdominal pain, CRPS and fibromyalgia; lower connectivity was seen in back pain, diabetic neuropathy and neuropathic pain; high or mixed was seen in headache, musculoskeletal pain and mixed pain groups; and mixed in all other groups (Fig. 4).

The insula was one of the top five brain regions implicated more than once in all MRI modality studies (13/15 chronic pain categories), followed by the anterior cingulate cortex (12/15) and thalamus (6/15) (online Supporting information Table S4). Each pain phenotype had a different combination of areas implicated.

## Discussion

This review found that individuals with chronic pain exhibit altered brain health across multiple domains, including MRI, EEG and serum BDNF levels, compared with healthy controls. The overall trend across pain phenotypes suggests that chronic pain is associated with reduced grey matter volumes; lower white matter integrity; older brain age; regionally variable resting‐state functional connectivity; and elevated serum BDNF. Structural MRI studies most frequently identified differences in the: anterior and middle cingulate cortices; insula; thalamus; primary motor cortex; hippocampus; amygdala; cerebellum; primary somatosensory cortex; and temporal lobe. Differences in resting state functional MRI were primarily reported in the insula; anterior; middle and posterior cingulate cortices; thalamus; cerebellum; postcentral gyrus; precuneus; amygdala; and middle prefrontal cortex.

Findings from our review suggest that chronic pain may affect the brain in ways that mirror structural and functional changes seen in Alzheimer's disease. Specifically, 17.3% of structural MRI studies reported hippocampal atrophy in individuals with chronic pain compared with controls. Additionally, most studies identified lower grey matter volumes or cortical thinning in regions commonly implicated in Alzheimer's disease [77, 78]. Grey matter atrophy and cortical thinning [79], particularly in the hippocampus and prefrontal cortex, are strongly associated with Alzheimer's disease [80, 81].

Our review also found that chronic pain is associated with lower fractional anisotropy levels in the white matter tracts, including the cingulum and regions spanning the frontal, temporal and parietal lobes (Fig. 3). Decreased fractional anisotropy is thought to reflect reduced white matter integrity, as diffusion becomes less directionally constrained, more similar to free water [42]. Similarly, Alzheimer's disease is characterised by lower fractional anisotropy in the same regions [80], particularly in the cingulum and fornix [80], which are known to support memory and task performance, as shown in animal models [82, 83]. In addition, most pain conditions studied showed altered resting state function MRI connectivity in regions implicated in Alzheimer's disease (Fig. 4). Patients with Alzheimer's disease exhibit disrupted connectivity within the default mode network, including the medial prefrontal cortex; cingulate cortices; precuneus; parietal cortex; and medial temporal lobe, notably the hippocampus [80, 81].

While these findings support the hypothesis that chronic pain may affect brain health in a pattern similar to Alzheimer's disease, several limitations must be acknowledged. First, many imaging findings such as hippocampal atrophy, while strongly associated with Alzheimer's disease, are not disease‐specific and have been observed in other neurodegenerative diseases such as epilepsy [84]. Second, it remains unclear whether structural MRI findings reflect underlying Alzheimer's disease pathophysiological processes, such as τ and amyloid deposition. Third, technical and methodological confounders including head motion and table position [85] were not controlled for consistently across studies. Finally, many included studies had a moderate to high risk of bias, limiting the strength of conclusions drawn (online Supporting Information Table S3).

While many findings in this review suggest brain health in chronic pain resembles that observed in Alzheimer's disease, some studies reported results that diverge from typical Alzheimer's disease or other dementia subtypes. Notably, 16 studies (12% of all structural MRI studies) reported increased grey matter volumes in the pain group compared with controls. Increased grey matter volume is generally associated with neuronal growth and plasticity, commonly observed in individuals who have longer sleep durations [86] or exercise regularly [87], and in children who have been breast‐fed [88, 89]. Some studies have found increased grey matter volumes in patients with neuropsychiatric long‐COVID‐19 syndrome [90] and children with obsessive compulsive disorder [91]. These findings may reflect compensatory neural adaptations or early‐stage plasticity in response to chronic pain. Moreover, some of these studies involved younger populations (e.g. facial pain and headache cohorts aged 18–35 y), which could account for the observed apparent neuroanatomical increases.

Similarly, 9/11 studies found higher serum BDNF levels in pain groups compared with controls. This contrasts with Alzheimer's disease, where BDNF levels are usually reduced [27, 92, 93]. Brain‐derived neurotrophic factor plays an important role in maintaining neuronal health and synaptic plasticity and reduces Aβ accumulation and τ phosphorylation and subsequent cognitive decline [93]. The highest BDNF levels were observed in patients with fibromyalgia [94, 95], although elevated levels were observed across multiple pain conditions including endometriosis and knee osteoarthritis. One hypothesis is that chronic pain induces synaptic hyperactivity and neuroplastic remodelling, prompting neurons to increase BDNF production to support ongoing structural changes [96]. However, few studies in this review characterised the duration of chronic pain, making it difficult to determine whether higher BDNF levels reflect acute neuroadaptive responses or long‐term compensatory mechanisms.

Even when studies were grouped by pain condition or location, the brain health outcomes remained heterogeneous, particularly in the directionality (higher, lower or mixed) and the specific brain regions implicated in group differences. For example, studies on irritable bowel syndrome and postherpetic neuralgia pain yielded mixed structural MRI findings (Fig. 2), with some studies reporting smaller grey matter volumes and others reporting no significant differences or mixed results. The variability in irritable bowel syndrome findings may stem from its origin in neuroplastic changes along the brain–gut axis [97, 98], a system recognised increasingly as central to the pathophysiology of functional gastrointestinal disorders. In the case of postherpetic neuralgia, variability in brain health may be related to different degrees of peripheral neuroinflammation leading to axonal loss in various dermatomes [99]. Interestingly, both irritable bowel syndrome and postherpetic neuralgia are pain conditions with strong ties to microbial influence, which may explain the observed variability in grey matter findings.

Despite the number of included studies in this review, there remain significant gaps in the literature. Almost all the studies were cross‐sectional, of small sample size and mostly conducted in China, western Europe or North America, with participants mainly Chinese or White Caucasian in ethnic origin. Clearly, this does not reflect the global prevalence of Alzheimer's disease, other dementia subtypes or chronic pain [100, 101, 102]. Some studies found that females with chronic pain had worse brain health than males. Both chronic pain and dementia are more prevalent in women than men [36, 37, 38], and there are sex differences in genetic and immune modulation of pain [39, 40, 41]. Future work should analyse sex‐specific differences in brain health across different pain conditions.

While an association between chronic pain and poorer brain health has been identified from this review, the causal direction of this relationship and mechanisms that might underlie it remain unknown. Most studies did not use multimodal imaging to assess brain health, and few performed statistical adjustments for known confounders. Future work on evaluating brain health should employ multimodal imaging techniques. Novel techniques utilising positron emission tomography to trace Aβ peptides or the τ burden in the brain specifically have gained popularity as biomarkers for early Alzheimer's disease [80], and could be applied to patients with chronic pain. Careful consideration and adjustment of results based on imaging and other known confounders could provide a clearer picture of the true relationship between chronic pain and brain health.

While the definition of chronic pain we used has practical utility, the variation in brain health patterns across different pain phenotypes in this review highlights the heterogeneity of pathophysiology and sequelae between them. Pain phenotypes were characterised inadequately in most studies, and few performed subgroup analyses or stratified pain based on severity or impact. Notably some categorisation of phenotypes such as somatoform pain syndromes are now absent from international classification system and this could have also influenced subgroup analysis. Future work should move away from the ‘one size fits all’ assumption for chronic pain to include larger sample sizes of patients with more detailed phenotyping of pain and consideration for stratification based on severity or impact, or clearer classifications using objective and repeatable diagnostic tools where possible.

This systematic review found that chronic pain is consistently associated consistently with altered brain health across a range of imaging and non‐imaging outcomes. Many of the key findings, including lower grey matter volumes, fractional anisotropy and altered resting‐state functional connectivity in the hippocampus, cingulate cortices and thalamus, mirror early neurobiological changes observed in Alzheimer's disease. Importantly, different chronic pain phenotypes were associated with distinct patterns of brain health, suggesting that pain type and possibly duration may influence the specific brain regions affected. These findings underscore the potential role of chronic pain as a modifiable risk factor for neurodegeneration and highlight the need for further longitudinal and mechanistic studies to clarify causal pathways and inform targeted interventions.

## Supporting information


Plain Language Summary.

**Figure S1.** Number of studies published by year.
**Figure S2.** Geographical distribution of studies on brain health in chronic pain.
**Figure S3.** Brain regions implicated most commonly in structural and diffusion MRI studies of brain health in chronic pain.
**Figure S4.** Brain regions implicated most commonly in resting state functional MRI studies of brain health in chronic pain.

**Table S1.** Location of study.
**Table S2.** Pain phenotypes.
**Table S3.** Quality assessment.
**Table S4.** Top five brain regions that differ between different chronic pain phenotypes vs. healthy controls.
**Table S5.** Abdominal pain results.
**Table S6.** Chronic back pain structural MRI results.
**Table S7.** Chronic low back pain resting state functional MRI results.
**Table S8.** Complex regional pain syndrome brain health results.
**Table S9.** Diabetic peripheral neuropathy results.
**Table S10.** Temporomandibular disorder brain health findings.
**Table S11.** Trigeminal neuralgia brain health findings.
**Table S12.** Fibromyalgia structural and diffusion MRI findings.
**Table S13.** Fibromyalgia resting state functional MRI findings.
**Table S14.** Fibromyalgia resting state electroencephalography study findings.
**Table S15.** Headache structural and diffusion MRI study findings.
**Table S16.** Headache resting‐state functional MRI study findings.
**Table S17.** Hip and knee pain MRI study findings.
**Table S18.** Chronic musculoskeletal pain study findings.
**Table S19.** Mixed pain MRI study findings.
**Table S20.** Mixed pain non‐imaging study findings.
**Table S21.** Neck pain study findings.
**Table S22.** Neuropathic pain study findings.
**Table S23.** Pelvic pain study findings.
**Table S24.** Postherpetic neuralgia study findings.
**Table S25.** Somatoform pain syndrome study findings.
**Table S26.** Other pain study findings.

**Appendix S1.** Search strategy.
**Appendix S2.** Data extraction form.
