# Supplementary material for: A systematic review of brain health in adults with chronic pain
Source: Anaesthesia. 2025 Oct 14;81(2):248–62. doi: 10.1111/anae.70021 (PMC12803547; doi:10.1111/anae.70021)
Supplement: Supplementary file 1 — Plain Language Summary. [file ANAE-81-248-s002.docx]

**Plain Language Summary**
We reviewed all studies written in English on adults with long-term (chronic) pain and brain health. We searched two large databases up to 1 November 2024. Studies had to compare people with chronic pain to people without pain. We collected their results and checked each study for quality. Some research has shown that people with chronic pain have a higher chance of a dementia diagnosis in later life. We do not fully know how chronic pain links to brain changes or to faster brain ageing. By bringing many studies together, we aimed to map the main brain changes linked to chronic pain to improve care and find targets for treatment. We found 365 suitable studies to compare. Most were small studies done at a single time point in middle-aged adults, often from China or the United States, and many had moderate to high risk of being unfair (biased) because of the way the studies were done. Magnetic resonance imaging (MRI) brain scans were the most common way to measure brain health. On MRI scans, people with chronic pain often had patterns that were like the patterns seen in people with Alzheimer’s disease: less grey brain tissue, weaker white brain tissue and signs of older brain age in places such as the hippocampus (the memory centre of the brain). There were also changes in brain chemical levels and brain activity levels in people with chronic pain compared to those without chronic pain. The findings suggest wide brain changes in people with chronic pain. Larger, better-designed studies are needed to understand these links more clearly.
