# Supplementary material for: A systematic review of brain health in adults with chronic pain
Source: Anaesthesia. 2025 Oct 14;81(2):248–62. doi: 10.1111/anae.70021 (PMC12803547; doi:10.1111/anae.70021)

## **Figure S1:** Number of studies published by year.

## **Figure S2:** Geographical distribution of studies on brain health in chronic pain. A world map of study locations by country, with a heat map for proportion of studies from each location, where a higher colour gradient represents countries with a higher percentage of studies conducted there.

## **Figure S3:** Brain regions most commonly implicated in structural and diffusion MRI studies of brain health in chronic pain


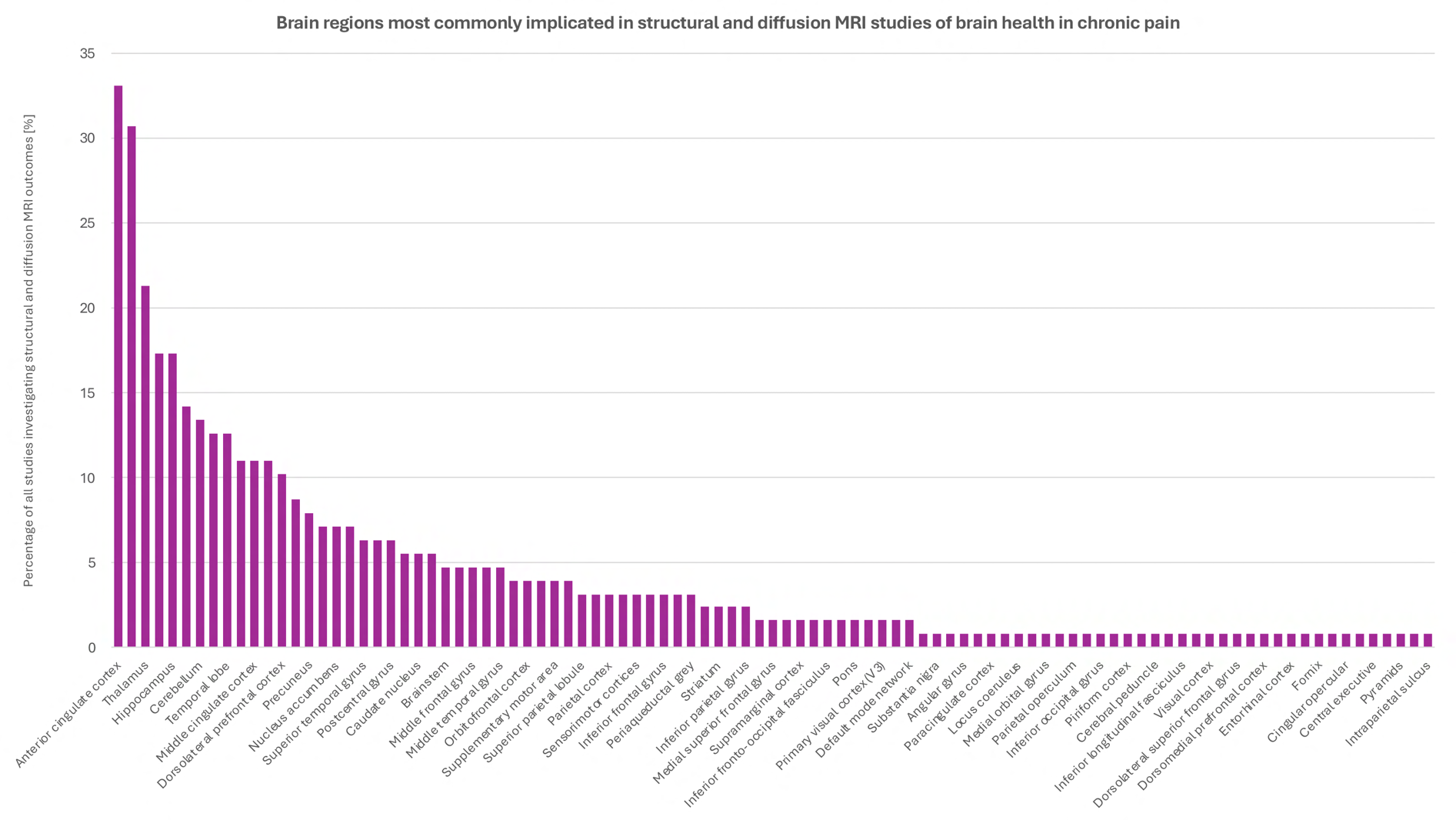


## **Figure S4:** Brain regions most commonly implicated in resting state functional MRI studies of brain health in chronic pain


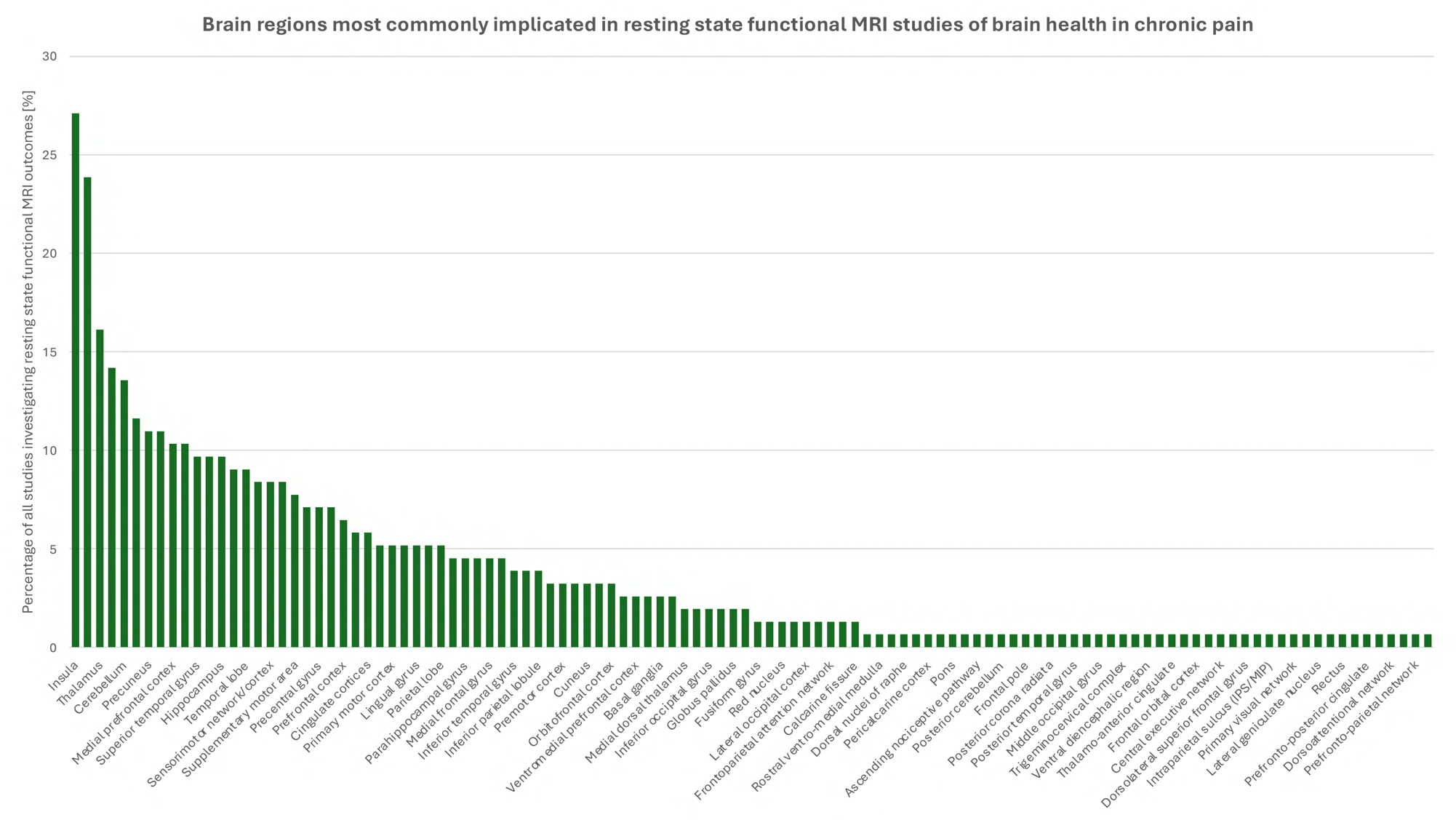

Supplement: Supplementary file 2 — Figure S1. Number of studies published by year. Figure S2. Geographical distribution of studies on brain health in chronic pain. Figure S3. Brain regions implicated most commonly in structural and diffusion MRI studies of brain health in chronic pain. Figure S4. Brain regions implicated most commonly in resting state functional MRI studies of brain health in chronic pain. [file ANAE-81-248-s004.docx]
