# Supplementary material for: A systematic review of brain health in adults with chronic pain
Source: Anaesthesia. 2025 Oct 14;81(2):248–62. doi: 10.1111/anae.70021 (PMC12803547; doi:10.1111/anae.70021)
Supplement: Supplementary file 3 — Table S1. Location of study. Table S2. Pain phenotypes. Table S3. Quality assessment. Table S4. Top five brain regions that differ between different chronic pain phenotypes vs. healthy controls. Table S5. Abdominal pain results. Table S6. Chronic back pain structural MRI results. Table S7. Chronic low back pain resting state functional MRI results. Table S8. Complex regional pain syndrome brain health results. Table S9. Diabetic peripheral neuropathy results. Table S10. Temporomandibular disorder brain health findings. Table S11. Trigeminal neuralgia brain health findings. Table S12. Fibromyalgia structural and diffusion MRI findings. Table S13. Fibromyalgia resting state functional MRI findings. Table S14. Fibromyalgia resting state electroencephalography study findings. Table S15. Headache structural and diffusion MRI study findings. Table S16. Headache resting‐state functional MRI study findings. Table S17. Hip and knee pain MRI study findings. Table S18. Chronic musculoskeletal pain study findings. Table S19. Mixed pain MRI study findings. Table S20. Mixed pain non‐imaging study findings. Table S21. Neck pain study findings. Table S22. Neuropathic pain study findings. Table S23. Pelvic pain study findings. Table S24. Postherpetic neuralgia study findings. Table S25. Somatoform pain syndrome study findings. Table S26. Other pain study findings. [file ANAE-81-248-s003.docx]

## **Table S1: Location of study.**

This is a frequency table of study countries with percentage of the 365 included studies in this review.

| Country | n | Percentage |
| --- | --- | --- |
| China | 96 | 26.3 |
| United States | 77 | 21.1 |
| Germany | 35 | 9.6 |
| Canada | 22 | 6.0 |
| Italy | 13 | 3.6 |
| South Korea | 12 | 3.3 |
| Spain | 12 | 3.3 |
| United Kingdom | 12 | 3.3 |
| Australia | 11 | 3.0 |
| Brazil | 10 | 2.7 |
| Taiwan | 10 | 2.7 |
| Japan | 9 | 2.5 |
| Switzerland | 9 | 2.5 |
| Belgium | 8 | 2.2 |
| Netherlands | 5 | 1.4 |
| Denmark | 4 | 1.1 |
| Turkiye | 4 | 1.1 |
| Sweden | 3 | 0.8 |
| Finland | 2 | 0.5 |
| India | 2 | 0.5 |
| Iran | 2 | 0.5 |
| Russia | 2 | 0.5 |
| Argentina | 1 | 0.3 |
| Austria | 1 | 0.3 |
| Egypt | 1 | 0.3 |
| France | 1 | 0.3 |
| Hungary | 1 | 0.3 |
| Portugal | 1 | 0.3 |

## **Table S2: Pain phenotypes.**

This is a frequency table of pain phenotypes used in the 365 studies included in this review.

| Pain group or location | n | Percentage |
| --- | --- | --- |
| Chronic back pain | 55 | 15.1 |
| Fibromyalgia | 40 | 11.0 |
| Other (mixed) | 39 | 9.9 |
| Trigeminal neuralgia (TN) | 31 | 8.5 |
| Pelvic pain | 24 | 6.5 |
| Migraine | 21 | 5.8 |
| Complex regional pain syndrome (CRPS) | 15 | 4.1 |
| Facial pain | 14 | 3.8 |
| Neuropathic pain | 14 | 3.8 |
| Neck pain | 10 | 2.7 |
| Abdominal pain | 10 | 2.7 |
| Somatoform pain disorder | 9 | 2.5 |
| Irritable bowel syndrome (IBS) | 8 | 2.2 |
| Postherpetic neuralgia | 8 | 2.2 |
| Knee pain + osteoarthritis | 7 | 1.9 |
| Shoulder pain | 6 | 1.6 |
| Burning mouth syndrome | 6 | 1.6 |
| Musculoskeletal pain | 6 | 1.6 |
| Chronic back pain + mixed | 5 | 1.4 |
| Diabetic neuropathy | 5 | 1.4 |
| Knee pain | 5 | 1.4 |
| Cervical spondylosis | 4 | 1.1 |
| Cluster headache | 3 | 0.8 |
| Neck pain + shoulder pain | 3 | 0.8 |
| Non-migraine headache | 3 | 0.8 |
| Cancer pain | 2 | 0.5 |
| Sickle cell disease | 2 | 0.5 |
| Hip pain | 2 | 0.5 |
| Hand pain + osteoarthritis | 1 | 0.3 |
| Phantom limb pain | 1 | 0.3 |
| Rheumatoid arthritis | 1 | 0.3 |
| Vulvodynia | 1 | 0.3 |

## **Table S3: Quality assessment**

This table shows the consensus answers to the 11 questions of the NIH Toolkit for Assessing Cross Sectional Studies for all the studies included in this review. Green boxes represent that the majority of studies were at low risk of bias for this parameter; yellow boxes represent that the majority of studies were at moderate risk of bias for this parameter, and red boxes represent that the majority of studies were at high risk of bias for this parameter.

| Assessment question | No (%) | Cannot determine/ not reported/ not available (%) | Yes (%) |
| --- | --- | --- | --- |
| Was the research question or objective in this paper clearly stated? | 14 (3.8) | 2 (0.5) | 349 (95.6) |
| Was the study population clearly specified and defined? | 41 (11.2) | 12 (3.3) | 312 (85.5) |
| Was the participation rate of eligible persons at least 50%? | 23 (6.3) | 324 (88.8) | 18 (4.9) |
| Were all the subjects selected or recruited from the same or similar populations (including the same time period?) Were inclusion and exclusion criteria for being in the study respecified and applied uniformly to all participants? | 26 (7.1) | 185 (50.7) | 154 (42.2) |
| Was a sample size justification, power description, or variance and effect estimates provided? | 312 (89.0) | 16 (4.4) | 24 (6.6) |
| For the analyses in this paper, were the exposure(s) of interest (chronic pain) measured prior to the outcome(s) being measured? | 29 (7.9) | 40 (11.0) | 296 (81.1) |
| For exposures that can vary in amount or level, did the study examine different levels of the exposure as related to the outcome (e.g., categories of exposure, or exposure measured as continuous variable)? | 252 (69.0) | 14 (3.8) | 99 (27.1) |
| Were the exposure measures (chronic pain) clearly defined, valid, reliable, and implemented consistently across all study participants? | 24 (6.6) | 70 (19.2) | 271 (74.2) |
| Were the outcome measures (dependent variables) clearly defined, valid, reliable, and implemented consistently across all study participants? | 0 (0.0) | 2 (0.5) | 363 (99.5) |
| Were the outcome assessors blinded to the exposure status of participants? | 0 (0.0) | 361 (99.7) | 1 (0.3) |
| Were key potential confounding variables measured and adjusted statistically for their impact on the relationship between exposure(s) and outcome(s)? | 242 (66.3) | 54 (14.8) | 69 (18.9) |

## **Table S4: Top five brain regions that differ between different chronic pain phenotypes versus healthy controls.**

| **Chronic pain group (n of MRI studies)** | Top five regions implicated more than once in all MRI modality studies | Number of studies (%) | |  |
| --- | --- | --- | --- | --- |
| **Abdominal pain (n=14)** | Insula | 4 | (28.5) | |
|  | Prefrontal cortex | 3 | (21.4) | |
|  | Primary somatosensory cortex | 3 | (21.4) | |
|  | Anterior cingulate cortex | 3 | (21.4) | |
|  | Sensorimotor cortex | 3 | (21.4) | |
| **Back pain (n=44)** | Primary somatosensory cortex | 6 | (13.6) | |
|  | Default mode network | 6 | (13.6) | |
|  | Thalamus | 5 | (11.4) | |
|  | Amygdala | 4 | (9.1) | |
|  | Anterior cingulate cortex | 4 | (9.1) | |
| **Complex regional pain syndrome (CRPS) (n=13)** | Prefrontal cortex | 10 | (76.9) | |
|  | Insula | 4 | (30.8) | |
|  | Anterior cingulate cortex | 3 | (23.1) | |
|  | Primary somatosensory cortex | 3 | (23.1) | |
|  | Corona radiata | 2 | (15.4) | |
| **Diabetic neuropathy (n=5)** | Thalamus | 4 | (80.0) | |
|  | Insula | 3 | (60.0) | |
|  | Anterior cingulate cortex | 3 | (60.0) | |
|  | Postcentral gyrus | 2 | (40.0) | |
|  | Frontal gyrus | 2 | (40.0) | |
| **Facial pain (n=47)** | Thalamus | 9 | (19.5) | |
|  | Insula | 7 | (14.9) | |
|  | Cingulum | 6 | (12.8) | |
|  | Temporal lobe | 5 | (10.7) | |
|  | Medial prefrontal cortex | 5 | (10.7) | |
| **Fibromyalgia (n=28)** | Insula | 11 | (39.3) | |
|  | Anterior cingulate cortex | 8 | (28.6) | |
|  | Posterior cingulate cortex | 6 | (21.4) | |
|  | Amygdala | 5 | (17.9) | |
|  | Supplementary motor area | 4 | (14.3) | |
| **Headache (n=24)** | Insula | 8 | (33.3) | |
|  | Anterior cingulate cortex | 8 | (33.3) | |
|  | Periaqueductal grey | 4 | (16.7) | |
|  | Cerebellum | 4 | (16.7) | |
|  | Prefrontal cortex | 3 | (12.5) | |
| **Musculoskeletal pain (n=27)** | Insula | 6 | (22.2) | |
|  | Anterior cingulate cortex | 6 | (22.2) | |
|  | Hippocampus | 5 | (18.5) | |
|  | Superior temporal gyrus | 4 | (14.8) | |
|  | Prefrontal cortex | 4 | (14.8) | |
| **Mixed pain (n=23)** | Insula | 9 | (39.1) | |
|  | Anterior cingulate cortex | 5 | (21.7) | |
|  | Hippocampus | 5 | (21.7) | |
|  | Sensorimotor cortex | 4 | (17.4) | |
|  | Prefrontal cortex | 4 | (17.4) | |
| **Neck pain (n=18)** | Precuneus | 6 | (33.3) | |
|  | Anterior cingulate cortex | 6 | (33.3) | |
|  | Insula | 5 | (27.8) | |
|  | Middle cingulate cortex | 5 | (27.8) | |
|  | Precentral gyrus | 4 | (22.2) | |
| **Neuropathic pain (n=11)** | Somatosensory cortex | 4 | (36.4) | |
|  | Prefrontal cortex | 3 | (27.3) | |
|  | Thalamus | 3 | (27.3) | |
|  | Insula | 3 | (27.3) | |
|  | Anterior cingulate cortex | 3 | (27.3) | |
| **Pelvic pain (n=23)** | Insula | 7 | (30.4) | |
|  | Thalamus | 4 | (17.4) | |
|  | Hippocampus | 4 | (17.4) | |
|  | Anterior cingulate cortex | 4 | (17.4) | |
|  | Caudate nucleus | 4 | (17.4) | |
| **Postherpetic neuralgia (n=7)** | Cerebellum | 7 | (100) | |
|  | Postcentral gyrus | 4 | (57.1) | |
|  | Insula | 4 | (57.1) | |
|  | Middle frontal gyrus | 3 | (42.9) | |
|  | Visual cortex | 3 | (42.9) | |
| **Somatoform pain disorder (n=6)** | Thalamus | 2 | (33.3) | |
|  | Occipital lobe | 2 | (33.3) | |
| **Other pain (n=8)** | Insula | 3 | (37.5) | |
|  | Anterior cingulate cortex | 3 | (37.5) | |
|  | Parietal cortex | 2 | (25.0) | |

## **Table S5: Abdominal pain results**

| Study | Pain type | Primary outcome measure | Number of pain group patients | Number of healthy controls | Age group(s) of patients | Main finding (pain group vs control) |
| --- | --- | --- | --- | --- | --- | --- |
| Hong 2013[1] | IBS | Cerebral blood flow | 60 | 118 | 18-30y; 31-50y | No difference between groups. Sex differences in power distribution in the insula, amygdala, right hippocampus and anterior insula. |
| Chen 2011[2] | IBS | Fractional anisotropy | 10 | 16 | 18-30y; 31-50y; 51-70y | ↑FA in fornix and external capsule adjacent to right posterior insula |
| Frokjaer 2011[3] | Chronic pancreatitis | Fractional anisotropy | 23 | 14 | 31-50y; 51-70y | ↓FA in the amygdala, cingulate cortex, insula, prefrontal cortex and secondary sensory cortex |
| Ellingson 2013[4] | IBS | Fractional anisotropy | 33 | 93 | 18-30y; 31-50y | ↓FA in globus pallidus and putamen, thalamus, primary and secondary somatosensory motor regions and posterior cingulate white matter bundle  ↑FA in prefrontal white matter regions and corpus callosum |
| Chua 2017[5] | IBS | Grey matter volumes or cortical thickness | 30 | 39 | 31-50y | ↓Cortical thickness in left cuneus, rostral middle frontal cortex, supramarginal cortex, right caudal anterior cingulate cortex, bilateral insula |
| Barazanji 2022[6] | IBS | Grey matter volumes or cortical thickness | 75 | 39 | 18-30y; 31-50y; 51-70y | ↓GMV in insular cortex |
| Frokjaer 2012[7] | Chronic pancreatitis | Grey matter volumes or cortical thickness | 19 | 15 | 31-50y; 51-70y | ↓cortical thickness in secondary somatosensory cortex, prefrontal cortex, frontal cortex, mid cingulate cortex, insula |
| Jiang 2013[8] | IBS | Grey matter volumes or cortical thickness | 90 | 176 | 18-30y; 31-50y | No difference in cortical thickness between groups; difference only in female pain patients vs controls - ↑ cortical thickness in somatosensory and primary motor cortex and ↓ in bilateral subgenual anterior cingulate cortex |
| Ohlmann 2021[9] | Chronic visceral pain, ulcerative colitis (UC), IBS | Grey matter volumes or cortical thickness | 31 UC and 23 IBS = 54 | 44 | 31-50y | ↓GMV in frontal cortex, anterior insula for UC  ↑↓GMV in sensorimotor, central executive and default mode networks for IBS |
| Grinsvall 2021[10] | IBS | Grey matter volumes or cortical thickness | 216 | 138 | 31-50y | ↑ cortical thickness in bilateral postcentral gyri, central sulcus, right subcentral gyrus and sulcus, left precentral gyrus, right nucleus accumbens, left putamen and left thalamus; ↓ cortical thickness of bilateral superior frontal gyrus and sulcus and smaller volume and surface area of posterior part of lateral fissure |
| Prus 2022[11] | Inflammatory bowel disease | Resting state fMRI | 32 | 32 | 31-50y | No structural MRI differences between groups. ↑rsFC of secondary somatosensory cortex within the salience network |
| Chen 2023[12] | Crohn’s disease with abdominal pain | Resting state fMRI | 24 | 28 | 18-30y; 31-50y | ↓rsFC for ventrolateral periaqueductal grey with the precuneus, medial prefrontal cortex (mPFC), orbitofrontal cortex (OFC), angular gyrus and premotor cortex |
| Qi 2020[13] | Functional dyspepsia | Resting state fMRI | 31 | 22 | 31-50y | ↑ALFF in sensorimotor cortex, insula and thalamus |
| Hong 2014[14] | IBS | Resting state fMRI | 48 | 48 | 18-30y; 31-50y | ↑rsFC between anterior insula and left precuneus and frontal regions observed in females only |
| Bao 2016[15] | Crohn’s disease with abdominal pain | Resting state fMRI | 25 | 32 | 18-30y; 31-50y | ↓ReHo in insula, middle cingulate cortex, supplementary motor area and ↑ReHo in the temporal pole |
| deVries 2013[16] | Chronic pancreatitis | EEG | 16 | 16 | 31-50y | ↓peak alpha frequency |
| Olesen 2011[17] | Chronic pancreatitis | EEG | 31 | 15 | 31-50y; 51-70y | ↓EEG rhythmicity |

IBS: Irritable bowel syndrome, fMRI: functional magnetic resonance imaging, EEG: electroencephalography, rsFC: resting state functional connectivity; ALFF: amplitude of low frequency fluctuation; ReHo: regional homogeneity

## **Table S6: Chronic back pain structural MRI results**

| Study | Pain type | Number of pain group patients | Number of healthy controls | Age group(s) of patients | Main finding (pain group vs control) |
| --- | --- | --- | --- | --- | --- |
| Gu 2024[18] | Chronic low back pain | 38 | 38 | 51-70y | ↓cortical thickness in left superior frontal cortex |
| Apkarian 2004[19] | Chronic low back pain | 26 | 26 | 51-70y | ↓GMV in neocortex |
| Schmidt-Wilcke 2006[20] | Chronic low back pain | 18 | 18 | 31-50y | ↓GMV in brainstem and somatosensory cortex |
| Fritz 2016[21] | Chronic low back pain | 111 | 432 | 51-70y | ↓GMV ventrolateral and dorsolateral prefrontal cortex, anterior insula |
| Mansour 2017[22] | Chronic low back pain | 130 (23 subacute and 68 chronic low back pain) | 39 | 31-50y | ↓GMV middle frontal gyrus, no difference in whole brain volume |
| Murray 2021[23] | Chronic low back pain chronically medicated with opioids without any evidence of misuse | 11 CLBP+opioids, 30 CLBP no opioid | 30 | 31-50y; 51-70y | ↓GMV in nucleus accumbens and thalamus |
| Buckalew 2008[24] | Chronic low back pain | 8 | 8 | 51-70y; >71y | ↓GMV in middle corpus callosum, posterior parietal cortex and middle cingulate white matter volume |
| Ivo 2013[25] | Chronic low back pain | 14 | 14 | 51-70y | ↓total grey matter volume and ↓GMV in middle cingulate gyrus, thalamus and dorsolateral prefrontal cortex |
| Sacca 2024[26] | Chronic low back pain | Acute LBP = 115, Chronic LBP=243, total = 358 | 358 | 51-70y | No difference in whole brain white matter volumes, and ↓ whole brain grey matter volume in patient group |
| Luchtmann 2014[27] | Chronic low back pain due to lumbar disc herniation | 12 | 12 | 31-50y | ↓GMV in right anterolateral prefrontal cortex, temporal lobe, caudate nucleus and cerebellum; and left premotor cortex.  ↑GMV in right dorsal anterior cingulate cortex, left precuneal cortex, left fusiform gyrus and right brainstem |
| Nees 2020[28] | Chronic low back pain | 42 | 43 | 31-50y; 51-70y | ↓GMV in amygdala and nucleus accumbens, ↑GMV in hippocampus |
| Kong 2013[29] | Chronic low back pain | 18 | 18 | 31-50y | ↑cortical thickness in bilateral primary somatosensory cortices |
| Lamichhane 2021[30] | Chronic low back pain | 24 | 27 | 31-50y; 51-70y | ↑cortical thickness in the bilateral occipital, temporal and parietal lobes, posterior cingulate, temporal parietal junction and left motor and premotor cortices |
| Wei 2022[31] | Chronic sciatica | 34 | 36 | 51-70y | ↑cortical surface area in the superior temporal sulcus and rostral anterior cingulate |
| Zhang 2019[32] | Chronic low back pain | 90 (Cohort 1) and 18 (Cohort 2). | Healthy controls 74 (Cohort 1) and 18 (Cohort 2) | 31-50y | ↑GMV in left anterior cingulate cortex |
| Dolman 2014[33] | Chronic low back pain | 14 | 14 | 31-50y | No difference between groups after age was added as a covariate |
| Ung 2014[34] | Chronic low back pain | 47 | 47 | 31-50y | No difference in total brain volume but different distributions of grey matter density throughout brain between patients and controls |
| Li 2021[35] | Chronic low back pain due to lumbar disc herniation | 36 | 38 | 31-50y | No difference in GMV between groups |

LBP: Lower back pain, GMV: grey matter volume

## **Table S7: Chronic low back pain resting state fMRI results**

| Study | Number of pain group patients | Number of healthy controls | Age group(s) of patients | Main finding (pain group vs control) |
| --- | --- | --- | --- | --- |
| Baumbach 2022[36] | 35 | 36 | 31-50y; 51-70y | ↓activity in the salience network |
| Ng 2021[37] | 12 | 12 | 31-50y | ↓connectivity in the posterior cingulate cortex and angular gyrus |
| Tu 2019[38] | 50 | 44 | 31-50y | ↓connectivity between medial prefrontal cortex and default mode network |
| Chen 2023[39] | 56 | 51 | 31-50y | ↓ excitation between bilateral cerebellar crus to the paracentral lobule |
| Liu 2018[40] | 20 | 17 | >71y | ↓connectivity in anterior, middle and posterior cingulate cortex, inferior frontal, middle temporal |
| Zhu 2024[41] | 48 | 64 | 31-50y | ↑connectivity in the frontoparietal network, somatomotor network and thalamus |
| Shen 2019[42] | 90 | 74 | 31-50y | ↑connectivity between primary visual network and somatosensory/motor areas |
| Jiang 2016[43] | 17 | 17 | 31-50y | ↑connectivity between the amygdala and central executive network |
| Fan 2023[44] | 56 | 56 | 51-70y | ↑ALFF in precuneus and the default mode network |
| Lamichhane 2021[30] | 24 | 27 | 31-50y; 51-70y | No difference between groups |
| Zhang 2024[45] | 20 | 18 | 31-50y | ↑connectivity between the left lobule and left insular, orbitofrontal and bilateral medial prefrontal cortices; ↓connectivity between the right lobule or bilateral cerebellar crus I with the contralateral default mode, salience and emotional network |
| Tagliazucchi 2010[46] | 12 | 20 | 31-50y; 51-70y | Abnormal connections between default mode network and bilateral insular cortex and regions in the middle frontal gyrus |

ALFF: amplitude of low-frequency fluctuation

## **Table S8: Complex regional pain syndrome (CRPS) brain health results**

| Study | Pain type | Primary outcome measure | Number of pain group patients | Number of healthy controls | Age group(s) of patients | Main finding (pain group vs control) |
| --- | --- | --- | --- | --- | --- | --- |
| Hotta 2017[47] | CRPS | Fractional anisotropy; Mean diffusivity | 12 | 12 | 31-50y | ↓mean FA, ↑mean MD, ↔ AD |
| Im 2021[48] | CRPS | Fractional anisotropy; Mean diffusivity | 21 | 49 | 31-50y | ↓mean FA and AD in the prefrontal cortex. ↔ MD. |
| Pleger 2014[49] | CRPS type I | Grey matter volume (including cortical thickness) | 20 | 20 | 31-50y; 51-70y | ↑GMV in dorsomedial prefrontal cortex and primary motor cortex contralateral to affected limb |
| Barad 2014[50] | Right upper extremity CRPS | Grey matter volume (including cortical thickness) | 15 | 15 | 18-30y; 31-50y; 51-70y | ↓GMV in dorsal insula, left orbitofrontal and cingulate cortex, ↑GMV in bilateral dorsal putamen and right hypothalamus |
| Lee 2015[51] | CRPS | Grey matter volume (including cortical thickness) | 25 | 25 | 31-50y | ↓cortical thickness in right dorsolateral prefrontal cortex and left ventromedial prefrontal cortex |
| Domin 2021[52] | CRPS | Grey matter volume (including cortical thickness) | 24 | 33 | 51-70y | ↓GMV in bilateral thalamus |
| Shokouhi 2018[53] | CRPS | Grey matter volume (including cortical thickness); Cerebral blood flow in specific regions | 12 early stage CRPS (<10 months since diagnosis, haven't received sympathetic nerve block), 16 late stage CRPS = 28 | 16 | 51-70y | ↓GMV and perfusion in somatosensory cortex, and limbic system in early stage CRPS, ↔ GMV and ↑perfusion in late stage |
| Geha 2008[54] | CRPS | Grey matter volume (including cortical thickness); Fractional anisotropy | 26 | 28 | 31-50y | ↓GMV in right insula, ventromedial prefrontal cortex and nucleus accumbens, ↓FA in left cingulum callosal bundle |
| vanVelzen 2016[55] | CRPS | Grey matter volume (including cortical thickness); Resting state functional connectivity; Fractional anisotropy | 19 | 19 | 31-50y | ↔ in FA or total or regional GMV. rsfMRI altered connectivity in left posterior cingulate cortex to left executive attentional network |
| DiPietro 2020[56] | Upper limb CRPS | Resting state functional connectivity | 15 | 30 | 31-50y | ↑resting signal intensity in thalamus contralateral to the painful limb |
| Bolwerk 2013[57] |  | Resting state functional connectivity | 12 | 12 | 31-50y; 51-70y; >71y | ↑rsFC between primary somatosensory and motor cortex with cingulate, precuneus, thalamus, prefrontal cortex |
| Hong 2023[58] | CRPS | Resting state functional connectivity | 21 | 49 | 18-30y; 31-50y | ↑rsFC in bilateral somatosensory subnetworks, ↓ connectivity in prefronto-posterior cingulate, prefrontal, thalmo-anterior-cingulate networks |
| Kim 2017[59] | CRPS | Resting state functional connectivity | 25 | 25 | 31-50y | ↓rsFC between anterior and posterior insular cortices and the postcentral and inferior frontal gyri, cingulate cortices |
| Hok 2024[60] | CRPS | Resting state functional connectivity | 51 | 50 | 31-50y; 51-70y | ↔rsFC for predefined regions of interest between groups |
| Sano 2024[61] | Only higher than 20 in VAS | EEG | 17 with severe CRPS, 6 with reduced severity CRPS | 45 | 31-50y; 51-70y | Pain group exhibited distinct patterns in alpha wave envelope dynamics, reflecting excitatory and inhibitory activities |

CRPS: Complex regional pain syndrome; EEG: electroencephalography; FA: fractional anisotropy; MD: mean diffusivity; AD: axial diffusivity; rsFC: resting state functional connectivity; GMV: grey matter volume; rsfMRI: resting state functional magnetic resonance imaging

## **Table S9: Diabetic peripheral neuropathy results**

| Study | Pain type | Primary outcome measure | Number of pain group patients | Number of healthy controls | Age group(s) of patients | Main finding (pain group vs control) |
| --- | --- | --- | --- | --- | --- | --- |
| Cauda 2009[62] | Diabetic peripheral neuropathy | Resting state functional connectivity | 8 | 8 | 51-70y; >71y | ↓rsFC bilaterally in left fusiform, lingual and inferior temporal gyri, right inferior occipital gyrus, dorsal anterior cingulate cortex |
| Cauda 2009[63] | Diabetic peripheral neuropathy | Resting state functional connectivity | 8 | 8 | 31-50y; 51-70y; >71y | ↓rsFC thalamus and cortex |
| Cauda 2010[64] | Diabetic peripheral neuropathy | Resting state functional connectivity | 8 | 8 | 31-50y; 51-70y; >71y | ↓rsFC attentional networks, dorsal anterior cingulate cortex |
| Croosu 2023[65] | Diabetic peripheral neuropathy | Resting state functional connectivity | 19 | 20 | 31-50y; 51-70y | ↑rsFC in thalamus and motor areas between postcentral gyrus and motor areas |
| Chao 2022[66] | Diabetic peripheral neuropathy | Resting state functional connectivity | 24 | 27 healthy controls, 13 painless diabetic neuropathy | 31-50y; 51-70y; >71y | ↓rsFC in white matter in insula, hippocampus, parahippocampus, amygdala and middle temporal gyrus |

rsFC: resting state functional connectivity

## **Table S10: Temporomandibular disorder brain health findings**

| Study | Pain type | Primary outcome measure | Number of pain group patients | Number of healthy controls | Age group(s) of patients | Main finding (pain group vs control) |
| --- | --- | --- | --- | --- | --- | --- |
| Moayedi 2012[67] | Temporomandibular disorder | Fractional anisotropy | 17 | 17 | 31-50y | ↓FA in both trigeminal nerves, diffuse abnormalities in white matter microstructure in sensorimotor networks |
| Budd 2022[68] | Temporomandibular disorder | Fractional anisotropy; Mean diffusivity | 33 | 33 | 18-30y; 31-50y; 51-70y | ↓FA and ↑MD in right uncinate fasciculus and right cingulum |
| Gerstner 2011[69] | Temporomandibular disorder | Grey matter volume (including cortical thickness) | 9 | 9 | 18-30y | ↓GMV in left anterior and right posterior cingulate gyrus, right anterior insular cortex, left inferior frontal gyrus and superior temporal gyrus |
| Schmidt-Wilcke 2010[70] | Persistent idiopathic facial pain | Grey matter volume (including cortical thickness) | 11 | 11 | 51-70y | ↓GMV in left anterior cingulate gyrus and left temporoinsular region, bilateral sensorimotor areas |
| Wilcox 2015[71] | Temporomandibular disorder | Grey matter volume (including cortical thickness); Fractional anisotropy; Mean diffusivity | 22 | 40 | 31-50y | ↓GMV in medullary dorsal horn, ↑MD in dorsal horn, midbrain periaqueductal grey, and nucleus raphe magnus, ↓FA in trigeminal tracts |
| Gustin 2013[72] | Trigeminal neuropathy or temporomandibular disorder (TMD) | Grey matter volume (including cortical thickness) | 42 | 35 | 31-50y | Pain + depression correlated to ↓GMV in thalamus and cingulate, dorsolateral prefrontal and hippocampal cortices |
| Moayedi 2011[73] | Temporomandibular disorder | Grey matter volume (including cortical thickness) | 17 | 17 | 18-30y; 31-50y | ↑cortical thickness in thalamus |
| Gustin 2011[74] | Trigeminal neuropathy or temporomandibular disorder | Grey matter volume (including cortical thickness) | 21 TN, 20 TMD | 36 | 31-50y; 51-70y | ↔ No difference in GMV between TMD and control; but TN patients had significant ↓GMV in anterior insula, putamen, thalamus, and primary somatosensory cortex ipsilateral to ongoing pain and contralateral thalamus and nucleus accumbens, ↑GMV in contralateral insula |
| Younger 2010[75] | Temporomandibular disorder | Grey matter volume (including cortical thickness) | 15 | 15 | 31-50y | ↔No difference in overall grey matter volumes, ↑GMV in right inferior frontal gyrus, right anterior insula, posterior putamen, thalami, right globus pallidus and pons. ↓GMV in primary somatosensory cortex |
| Moayedi 2012[67] | Non traumatic temporomandibular dysfunction | Grey matter volume (including cortical thickness) | 17 | 17 | 31-50y | ↔ No difference in overall grey matter volumes. Patients had higher brain aging than controls |
| Domin 2021[76] | Temporomandibular disorder | Grey matter volume (including cortical thickness) | 47 in clinical, 57 in validation cohort | 60 in clinical, 381 in validation cohort | 18-30y; 31-50y | ↔ No difference in GMV between TMD and control |
| Lim 2021[77] | Temporomandibular disorder | Resting state functional connectivity | 12 | 24 | 18-30y | ↓BOLDSV in dorsolateral prefrontal cortex |
| Mills 2021[78] | Temporomandibular disorder | Resting state functional connectivity | 16 | 45 | 31-50y | ↑rsFC between rostral ventro-medial medulla and bilateral subnucleus reticularis dorsalis |
| Kucyi 2014[79] | Idiopathic temporomandibular disorder | Resting state functional connectivity | 17 | 17 | 31-50y | ↑rsFC between medial prefrontal cortex with other default mode network regions including the posterior cingulate cortex, precuneus and retrosplenial cortex |
| Chen 2022[80] | Temporomandibular disorder | Resting state functional connectivity | 21 | 30 | 18-30y | ↑ALFF in right parahippocampal gyrus, supplementary motor area, precentral gyrus and ↓ALFF in right cerebellum crus |
| Jasim 2020[81] | Temporomandibular disorder | Blood brain derived neurotrophic factor (BDNF) | 39 | 39 | 18-30y | Pain group had higher BDNF (263.33+/-345.13pg/ml) than controls (151.81+/-125.90) |

GMV: grey matter volume, TMD: temporomandibular disorder, FA: fractional anisotropy, MD: mean diffusivity, ALFF: amplitude of low frequency fluctuations, BOLDSV: Blood oxygenation functional magnetic resonance imaging response, BDNF: brain derived neurotrophic factor

## **Table S11: Trigeminal neuralgia brain health findings**

| Study | Pain type | Primary outcome measure | Number of pain group patients | Number of healthy controls | Age group(s) of patients | Main finding (pain group vs control) |
| --- | --- | --- | --- | --- | --- | --- |
| Zhou 2022[82] | Trigeminal neuralgia | Cerebral blood flow in specific regions | 28 | 30 | 31-50y; 51-70y | ↑cerebral blood flow in the thalamus, middle frontal gyrus and left insula |
| Li 2021[83] | Trigeminal neuralgia | Fractional anisotropy; Mean diffusivity | 22 | 22 | 51-70y | ↓FA↑MD in corpus callosum |
| Liu 2018[84] | Trigeminal neuralgia | Fractional anisotropy; Mean diffusivity | 29 | 35 | 31-50y; 51-70y; >71y | ↓FA in bilateral corona radiata, corpus callosum, cingulum frontal occipital fasciculus, internal capsule, external capsule and fornix cerebri, internal sagittal stratum and cerebral peduncle. ↔ MD |
| Li 2024[85] | Trigeminal neuralgia | Fractional anisotropy; Mean diffusivity | 32 | 32 | 51-70y | ↓FA↑MD in bilateral thalami |
| Xiong 2024[86] | Trigeminal neuralgia | Fractional anisotropy; Mean diffusivity | 36 | 36 | 31-50y; 51-70y | ↓FA↑MD in corona radiata, corpus callosum, internal capsule, superior longitudinal fasciculi, cingulum. |
| Filimonova 2024[87] | Trigeminal neuralgia | Fractional anisotropy; Mean diffusivity | 60 | 28 | 51-70y | ↓FA in corona radiata, internal capsule, optic radiation and thalami. ↔ MD |
| Desouza 2013[88] | Trigeminal neuralgia | Grey matter volume (including cortical thickness) | 24 | 24 | 31-50y | ↑GMV in thalamus, contralateral somatosensory cortex, amygdala, frontal pole, basal ganglia, periaqueductal grey, but cortical thinning in orbitofrontal cortex, insula and anterior cingulate cortex |
| Sun 2024[89] | Trigeminal neuralgia | Grey matter volume (including cortical thickness); Fractional anisotropy; Mean diffusivity | 46 | 35 | 51-70y | ↔GMV, ↓FA in corpus callosum, cerebral and cerebellar peduncles, thalamic radiation and internal capsule |
| Mammadkhanli 2024[90] | Trigeminal neuralgia | Grey matter volume (including cortical thickness) | 20 | 28 | 31-50y | ↓GMV in insula, posterior and middle cingulate cortex |
| Parise 2014[91] | Trigeminal neuralgia | Grey matter volume (including cortical thickness), Fractional anisotropy; Mean diffusivity | 24 | 24 | 31-50y; 51-70y | ↓cortical thickness in left cuneus and left fusiform cortex, ↔ FA/MD |
| Li 2017[92] | Trigeminal neuralgia | Grey matter volume (including cortical thickness) | 28 | 28 | 31-50y | ↓GMV in bilateral superior/middle temporal gyrus, bilateral parahippocampus, left anterior cingulate cortex, caudate, right fusiform gyrus, right cerebellum |
| Li 2021[93] | Trigeminal neuralgia | Grey matter volume (including cortical thickness) | 30 | 30 | 31-50y; 51-70y | ↓cortical thickness in left inferior frontal and medial orbitofrontal cortex |
| Albano 2022[94] | Trigeminal neuralgia with previous Gamma Knife Surgery | Grey matter volume (including cortical thickness) | 30 | 15 | 51-70y | ↓cortical thickness of temporal, prefrontal, cingulate, somatosensory and occipital areas |
| Wang 2019[95] | Trigeminal neuralgia | Grey matter volume (including cortical thickness) | 40 | 40 |  | ↓GMV in insula, secondary somatosensory cortex, hippocampus, dorsal anterior cingulate cortex, precuneus |
| Obermann 2013[96] | Trigeminal neuralgia | Grey matter volume (including cortical thickness) | 60 | 49 | 51-70y | ↓GMV in primary somatosensory and orbitofrontal cortices, thalamus, secondary somatosensory cortex, insula, anterior cingulate cortex, cerebellum and dorsolateral prefrontal cortex |
| Ge 2023[97] | Trigeminal neuralgia | Grey matter volume (including cortical thickness) | 79 | 83 | 51-70y | ↓GMV in right superior temporal pole and precentral gyrus |
| Wilcox 2015[98] | Trigeminal neuralgia | Grey matter volume (including cortical thickness); Fractional anisotropy | 22 | 44 | 31-50y | ↓GMV ↓MD and ↑FA in spinal trigeminal nucleus |
| Hayes 2017[99] | Trigeminal neuralgia | Grey matter volume (including cortical thickness); Fractional anisotropy; Mean diffusivity | 37 | 28 | 31-50y; 51-70y | ↓GMV and FA, ↑MD and RD in middle and posterior cingula, bilateral hippocampi and nucleus accumbens |
| Latypov 2023[100] | Classical trigeminal neuralgia, trigeminal neuropathic pain | Grey matter volume (including cortical thickness); Fractional anisotropy; Mean diffusivity | 265 classical TN, 106 trigeminal neuropathic pain | 108 | 51-70y | ↓GMV in temporal lobes, insulae, olfactory cortex and parahippocampal gyrus |
| Zhang 2018[101] | Trigeminal neuralgia | Grey matter volume (including cortical thickness); Resting state functional connectivity | 29 | 34 | 31-50y | ↓GMV in bilateral amygdala, periaqueductal grey and right insula. Significantly altered FC in amygdala to putamen/prefrontal cortices |
| Wang 2018[102] | Trigeminal neuralgia | Grey matter volume (including cortical thickness); Resting state functional connectivity; Fractional anisotropy; Mean diffusivity | 20 | 21 | 51-70y | ↓cortical thickness in left insular cortex and ↑FC between insula to left posterior cingulate cortex and thalamus |
| Wang 2017[103] | Trigeminal neuralgia | Grey matter volume (including cortical thickness); Resting state functional connectivity; Fractional anisotropy; Mean diffusivity | 38 | 38 | 31-50y; 51-70y | ↓GMV in anterior and mid cingulate cortex, insula, primary motor cortex, secondary somatosensory cortex and temporal lobe; ↓FA↑MD in corpus callosum and corona raidata, ↑FC between right insula and anterior and posterior cingulate and medial and dorsal prefrontal cortex |
| Wu 2020[104] | Trigeminal neuralgia | Grey matter volume (including cortical thickness); White matter hyperintensities | 23 with neurovascular compression, 22 without | 45 | 31-50y; 51-70y | ↓GMV in different regions for both groups, including the anterior cingulate cortex and thalamus |
| Wang 2015[105] | Trigeminal neuralgia | Resting state functional connectivity | 17 | 19 | 51-70y | ↓ReHo in left amygdala, right parahippocampal gyrus and left cerebellum; ↑ReHo in right inferior temporal gyrus, thalamus, inferior parietal lobule |
| Yuan 2018[106] | Trigeminal neuralgia | Resting state functional connectivity | 23 | 23 | 51-70y | Abnormal ReHo and ALFF in cerebellum, cingulate cortex, temporal lobe, putamen, occipital lobe, limbic lobe, precuneus, insula, medial and superior frontal gyrus |
| Tsai 2019[107] | Trigeminal neuralgia | Resting state functional connectivity | 25 | 20 | 51-70y | Abnormal participation coefficient of sensorimotor network and default mode network |
| Zhang 2019[108] | Trigeminal neuralgia | Resting state functional connectivity | 29 | 34 | 31-50y | ↓ALFF in left middle/superior temporal gyrus, bilateral medial prefrontal cortex, anterior cingulate cortex, bilateral postcentral gyrus and right middle frontal gyrus |
| Zhang 2021[109] | Trigeminal neuralgia | Resting state functional connectivity | 41 | 43 | 51-70y | Abnormal connections within the default mode network |
| Xu 2022[110] | Trigeminal neuralgia | Resting state functional connectivity | 48 | 46 | 51-70y | ↓FC in bilateral posterior cingulate cortex, right angular gyrus, bilateral precentral and postcentral gyrus and bilateral anterior cingulate cortex and left anterior insula |

FC: Functional connectivity; ALFF: amplitude of low frequency fluctuations; ReHo: regional homogeneity; rsfMRI: resting state functional magnetic resonance imaging; GMV: grey matter volumes; FA: fractional anisotropy; MD: mean diffusivity

## **Table S12: Fibromyalgia structural and diffusion MRI findings**

| Study | Primary outcome measure | Number of pain group patients | Number of healthy controls | Age group(s) of patients | Main finding (pain group vs control) |
| --- | --- | --- | --- | --- | --- |
| Puri 2010[111] | Grey matter volume (including cortical thickness) | 5 | 5 | 31-50y | ↓GMV in left supplementary motor area and right superior frontal gyrus |
| Feraco 2020[112] | Grey matter volume (including cortical thickness) | 12 | 12 | 31-50y | ↓GMV in left anterior cingulate gyrus, right mid-posterior cingulate gyrus and right inferior occipital gyrus |
| Schmidt-Wilcke 2007[113] | Grey matter volume (including cortical thickness) | 20 | 22 | 51-70y | ↓GMV in right superior temporal gyrus, left posterior thalamus, ↑GMV in left orbitofrontal cortex, cerebellum, striatum |
| Leon-Llamas 2021[114] | Grey matter volume (including cortical thickness) | 49 | 43 | 51-70y | ↓GMV in hippocampus |
| Niddam 2017[115] | Grey matter volume (including cortical thickness) | 21 | 21 | 31-50y | ↓GMV in bilateral superior frontal gyri, right ventrolateral and dorsolateral prefrontal cortex, bilateral claustra, anterior insula, bilateral middle and inferior temporal gyri. |
| Mosch 2023[116] | Grey matter volume (including cortical thickness) | 23 | 21 | 51-70y | ↓GMV in bilateral middle temporal gyrus, parahippocampal gyrus, left dorsal anterior cingulate cortex, right putamen, right caudate nucleus, left dorsolateral prefrontal cortex. ↑GMV in bilateral cerebellum and left thalamus |
| Lutz 2008[117] | Grey matter volume (including cortical thickness); Fractional anisotropy; Mean diffusivity | 30 | 30 | 51-70y | ↓GMV, ↑FA in postcentral gyri, amygdalae, hippocampi, superior frontal gyri and anterior cingulate gyri. ↓FA bilateral thalami, thalamo-cortical tracts and insulae |
| Ceko 2013[118] | Grey matter volume (including cortical thickness) | 28 | 28 | 31-50y; 51-70y | ↓GMV in older patients in posterior cingulate cortex, ↑GMV in younger patients in insula and basal ganglia |
| Hsu 2009[119] | Grey matter volume (including cortical thickness) | 29 | 29 | 31-50y | ↔No difference in total GMV, smaller left anterior insula in fibromyalgia with affective disorders vs controls |
| OliveriaNeto 2024[120] | Grey matter volume (including cortical thickness) | 33 | 33 | 31-50y | ↑cortical thickness in precentral gyrus, postcentral gyrus, superior parietal gyrus, entorhinal, inferior parietal gyrus. ↓cortical thickness in insula. |
| Diaz-Piedra 2016[121] | Grey matter volume (including cortical thickness); Total brain volume | 24 | 24 | 18-30y; 31-50y | ↑GMV in superior frontal gyrus and cerebellum, ↓total GMV |
| Nhu 2023[122] | Resting state functional connectivity, grey matter volume | 26 | 30 | 31-50y; 51-70y | ↑FC in visual networks, ↑GMV in lingual gyrus, lateral occipital cortex and pericalcarine cortex |
| Izuno 2023[123] | Grey matter volume (including cortical thickness); Total brain volume | 34 | 25 | 31-50y | ↑White matter volume of temporal pole |
| Sundgren 2007[124] | Fractional anisotropy | 19 | 25 | 31-50y | ↔No difference FA |
| Tu 2022[125] | Fractional anisotropy, mean, radial and axial diffusivity | 20 | 20 | 31-50y | ↑FA and ↓MD,RD, AD in multiple tracts in corpus callosum |

FA: fractional anisotropy, MD: mean diffusivity, RD: radial diffusivity, AD: axial diffusivity, GMV: grey matter volume

## **Table S13: Fibromyalgia resting state functional MRI findings**

| Study | Primary outcome measure | Number of pain group patients | Number of healthy controls | Age group(s) of patients | Main finding (pain group vs control) |
| --- | --- | --- | --- | --- | --- |
| Cifre 2012[126] | Resting state functional connectivity | 9 | 11 | 31-50y | ↑FC between anterior cingulate cortex (ACC) and insula, basal ganglia; secondary somatosensory area and caudate, primary motor cortex with supplementary motor area, globus pallidus and amygdala and superior temporal sulcus, and medial prefrontal cortex with posterior cingulate cortex (PCC) and caudate. ↓FC between ACC and amygdala and periaqueductal grey (PAG), thalamus to insula and PAG, insula and putamen and PAG with caudate, secondary somatosensory area with motor cortex and PCC and PCC with superior temporal sulcus |
| Coulombe 2017[127] | Resting state functional connectivity | 23 | 16 | 31-50y | ↑FC in periaqueductal grey to lingual gyrus and hippocampus, ↓FC to regions associated with motor and executive functions including the default mode network |
| Napadow 2010[128] | Resting state functional connectivity | 18 | 18 | 31-50y | ↑FC within the default mode network and executive attention network and insula |
| Ichesco 2014[129] | Resting state functional connectivity | 18 | 18 | 31-50y | ↑FC between right mid insula and right mid/posterior cingulate cortex and right mid insula, left corpus callosum, left superior temporal gyrus |
| Kim 2013[130] | Resting state functional connectivity | 19 | 20 | 31-50y | ↑FC primary somatosensory cortex, supplementary motor area, dorsolateral prefrontal cortex, amygdala |
| Truini 2016[131] | Resting state functional connectivity | 20 | 15 | 18-30y; 31-50y; 51-70y | ↑FC between periaqueductal grey with anterior cingulate cortex, amygdala and insula |
| Nhu 2023[122] | Resting state functional connectivity, grey matter volume | 26 | 30 | 31-50y; 51-70y | ↑FC in visual networks, ↑GMV in lingual gyrus, lateral occipital cortex and pericalcarine cortex |
| Kaplan 2019[132] | Resting state functional connectivity | 40 | 46 | 31-50y | ↑FC in areas such as operculum insula, superior temporal gyrus and posterior cingulate cortex |
| Park 2022[133] | Resting state functional connectivity | 32 | 37 | 31-50y; 51-70y | ↓FC between right nucleus accumbens and left putamen, thalamus and ventral pallidum |
| Larkin 2021[134] | Resting state functional connectivity | 77 | 41 | 18-30y; 31-50y; 51-70y; >71y | Variable network organisation in patients compared to controls in posterior and anterior cingulate cortices, salience network and angular gyrus |

FC: functional connectivity, GMV: grey matter volume

**Table S14: Fibromyalgia resting state electroencephalography (EEG) study findings**

| Study | Primary outcome measure | Number of pain group patients | Number of healthy controls | Age group(s) of patients | Main finding (pain group vs control) |
| --- | --- | --- | --- | --- | --- |
| Alves 2023[135] | EEG | 49 | 15 | 18-30y; 31-50y; 51-70y | ↑connectivity between different pain processing circuits particularly in beta-3 frequency band |
| Gonzalez-Villar 2020[136] | EEG | 43 | 51 | 31-50y | ↑beta connectivity between different brain networks |
| Fallon 2018[137] | EEG | 19 | 18 | 31-50y | ↑theta activity in prefrontal and anterior cingulate cortices |
| Makowka 2023[138] | EEG | 16 | 11 | 51-70y | ↓functional connectivity in the high alpha sub-band than controls in the left basolateral complex of the amygdala |
| Gonzalez-Roldan 2016[139] | EEG | 20 | 18 | 51-70y | ↓power density of delta EEG band over right insula, superior and middle temporal gyri |
| Villafaina 2019[140] | EEG | 31 | 31 | 51-70y | ↓alpha-2 EEG power in C4, T3, P4, Pz and O2 |
| Martin-Brufau 2021[141] | EEG | 23 | 23 | 51-70y | Mixed altered activity in all EEG bands except delta |
| Vanneste 2017[142] | EEG | 44 | 44 | 31-50y | ↓alpha-1 EEG activity in posterior cingulate cortex extending to precuneus, ↑beta 1, 2 and 3 activity in posterior cingulate cortex to precuneus and dorsal anterior cingulate cortex and subgenual anterior cingulate cortex |

EEG: Electroencephalography

## **Table S15: Headache structural and diffusion MRI study findings**

| Study | Pain category | Primary outcome measure | Number of pain group patients | Number of healthy controls | Age group(s) of patients | Main finding (pain group vs control) |
| --- | --- | --- | --- | --- | --- | --- |
| Gomez-Beldarrain 2015[143] | Migraine | Fractional anisotropy | 19 episodic migraine, 18 chronic migraine | 15 | 31-50y | ↓FA in right anterior insula, cingulate gyrus, uncinate fasciculus |
| TantikPak 2023[144] | Migraine | Fractional anisotropy; Mean diffusivity | 51 | 44 | 31-50y | ↓FA in genu of corpus callosum |
| Coppola 2020[145] | Migraine | Fractional anisotropy; Mean diffusivity | chronic migraines (18), episodic migraine without aura (19) | 18 | 18-30y; 31-50y | ↑MD in right superior and posterior corona radiata, right superior longitudinal fasciculus, right splenium of corpus callosum |
| Naegel 2014[146] | Cluster headache | Grey matter volume (including cortical thickness) | 91 total: 46 episodic out of bout, 22 episodic in bout, 23 chronic | 78 | 31-50y | ↓GMV in ipsilateral temporal lobe and contralateral dorsal hippocampus, ↑GMV in bilateral cerebellum and ipsilateral posterior insula |
| Hubbard 2014[147] | Migraine | Grey matter volume (including cortical thickness); Resting state functional connectivity | 17 | 18 | 31-50y | ↑GMV and ↓cortical thickness in left anterior midcingulate |
| Schmidt-Wilcke 2008[148] | Migraine | Grey matter volume (including cortical thickness) | 35 | 31 | 18-30y; 31-50y | ↓GMV in anterior and posterior cingulate cortex and right insula |
| Mammadkhanli 2024[149] | Migraine | Grey matter volume (including cortical thickness) | 21 migraine without aura, 17 with aura | 28 | 31-50y | ↓GMV in smaller posterior insula, parietal operculum and insular cortex |
| Schmidt-Wilcke 2005[150] | Other (mixed) | Grey matter volume (including cortical thickness) | 20 Chronic tension type headache and 20 medication overuse headache | 40 | 18-30y; 31-50y; 51-70y | ↓GMV in dorsal, rostral and ventral pons, anterior cingulate cortex, insulae, right posterior temporal lobe, orbitofrontal cortex, parahippocampus and right cerebellum in tension headache but no difference in medication overuse headache |
| Neeb 2017[151] | Migraine | Grey matter volume (including cortical thickness) | 21 chronic migraine, 21 episodic migraine = 42 | 21 | 31-50y | ↑GMV in amygdala and putamen |
| Ferraro 2022[152] | Cluster headache | Grey matter volume (including cortical thickness); Resting state functional connectivity | 28 | 28 | 31-50y | ↑GMV mesocorticolimbic volumes, ↓rsFC in right frontal pole – right amygdala pathway *Note: study was on patients hospitalised in acute pain |
| Chen 2016[153] | Other (mixed) | Grey matter volume (including cortical thickness) | 40 | 40 | 31-50y | ↔No difference between groups |
| DeRidder 2024[154] | Migraine | White matter hyperintensities | 61 episodic migraine, 31 chronic migraine, 36 migraine + aura, 56 migraine without aura | 24 | 31-50y | Prevalence of WMH similar across migraine groups but control group showed no WMH at all. |

GMV: Grey matter volume, WMH: white matter hyperintensities, FA: fractional anisotropy, MD: mean diffusivity, rsFC: resting state functional connectivity

## **Table S16: Headache resting-state functional MRI study findings**

| Study | Pain type | Number of pain group patients | Number of healthy controls | Age group(s) of patients | Main finding (pain group vs control) |
| --- | --- | --- | --- | --- | --- |
| Ferraro 2018[155] | Cluster headache | 17 | 16 | 31-50y | ↑FC between ipsilateral hypothalamus and ventral tegmental area, dorsal nuclei of raphe, bilateral substantia nigra, subthalamic nucleus, red nucleus, ipsilateral lingual gyrus, contralateral superior and middle temporal gyrus |
| Liu 2023[156] | Migraine | 28 | 17 | 31-50y | ↑FC between left periaqueductal grey and left anterior cingulate gyrus and right postcentral gyrus; right periaqueductal grey to left precentral gyrus and left caudate and right middle occipital gyrus to right periaqueductal grey |
| Zhang 2020[157] | Migraine | 30 | 22 | 31-50y | ↑FC left lateral geniculate nucleus in left cerebellum and right lingual gyrus. No difference in volumes. |
| Gecse 2022[158] | Migraine | 34 | 48 | 18-30y | ↑FC in periaqueductal grey and nucleus cuneiformis |
| Liu 2015[159] | Migraine | 108 | 30 | 18-30y | ↑FC in migraine across a wide range of networks |
| Zhang 2023[160] | Migraine without aura | 7 acute, 27 interictal | 29 | 31-50y | ↑ALFF in trigeminal-cervical cortex in acute migraine compared to interictal and healthy controls |
| Huang 2019[161] | Migraine | 30 | 22 | 31-50y | ↓FC between left red nucleus and middle frontal gyrus; right nucleus to ipsilateral superior parietal lobe and ↑FC ipsilateral cerebellum |
| Schwedt 2014[162] | Migraine | 38 | 20 | 31-50y | Altered FC in sensorimotor areas and visual cortex, temporal cortex, posterior parietal lobule, prefrontal areas, precuneus, cingulate gyrus, and cerebellum |
| Qin 2020[163] | Migraine | 48 | 48 | 31-50y | Altered FC in bilateral hippocampus, bilateral insula, right amygdala, right anterior cingulate cortex, bilateral putamen, bilateral caudate nucleus, prefrontal cortex |
| Gao 2016[164] | Migraine | 55 | 44 | 31-50y | Altered FC in bilateral hippocampus, insula, putamen, caudate, prefrontal cortex, right amygdala and right anterior cingulate cortex |
| Maki-Marttunen 2023[165] | Migraine | 99 chronic migraine, 22 chronic back pain, 25 episodic migraine | 27 healthy controls, 17 clinical depression | 31-50y | Altered FC in a number of resting state networks, mostly the visual cortex |
| Li 2016[166] | Migraine | 100 | 46 | 18-30y | ↓FC between periaqueductal grey and rostral anterior cingulate cortex and medial frontal cortex |
| Chen 2016[167] | Episodic migraine, chronic migraine and medication overuse headache | 18 episodic migraine, 16 chronic, 44 medication overuse | 32 | 31-50y | ↓FC in marginal division of neostriatum in episodic and chronic migraine groups |

FC: Functional connectivity, ALFF: amplitude of low frequency fluctuations

## **Table S17: Hip and knee pain MRI study findings**

| Study | Pain type | Primary outcome measure | Number of pain group patients | Number of healthy controls | Age group(s) of patients | Main finding (pain group vs control) |
| --- | --- | --- | --- | --- | --- | --- |
| Feng 2020[168] | Osteonecrosis of the femoral head | Resting state functional connectivity | 10 | 10 | 51-70y | ↑ALFF in right middle occipital gyrus, inferior parietal lobule, insula, superior temporal and lingual gyrus and parahippocampal gyrus and left angular gyrus, precuneus, median cingulate and paracingulate gyrus |
| Barroso 2020[169] | Advanced hip and knee osteoarthritis | Grey matter volume (including cortical thickness) | 115 | 36 | 51-70y | ↓GMV in anterior cingulate and paracingulate cortex in hip pain; ↓GMV in primary motor cortex in hip and knee pain |
| Gwilym 2010[170] | Right sided hip pain | Grey matter volume (including cortical thickness) | 16 | 16 | 51-70y | ↓GMV in thalamus in hip pain pre surgery, no difference in total GMV |
| Johnson 2022[171] | Knee pain | Brain age gap calculations | 94 | 19 | 51-70y | ↓brain age in patients with pain who used non-pharmacological methods to control pain compared to patients with chronic pain and controls |
| Johnson 2022[172] | Knee pain | Brain age gap calculations | 111 low impact, 60 high impact | 31 | 51-70y | ↑brain age difference in high impact chronic knee pain compared to individuals with low impact knee pain |
| Valdes-Hernandez 2023[173] | Knee pain and chronic lower back pain | Brain age gap calculations | 169 OA, 170 CBP = 339 | 321 | 31-50y; 51-70y | ↑Brain age difference in osteoarthritis compared to back pain, or osteoarthritis and chronic back pain compared to controls; no difference between chronic back pain compared to controls |
| Iwabuchi 2020[174] | Knee pain | Cerebral blood flow in specific regions | 44 | 29 | 51-70y | ↓perfusion in anterior default mode and salience network hubs, ↑perfusion in posterior default mode, thalamus and sensory regions |
| Cottam 2016[175] | Knee pain; Osteoarthritis | Cerebral blood flow in specific regions | 26 | 27 | 51-70y | ↑blood flow in anterior mid-cingulate cortex, subgenual cingulate cortex, bilateral hippocampi and amygdala, left central operculum, mid-insula, putamen and brainstem |
| Sanchis-Alfonso 2023[176] | Knee pain | Grey matter volume (including cortical thickness); Resting state functional connectivity | 24 | 21 | 18-30y | ↓FC in left cuneus and right anterior insular cortex and ↑FC in rostral prefrontal cortex and right supplementary motor area |
| Iwabuchi 2023[177] | Knee pain | Resting state functional connectivity | 74 | 36 |  | ↓FC in subgenual anterior cingulate, ventromedial prefrontal cortex, insula, hippocampus, cerebellum, supramarginal gyrus, middle and inferior temporal gyrus, ↑FC in cuneus, cerebellum precentral gyrus, anterior cingulate cortex, inferior temporal gyrus, postcentral and superior frontal gyrus, lateral occipital cortex and putamen |
| Mao 2016[178] | Knee pain; Osteoarthritis | Grey matter volume (including cortical thickness) | 26 | 31 | 51-70y | ↓GMV bilateral caudate nucleus and hippocampus in knee osteoarthritis |
| Liao 2018[179] | Knee pain; Osteoarthritis | Grey matter volume (including cortical thickness) | 30 | 30 | 51-70y | ↓total GMV and bilateral orbital frontal cortex, ↓GMV in right lateral prefrontal cortex and precentral and postcentral cortices |
| Zeng 2024[180] | Knee pain; Osteoarthritis | Grey matter volume (including cortical thickness) | 36 | 25 | 31-50y | ↓GMV in amygdala |
| Kang 2022[181] | Knee pain; Osteoarthritis | Grey matter volume (including cortical thickness); Resting state functional connectivity | 37 | 37 | 51-70y; >71y | ↓GMV in left middle temporal gyrus and left inferior temporal gyrus; ↓FC between middle temporal gyrus to dorsolateral superior frontal, left middle frontal and left medial superior frontal gyrus |
| Ushio 2024[182] | Knee pain; Osteoarthritis | Resting state functional connectivity | 15 | 15 | >71y | ↑FC between left anterior insular cortex to right orbitofrontal cortex, bilateral frontal pole, subcallosal area, medial frontal cortex; and right anterior insular cortex to bilateral orbitofrontal cortex, frontal pole, subcallosal area, medial frontal cortex |

GMV: grey matter volume, FC: functional connectivity, ALFF: amplitude of low frequency fluctuations

## **Table S18: Chronic musculoskeletal pain study findings**

| Study | Pain type | Primary outcome measure | Number of pain group patients | Number of healthy controls | Age group(s) of patients | Main finding (pain group vs control) |
| --- | --- | --- | --- | --- | --- | --- |
| Lieberman 2014[183] | Musculoskeletal pain | Fractional anisotropy | 46 | 33 | 31-50y | ↓FA in splenium of corpus callosum and left cingulum |
| VanRiper 2017[184] | Musculoskeletal pain in Persian gulf war veterans | Fractional anisotropy; Mean diffusivity | 30 | 31 | 31-50y | ↓white matter integrity across various pain brain regions including corpus callosum and corona radiata |
| Ninneman 2022[185] | Musculoskeletal pain in Persian gulf war veterans | Grey matter volume (including cortical thickness) | 108 | 62 | 31-50y | ↓GMV in bilateral insula and ↑GMV in frontal pole |
| Bishop 2018[186] | Musculoskeletal pain | Grey matter volume (including cortical thickness); Fractional anisotropy; Mean diffusivity | 74 | 31 | 31-50y | ↑GMV in caudate, amygdala and hippocampus, ↓GMV in left dorsolateral prefrontal cortex and primary somatosensory areas, ↓white matter density in dorsal attention network |
| DukeHan 2013[187] | Musculoskeletal pain | Resting state functional connectivity | 64 | 64 | >71y | ↑FC between posterior cingulate and left insula, superior temporal gyrus and left cerebellum |
| Conboy 2021[188] | Rotator cuff tear | Grey matter volume (including cortical thickness); Cerebral blood flow in specific regions; Resting state functional connectivity | 21 (imaging), 13 (behaviour) | 18 (imaging), 11 (behaviour) | >71y | ↓GMV in primary somatosensory cortex, anterior intraparietal sulcus, inferior parietal lobule and temporo-parietal junction; altered motor network connectivity with middle temporal visual cortex, ↓FA in frontal occipital fasciculus and inferior longitudinal fasciculus |
| Chatterjee 2023[189] | Osteoarthritis | Resting state functional connectivity | 51 | 20 | 51-70y | ↓FC in frontal, parietal lobe, middle frontal and superior temporal gyri |
| Song 2021[190] | Chronic myofascial pain | Cerebral blood flow in specific regions; Resting state functional connectivity | 23 | 23 | 18-30y; 31-50y | ↓whole brain grey matter cerebral blood flow and functional connectivity coupling, and in bilateral superior temporal gyri, right parahippocampal gyrus, right hippocampus, caudate, right medial prefrontal cortex, periaqueductal grey; ↑FC in bilateral lingual gyri, posterior cingulate and bilateral inferior parietal lobules |
| Wang 2023[191] | Ankle pain | Resting state functional connectivity | 28 UKB 15 clinical | 109 UKB 15 clinical | 51-70y | Altered FC in cingulate motor area and insula |
| Meneses 2016[192] | Rheumatoid arthritis | EEG | 21 | 21 | 31-50y | ↑absolute alpha power density in all brain regions |
| Niddam 2019[193] | Shoulder pain | Grey matter volume (including cortical thickness) | 22 | 22 | 31-50y | ↓GMV in right central sulcus, posterior insula, inferior frontal and dorsomedial prefrontal cortices, precuneus, middle temporal and left medial orbitofrontal cortex |
| Li 2024[194] | Adhesive capsulitis | Grey matter volume (including cortical thickness); Resting state functional connectivity | 52 | 52 | 31-50y | ↓ReHo and ↓GMV within right medial prefrontal cortex |
| Li 2024[195] | Frozen shoulder | Grey matter volume (including cortical thickness); Resting state functional connectivity | 54 | 52 | 31-50y | ↓GMV in bilateral thalami and ↑ALFF in left thalamus and bilateral anterior cingulate cortex |
| Wei 2022[196] | Shoulder pain | Grey matter volume (including cortical thickness); Resting state functional connectivity | 22 left 15 right = 37 | 24 | 51-70y | Altered functional connectivity in caudate, paracentral lobule and precentral region and asymmetrical surface area indices |
| Li 2020[197] | Shoulder pain | Resting state functional connectivity | 37 | 24 | 51-70y | ↑ReHo values in left middle temporal gyrus and ↓ReHo in right orbitofrontal cortex |
| Russell 2018[198] | Hand pain; Osteoarthritis | Grey matter volume (including cortical thickness) | 28 | 11 | 51-70y | ↓GMV in anterior cingulate cortex that persisted after treatment with centrally acting analgesics pregabalin and duloxetine |

GMV: grey matter volume; ReHo: Regional homogeneity; ALFF: amplitude of low frequency fluctuations; EEG: electroencephalography; FA: fractional anisotropy; FC: functional connectivity; UKB: UK Biobank

## **Table S19: Mixed pain magnetic resonance imaging study findings**

| Study | Pain type | Primary outcome measure | Number of pain group patients | Number of healthy controls | Age group(s) of patients | Main finding (pain group vs control) |
| --- | --- | --- | --- | --- | --- | --- |
| Hung 2022[199] | Chronic back pain (BP); Osteoarthritis (OA); Trigeminal neuralgia (TN) | Brain age gap calculations | 147 (45 TN, 52 OA, 50 BP) | 812 | 31-50y; 51-70y | ↑brain age in trigeminal neuralgia and osteoarthritis, but not back pain |
| Cruz-Almeida 2019[200] | Neck and shoulder 12.1%, back 21.2%, wrist 12.1%, knees 30.3%, hip 6.1% | Brain age gap calculations | 33 | 14 | 51-70y; >71y | ↑brain age difference in pain group by +1.5 years |
| Malinen 2010[201] | Mixed: complex regional pain syndrome, posttraumatic arthiritis, lumbar spine degeneration, phantom limb pain, post-surgical, cervical spine degeneration | Resting state functional connectivity | 10 | 10 | 51-70y | Altered FC in lower insula and anterior cingulate cortex |
| Huang 2016[202] | Irritable bowel syndrome (IBS); urological chronic pelvic pain (UCPPS) | Fractional anisotropy; Mean diffusivity | 91 (52 UCPPS, 39 IBS) | 61 | 31-50y | Altered FA clusters particularly in right corticospinal tract and right anterior thalamic radiation |
| Woodworth 2015[203] | Irritable bowel syndrome (IBS); urological chronic pelvic pain (UCPPS) | Fractional anisotropy; Mean diffusivity | 84 (45 UCPPS, 39 IBS) | 56 | 31-50y | ↓FA, ↑MD in brain regions such as putamen |
| Gupta 2018[204] | Irritable bowel syndrome (IBS); Pelvic pain; Vulvodynia | Fractional anisotropy; Mean diffusivity | 58 (29 PVD, 29 IBS) | 29 | 31-50y | ↑FA in somatosensory and basal ganglia in PVD compared to HCs. No differences between PVD and IBS |
| Bell 2024[205] | Probable chronic pain from questionnaires | Grey matter volume (including cortical thickness) | 60 | 364 | 51-70y | ↓GMV in hippocampus, lower rostral middle caudate |
| Baliki 2011[206] | Chronic back pain; Complex regional pain syndrome (CRPS); Osteoarthritis | Grey matter volume (including cortical thickness) | 84 | 46 | 18-30y; 31-50y; 51-70y | ↓whole brain GMV in chronic back pain. Different chronic pain types have different brain signatures |
| Ruscheweyh 2011[207] | Chronic back pain; Migraine; Knee pain | Grey matter volume (including cortical thickness) | 45 | 31 | 51-70y | ↓GMV in cingulate, prefrontal and motor/premotor regions. No difference in pain that had stopped for > 12 months |
| Sundermann 2019[208] | Fibromyalgia (FM); Osteoarthritis (OA) | Grey matter volume (including cortical thickness) | 48 (25 FM, 23 OA) | 21 | 31-50y; 51-70y | ↔No differences in local GMV between 3 groups |
| Ezzati 2019[209] | Moderate or severe chronic pain in at least 1 location | Grey matter volume (including cortical thickness) | 72 | 59 | >71y | ↓GMV in right hippocampus |
| Bhatt 2024[210] | Headache, neck and shoulder, back, knee, hip, multisite | Grey matter volume (including cortical thickness) | 10,984 | 10,984 | 51-70y | ↓GMV throughout cortex particularly chronic multisite pain; abdominal pain had lower subcortical volumes and lower cerebellum volumes. Headache had thicker cortex compared to controls |
| Neumann 2023[211] | Chronic back pain, migraine, craniomandibular disorder | Grey matter volume (including cortical thickness) | 305 (174 back pain, 92 migraine, 39 craniomandibular disorder) | 296 | 31-50y; 51-70y | ↓GMV in left anterior and posterior insula, anterior cingulate cortex, left hippocampus |
| Yang 2017[212] | Chronic back pain; Cluster headache | Grey matter volume (including cortical thickness); Resting state functional connectivity | 88 (39 headache, 49 lower back pain) | 88 |  | Altered cortical thickness and GMV in left posterior cingulate cortex and rostral middle frontal gyrus and other subcortical areas in headache; ↑FC in headache compared to controls |
| Ikeda 2018[213] | Fibromyalgia; Chronic back pain; Complex regional pain syndrome (CRPS); Neuropathic pain; Phantom limb pain | Grey matter volume (including cortical thickness); Resting state functional connectivity | 23 | 17 | 31-50y | ↓GMV in right anterior insular cortex and left middle cingulate cortex |
| Kutch 2017[214] | Fibromyalgia (FM); urologic chronic pelvic pain syndrome (UCPSS) | Grey matter volume (including cortical thickness); Resting state functional connectivity | 315 (73 UCPSS neuroimaging sample, 219 UCPSS non-neuroimaging sample, 23 FM) | 49 | 31-50y | ↑GMV and FC in sensorimotor and insular cortices |
| McConnell 2020[215] | Prescription opioid users with chronic low back, neck, knee pain | Grey matter volume (including cortical thickness); Resting state functional connectivity | 31 | 30 | 51-70y | ↓GMV and FC in left nucleus accumbens and ventromedial prefrontal cortex in opioid group relative to controls |
| Lam 2024[216] | Fibromyalgia (FM); temporomandibular disorder (TMD) | Grey matter volume (including cortical thickness); White matter hyperintensities; Fractional anisotropy; Mean diffusivity | 33 (16 FM, 17 TMD) | 10 | 31-50y | ↓GMV in right thalamus in FM compared to TMD; ↓cortical thickness in right anterior prefrontal cortex than controls |
| Davis 2016[217] | Chronic back pain; Osteoarthritis | Resting state functional connectivity | 84 (40 back pain and 44 osteoarthritis) | 88 | 31-50y; 51-70y | Altered FC in thalamus in pain group |
| Pujol 2022[218] | Fibromyalgia (FM); Knee osteoarthritis (OA) | Resting state functional connectivity | 69 (31 OA, 38 FM) | 23 control for OA, 35 control for FM | 51-70y | ↓FC in insula in OA; ↓FC in sensorimotor cortex in FM |
| Gupta 2015[219] | Irritable bowel syndrome (IBS); localised provoked vulvodynia (LPVD) | Resting state functional connectivity | 58 (29 LPVD, 29 IBS) | 29 | 18-30y | Altered FC across sensorimotor and default mode networks in LPVD compared to IBS and controls |
| Delgado-Gallen 2023[220] | Widespread, head or face, upper or lower limb and back pain | Resting state functional connectivity | 133 | 214 | 51-70y | ↔Brain system segregation between groups |
| Hadjikhani 2013[221] | Migraine, carpal tunnel, trigeminal neuralgia | Resting state functional connectivity | 42 (22 migraine, 11 carpal tunnel, 9 trigeminal neuralgia) | 40 | 18-30y; 31-50y | ↑FC between left and right amygdala and anterior insula, secondary somatosensory cortex and thalamus in migraineurs |
| Michels 2017[222] | Medication overuse headache and chronic myofascial pain | Resting state functional connectivity; Fractional anisotropy; Mean diffusivity | 23 (12medication overuse headache and 11 chronic myofascial pain) | 16 | 31-50y | ↓FC in frontoparietal attention network in both pain groups; altered FA and MD in insula in medication overuse headache |

FC: functional connectivity; FA: fractional anisotropy; MD: mean diffusivity; GMV: grey matter volume

## **Table S20: Mixed pain non-imaging study findings**

| Study | Pain type | Primary outcome measure | Number of pain group patients | Number of healthy controls | Age group(s) of patients | Main finding (pain group vs control) |
| --- | --- | --- | --- | --- | --- | --- |
| Schwenkreis 2010[223] | Transcranial magnetic stimulation | Incomplete median or ulnar nerve lesion chronic neuropathic pain, painful osteoarthritis of the hand | 46: 26 neuropathic, 20 OA | 14 | 51-70y | ↓intracortical inhibition in the hemisphere contralateral to lesioned nerve in neuralgia, no difference for other groups |
| Klug 2011[224] | EEG | Fibromyalgia, somatoform pain disorder | 15 | 15 | 31-50y; 51-70y | ↓beta-3 waves in primary and secondary somatosensory, prefrontal, anterior cingulate cortex, amygdala, supplementary motor area, insula, hippocampus, posterior parietal cortex |
| Schuurman 2023[225] | EEG | MSK pain, headache, neuropathic pain, ischaemic pain, abdo pain and multiple | 28 | 44 | 31-50y | ↓power in lower frequency bands |
| TaDinh 2019[226] | EEG | Chronic back pain, chronic widespread pain, joint pain, neuropathic pain, postherpetic neuralgia, polyneuropathic pain | 101 | 84 | 51-70y | ↑connectivity in theta and gamma bands |
| Dimmek 2021[227] | Blood brain derived neurotrophic factor (BDNF) | Chronic widespread pain, osteoarthritis of knee/hip, or chronic lower back pain | Osteoarthritis (37), low back pain (38), chronic widespread pain (37) | 35 | 31-50y; 51-70y | ↓BDNF in patients |
| Sarchielli 2007[228] | Blood brain derived neurotrophic factor (BDNF) | Fibromyalgia and migraine | 20 FM, 20 migraine | 20 | 31-50y | ↑BDNF in patients |
| Stefani 2019[229] | Blood brain derived neurotrophic factor (BDNF) | Endometriosis, fibromyalgia, chronic tension type headache, osteoarthritis | 88 OA, 36 endometriosis, 117 fibromyalgia, 33 chronic tension type headache | 41 | 31-50y; 51-70y | ↑BDNF in all pain conditions particularly in fibromyalgia (FM>headache>OA> endometriosis) |
| Deitos 2015[230] | Blood brain derived neurotrophic factor (BDNF) | Endometriosis, chronic tension headache and myofascial pain syndrome | chronic headache - 30, myofascial - 29, fibromyalgia - 22, OA- 27, endometriosis 32 | 37 | 31-50y | ↑BDNF with central sensitisation syndrome |
| Caumo 2017[231] | Blood brain derived neurotrophic factor (BDNF) | Osteoarthritis (OA), myofascial pain syndrome (MFS), chronic tension type headache, Fibromyalgia (FM) | 222 | 63 | 31-50y; 51-70y | ↑BDNF in all pain conditions compared to controls (FM> MPS>OA) |

EEG: Electroencephalography

## **Table S21: Neck pain study findings**

| Study | Pain type | Primary outcome measure | Number of pain group patients | Number of healthy controls | Age group(s) of patients | Main finding (pain group vs control) |
| --- | --- | --- | --- | --- | --- | --- |
| Li 2024[194] | Degenerative cervical myelopathy | Cerebral blood flow in specific regions | 42 | 40 | 51-70y | ↑BOLD in bilateral thalamus and right insula, ↓BOLD in right somatosensory cortex |
| deZoete 2022[232] | Chronic neck pain | Grey matter volume (including cortical thickness) | 33 | 30 | 31-50y | ↑cortical thickness in parahippocampus, ↓GMV in frontal, parietal, occipital, precentral, paracentral, temporal cortices and precuneus |
| Murillo 2024[233] | Chronic whiplash associated disorders | Grey matter volume (including cortical thickness) | 63 | 32 | 31-50y | ↓GMV in bilateral dorsolateral prefrontal cortex, left inferior temporal gyrus |
| DePauw 2019[234] | Whiplash and idiopathic neck pain | Grey matter volume (including cortical thickness) | 37 Idiopathic neck pain, 39 whiplash | 34 | 18-30y | ↑cortical thickness in left precuneus and ↑GMV in left superior parietal gyrus in CINP compared to whiplash; whiplash had ↓right precentral and superior temporal gyri compared to controls |
| Coppieters 2017[235] | Chronic whiplash associated disease (CWAD) and chronic idiopathic neck pain (CINP) | Grey matter volume (including cortical thickness) | 34 chronic idiopathic neck pain and 31 chronic whiplash-associated disorders | 28 | 31-50y | ↓GMV in right lateral orbitofrontal cortex, left supramarginal cortex, and left posterior cingulate cortex |
| Woodworth 2019[203] | Cervical spondylosis | Grey matter volume (including cortical thickness) | 26 | 45 | 51-70y | ↓cortical thickness in superior frontal gyrus, anterior cingulate, precuneus and ↓GMV in putamen |
| Yang 2020[236] | Cervical spondylosis | Grey matter volume (including cortical thickness) | 31 | 30 | 31-50y | ↓GMV in right middle cingulate cortex, superior temporal gyrus and precuneus |
| Coppieters 2018[237] | chronic idiopathic neck pain (37), chronic whiplash-associated disorders (37) | Grey matter volume (including cortical thickness); Fractional anisotropy; Mean diffusivity | 74 | 31 | 18-30y; 31-50y; 51-70y | ↓cortical thickness in left precuneus in traumatic vs non-traumatic neck pain. ↔No difference between traumatic neck pain compared to controls |
| Ihara 2019[238] | Chronic neck pain | Resting state functional connectivity | 20 | 20 | 31-50y | ↑FC between right dorsolateral prefrontal cortex and anterior insular cortex |
| Ma 2020[239] | Cervical discogenic pain | Resting state functional connectivity | 40 | 40 | 51-70y | ↑ALFF in left insula and posterior precuneus and ↓ALFF in left precentral/postcentral gyrus, thalamus and subgenual anterior cingulate cortex |
| Ni 2022[240] | Chronic neck pain | Resting state functional connectivity | 89 | 57 | 18-30y; 31-50y | Altered FC in anterior cingulate, occipital lobe, temporal lobe and cerebellum |
| Coppieters 2021[241] | Chronic idiopathic neck pain (CINP) and chronic whiplash associated neck pain (CWAD) | Resting state functional connectivity | 38 CINP, 37 CWAD | 32 | 18-30y; 31-50y | ↑FC between left amygdala and left frontal operculum and between left pallidum and frontal operculum; left amygdala and frontal orbital cortex |
| DePauw 2020[242] | Chronic traumatic (whiplash) and idiopathic neck pain | Resting state functional connectivity | 39 with idiopathic neck pain, 37 with whiplash associated disorder | 35 | 18-30y; 31-50y | Altered network in posterior cingulate cortex, amygdala and pallidum |
| Yu 2017[243] | Cervical spondylotic radiculopathy | Resting state functional connectivity | 14 | 14 | 31-50y | ↑FC between periaqueductal grey and right orbital inferior frontal gyrus, supra-marginal gyrus/postcentral gyrus and putamen |
| Yu 2017[244] | Chronic neck and shoulder pain | Resting state functional connectivity | 25 | 20 | 31-50y | No differences in brain structure between groups; ↑ReHo in bilateral middle frontal gyrus and ↓ReHo in left insula, superior frontal gyrus, middle cingulate gyrus, supplementary motor area, right postcentral gyrus and superior parietal lobule |
| Zhang 2024[45] | Chronic neck and shoulder pain | Resting state functional connectivity | 60 | 60 | 31-50y | ↑ReHo in right anterior cingulate cortex, left insular cortex and left Rolandic operculum and ↓ReHo in middle cingulate cortex |
| Bai 2022[245] | Cervical spondylosis | Resting state functional connectivity | 33 | 44 | 51-70y | Altered ALFF in middle cingulate cortex, cerebellum and middle frontal gyrus |
| Chen 2018[246] | Cervical spondylosis | Resting state functional connectivity | 104 | 96 | 18-30y | ↓ReHo in left sensorimotor cortex and right temporo-parietal junction |

ALFF: Amplitude of low frequency fluctuations; ReHo: regional homogeneity; FC: functional connectivity; GMV: grey matter volumes; BOLD: Blood oxygen level dependent oscillations

## **Table S22: Neuropathic pain study findings**

| Study | Pain type | Primary outcome measure | Number of pain group patients | Number of healthy controls | Age group(s) of patients | Main finding (pain group vs control) |
| --- | --- | --- | --- | --- | --- | --- |
| Pandey 2024[247] | Hemisensory Syndrome | Cerebral blood flow in specific regions; Fractional anisotropy; Mean diffusivity | 20 | 10 | 18-30y; 31-50y; 51-70y | ↑cerebral blood flow and FA difference in thalamus of the affected hemisphere in pain group vs non-affected hemisphere but no difference compared to controls |
| Park 2021[248] | Stroke patients who experience central post-stroke pain | Fractional anisotropy | 17 | 34 | 51-70y | ↓FA spinothalamic tract and superior thalamic tract and superior thalamic radiation |
| Scheliga 2024[249] | Small fibre neuropathy | Grey matter volume (including cortical thickness) | 26 | 25 | 31-50y | ↓GMV in bilateral medial superior frontal gyrus and left caudate, ↑GMV left precuneus and Raphe’s nuclei |
| vanGool 2024[250] | Peripheral small fiber neuropathy (SFN): idiopathic (iSFN) vs motation in SCN9A | Grey matter volume (including cortical thickness) | 20 iSFN, 12 SFN-SCN9A | 21 | 31-50y; 51-70y | ↓cortical thickness in left anterior circular sulcus of insula, left and right anterior midcingulate cortex, bilateral dorsal posterior cingulate cortex, ↓GMV left hippocampus |
| Mole 2014[251] | Spinal cord injury (SCI) with and without neuropathic pain | Grey matter volume (including cortical thickness) | 18 | 12 SCI without pain and 18 healthy | 51-70y | No difference in whole brain tissue fractions and ↓white matter in corticospinal tracts |
| Yoon 2013[252] | Chronic neuropathic pain after spinal cord injury | Grey matter volume (including cortical thickness); Mean diffusivity | 10 | 10 | 31-50y | ↓GMV in left dorsolateral prefrontal cortex, bilateral anterior insulae and subgenual anterior cingulate cortices, ↓MD in right internal capsule |
| Gustin 2010[253] | Persisent neuropathic pain after spinal cord injury (SCI) | Mean diffusivity | 12 | 45 | 31-50y | ↑MD in dorsolateral prefrontal cortex, posterior parietal cortex and premotor cortex; ↓MD in ventral posterior thalamus, amygdala and ventral pons; no difference in FA |
| Liu 2022[254] | Breast cancer survivors with chronic neuropathic pain | Resting state functional connectivity | 20 | 20 | 31-50y; 51-70y | ↑FC between the thalamus and superior insula |
| Chao 2023[255] | Small fiber neuropathy (SFN) | Resting state functional connectivity | 53 | 53 | 18-30y; 31-50y; 51-70y; >71y | ↓FC globally, particularly in limbic and somatosensory system |
| Park 2023[256] | Neuropathic pain following spinal cord injury | Resting state functional connectivity | 41 | 32 | 31-50y; 51-70y | ↓ALFF in left middle frontal gyrus |
| Teixeira 2021[257] | Chronic peripheral neuropathic pain (peripheral nerve lesions in lower limbs) | EEG | 12 | 10 | 51-70y | ↓beta power |
| Stern 2006[258] | Chronic neurogenic pain fulfilling criteria for central lateral thalamotomy (trigeminal/lower back/leg) | EEG | 16 | 16 |  | ↑overactivations in theta and low beta frequency ranges particularly in insula, anterior cingulate, prefrontal and inferior posterior parietal cortices |
| Rajan 2024[259] | Neuropathic pain | EEG | 26 | 26 | 31-50y | ↑power spectral densities in delta, theta and beta frequency ranges |
| DiPietro 2018[260] | Orofacial neuropathic pain | EEG | 20 | 20 | 18-30y; 31-50y; 51-70y; >71y | ↑power in the 4-25Hz frequency range, mainly in the theta and low alpha bands |

FA: Fractional anisotropy, ALFF: amplitude of low frequency fluctuations; GMV: grey matter volumes; FC: functional connectivity; EEG: electroencephalography

## **Table S23: Pelvic pain study findings**

| Study | Pain type | Primary outcome measure | Number of pain group patients | Number of healthy controls | Age group(s) of patients | Main finding (pain group vs control) |
| --- | --- | --- | --- | --- | --- | --- |
| Liu 2017[261] | primary dysmenorrhea | Fractional anisotropy; Mean diffusivity | 41 | 41 | 18-30y | ↓FA and AD; ↑MD and RD in posterior section of cingulum bundle and parahippocampal section of cingulum |
| Farmer 2015[262] | Chronic interstitial cystitis/bladder pain syndrome. Note all women | Fractional anisotropy; Mean diffusivity | 22 | 32 | 31-50y | ↑FA in bilateral inferior and superior longitudinal fasciculus; ↓FA in anterior thalamic radiation, forceps major and inferior longitudinal fasciculus |
| Lan 2023[263] | Chronic prostatitis/chronic pelvic pain | Fractional anisotropy; Mean diffusivity | 23 | 22 | 18-30y; 31-50y | ↑FA ↓MD in white matter tracts in corpus callosum, cerebellar and cerebral peduncle, internal and external capsule, corona radiata, thalamic radiation, cingulus, longitudinal and uncinate fasciculi |
| Dun 2017[264] | Primary dysmenorrhoea | Fractional anisotropy; Mean diffusivity | 27 | 24 | 18-30y | ↓FA↑MD and RD in splenium and genu of corpus callosum, bilateral posterior limbs of internal capsule, bilateral superior and posterior corona radiata, left external capsule, fornix, sagittal striatum and bilateral posterior thalamic radiation |
| As-Sanie 2012[265] | Endometriosis-associated chronic pain | Grey matter volume (including cortical thickness) | 17 | 23 | 18-30y; 31-50y; 51-70y | ↑GMV in left amygdala; ↓GMV in left thalamus, left middle frontal gyrus, bilateral mid cingulate cortex, right putamen and right insular cortex |
| Mordasini 2012[266] | Chronic pelvic pain syndrome | Grey matter volume (including cortical thickness) | 20 | 20 | 31-50y | ↓GMV in anterior cingulate cortex |
| Bagarinao 2014[267] | Chronic pelvic pain | Grey matter volume (including cortical thickness) | 33 | 33 | 18-30y; 31-50y; 51-70y | ↓GMV in primary somatosensory cortex, pre-supplementary motor area, hippocampus and amygdala |
| Kairys 2015[268] | Interstitial cystitis | Grey matter volume (including cortical thickness) | 33 | 33 | 31-50y | ↑GMV in right primary somatosensory cortex, superior parietal lobule bilaterally and right supplementary motor area |
| Maulitz 2024[269] | Endometriosis | Grey matter volume (including cortical thickness) | 27 endometriosis pelvic pain, 26 no endometriosis pelvic pain | 25 | 18-30y | ↑GMV in left cerebellum, lingual or calcarine gyrus; ↓GMV right cerebellum |
| Schweinhardt 2008[270] | Provoked vestibulodynia (PVD) | Grey matter volume (including cortical thickness) | 14 | 14 | 18-30y | ↑GMV in parahippocampal gyrus, hippocampus and basal ganglia |
| Farmer 2011[271] | Chronic prostatitis / Chronic pelvic pain | Grey matter volume (including cortical thickness); Resting state functional connectivity; Fractional anisotropy | 19 | 16 | 31-50y | No group differences in regional GMV; ↑FC in right anterior insula |
| Liu 2023[156] | Primary dysmenorrhoea | Resting state functional connectivity | 33 | 36 | 18-30y | Altered FC in a series of brain regions including the hippocampus, temporal pole, superior temporal gyrus, nucleus accumbens and anterior cingulate cortex |
| Kilpatrick 2014[272] | Female interstitial cystitis/painful bladder syndrome | Resting state functional connectivity | 82 | 85 | 31-50y | ↑FC between paracentral lobule and midbrain and cerebellum |
| Yu 2024[273] | Primary dysmenorrhoea | Resting state functional connectivity | 41 | 39 | 18-30y | ↑FC in bilateral lingual gyrus, dorsal anterior cingulate cortex, middle cingulate cortex, ↓FC in right orbital frontal cortex |
| Lee 2018[274] | Primary dysmenorrhoea | Resting state functional connectivity | 57 | 62 | 18-30y | No difference between groups |
| As-Sanie 2016[275] | Endometriosis-associated chronic pelvic pain | Resting state functional connectivity | 17 | 24 | 18-30y; 31-50y; 51-70y | ↑FC anterior insula to medial prefrontal cortex |
| Ge 2021[276] | Chronic prostatitis/chronic pelvic pain syndrome | Resting state functional connectivity | 18 | 21 | 31-50y | ↓FC between left medial prefrontal cortex and posterior default mode network; ↑FC between left anterior cerebellar lobe and posterior default mode network |
| Kutch 2015[277] | Men with chronic prostatitis/pelvic pain syndrome | Resting state functional connectivity | 28 | 27 | 31-50y | ↑FC between pelvic motor and right posterior insula |
| Lan 2022[278] | Chronic prostatitis and pelvic pain syndrome | Resting state functional connectivity | 28 | 28 | 18-30y; 31-50y | ↓FC between bilateral caudate and right middle cingulate cortex; left globus pallidum to right middle cingulate cortex and left superior temporal gyrus; left putamen to superior temporal gyrus, right middle cingulate cortex and right supramarginal gyrus |
| Lin 2017[279] | Chronic prostatitis/chronic pelvic pain syndrome | Resting state functional connectivity | 31 | 31 | 31-50y | ↓ReHo in bilateral anterior cingulate cortices, insulae and right medial prefrontal cortex, ↑ReHo in brainstem and right thalamus |
| Martucci 2015[280] | Urological chronic pelvic pain syndrome | Resting state functional connectivity | 45 | 45 | 31-50y | ↓FC of default mode network to posterior cingulate cortex and left precuneus, ↑FC between posterior cingulate and insular cortex, dorsolateral prefrontal cortex, thalamus, globus pallidus, putamen, amygdala and hippocampus |
| Wei 2016[281] | Primary dysmenorrhoea | Resting state functional connectivity | 46 | 49 | 18-30y | ↑FC between periaqueductal grey to primary somatosensory and motor cortex, and supplementary motor area |
| Wu 2016[282] | Primary dysmenorrhoea | Resting state functional connectivity | 46 | 49 | 18-30y | ↓ReHo in ventromedial prefrontal cortex, default mode network |
| Gupta 2019[283] | Urological chronic pelvic pain syndrome | Resting state functional connectivity | 85 | 86 | 31-50y | ↓FC in right anterior insula |
| Han 2019[284] | Primary dysmenorrhoea | Resting state functional connectivity | 36 | 29 | 18-30y | ↑serum BDNF, abnormal FC in thalamic subregions to prefrontal, sensorimotor and temporal cortex |
| Ding 2018[285] | Endometriosis | Blood brain derived neurotrophic factor (BDNF) | 23 | 38 | 31-50y | No difference between groups |

GMV: grey matter volume; FC: functional connectivity; FA: fractional anisotropy, AD: axial diffusivity; MD: mean diffusivity; RD: radial diffusivity; ReHo: regional homogeneity; BDNF: brain derived neurotrophic factor

## **Table S24: Postherpetic neuralgia study findings**

| Study | Primary outcome measure | Number of pain group patients | Number of healthy controls | Age group(s) of patients | Main finding (pain group vs control) |
| --- | --- | --- | --- | --- | --- |
| Liu 2019[286] | Grey matter volume (including cortical thickness) | 22 | 28 | 51-70y | ↓GMV in bilateral insular lobes and ↑GMV in bilateral thalamus for HZ compared to controls; ↓GMV in bilateral insulae, right middle frontal gyrus, bilateral precentral gyrus and left postcentral gyrus and ↑GMV in left cerebellar posterior lobe, right parahippocampal gyrus and right lentiform nucleus for PHN compared to controls |
| Liu 2022[287] | Grey matter volume (including cortical thickness) | 30 | 30 healthy, 30 herpes zoster no pain | 51-70y | ↑cortical thickness of bilateral primary visual cortex; left somatosensory cortex, right anterior cingulate gyrus and medial prefrontal cortex, ↓cortical thickness of left insula, frontal opercular, left motor and right superior temporal visual cortices |
| Li 2020[288] | Grey matter volume (including cortical thickness); Resting state functional connectivity | 24 | 23 | 51-70y | ↓GMV of thalamus and amygdala |
| Niu 2022[289] | Grey matter volume (including cortical thickness); White matter hyperintensities; Total brain volume | 25 | 25 | 51-70y | ↓GMV globally, and in striatum, cerebellum precentral, middle frontal, parahippocampal and postcentral gyri |
| Cao 2017[290] | Resting state functional connectivity | 19 | 19 | 31-50y; 51-70y; >71y | ↑ReHo in right anterior and posterior cerebellum, pons, bilateral temporal and frontal gyri, limbic lobe; ↓ReHo in bilateral parietal lobules, right precentral gyrus, left cerebellum posterior and left fusiform gyrus and limbic lobe |
| Cao 2017[291] | Resting state functional connectivity | 23 | 55 | 51-70y | Altered ALFF in pain related regions such as thalamus, insula, cerebellum brainstem |
| Wu 2022[292] | Resting state functional connectivity | 33 PHN, 33 Recuperation from herpes zoster | 34 | 51-70y | Altered default mode network connectivity to salience and basal ganglia networks |
| Dai 2020[293] | Resting state functional connectivity; Fractional anisotropy | 12 | 12 | 51-70y | ↓FA in right cerebellum, left anterior cingulate, left middle frontal gyrus, bilateral putamen, bilateral temporal lobe, left inferior parietal gyrus and right middle cingulate gyrus. ↑MD in left precuneus and bilateral parietal lobes etc. ↓ALFF in brainstem, insula, superior temporal gyrus, corpus callosum, ↑ALFF in inferior and middle temporal gyri, right superior parietal and left postcentral gyri. |

GMV: grey matter volume; FC: functional connectivity; FA: fractional anisotropy; MD: mean diffusivity; ReHo: regional homogeneity; PHN: postherpetic neuralgia; HZ: herpes zoster; ALFF: amplitude of low frequency fluctuations

## **Table S25: Somatoform pain syndrome study findings**

| Study | Primary outcome measure | Number of pain group patients | Number of healthy controls | Age group(s) of patients | Main finding (pain group vs control) |
| --- | --- | --- | --- | --- | --- |
| Magon 2018[294] | Grey matter volume (including cortical thickness) | 23 | 23 | 51-70y | ↓cortical thickness in left precentral and postcentral gyri and left inferior temporal sulcus |
| Yoshino 2014[295] | Resting state functional connectivity | 9 | 20 | 31-50y | Atypical precentral gyrus activation |
| Huang 2016[296] | Resting state functional connectivity | 13 | 23 | 31-50y | ↓ReHo in bilateral somatosensory cortices, posterior cerebellum and occipital lobe; ↑ReHo in left superior and inferior frontal gyri, bilateral middle frontal gyri, medial prefrontal cortex, bilateral inferior parietal lobules and precuneus |
| Sun 2020[297] | Resting state functional connectivity | 13 | 23 | 31-50y | ↑FC between left thalamus and right amygdala, hippocampus and occipital lobe |
| Otti 2013[298] | Resting state functional connectivity | 21 | 19 | 31-50y | ↑ALFF between frontoinsular salience network and anterior default mode network |
| Otti 2013[299] | Resting state functional connectivity | 21 | 19 | 31-50y | No group differences |
| Liu 2019[300] | Resting state functional connectivity | 21 | 17 | 31-50y | ↓FC in right inferior occipital gyrus and left calcarine fissures |
| Yoshino 2017[301] | Resting state functional connectivity | 41 | 41 | 31-50y | ↓ReHo in dorsolateral prefrontal cortex and ↓FC between dorsolateral prefrontal cortex and thalamus |
| Ye 2019[302] | EEG | 17 | 17 | 31-50y | ↑resting state alpha oscillations in parietal region |

GMV: grey matter volume; FC: functional connectivity; FA: fractional anisotropy; MD: mean diffusivity; ReHo: regional homogeneity; EEG: electroencephalography; ALFF: amplitude of low frequency fluctuations

## **Table S26: Other pain study findings**

| Study | Pain type | Primary outcome measure | Number of pain group patients | Number of healthy controls | Age group(s) of patients | Main finding (pain group vs control) |
| --- | --- | --- | --- | --- | --- | --- |
| Soros 2020[303] | Chronic non cancer pain | Brain age gap calculations | 59 | 60 | 51-70y | No difference in predicted brain age between groups |
| Kim 2015[304] | Chronic central pain in patients with mild traumatic brain injury | Fractional anisotropy; Mean diffusivity | 22 | 21 | 31-50y | ↓FA↑MD in spinothalamocortical tract |
| Seo 2019[305] | Unilateral arm amputation phantom limb pain | Mean diffusivity | 10 | 16 | 31-50y | ↑MD in internal capsule, posterior thalamic radiation, sagittal striatum, corona radiata, cerebral peduncle, external capsule, superior frontooccipital fasciculus, fornix and tapetum |
| Zhao 2023[306] | Multisite chronic pain | Grey matter volume (including cortical thickness) | 89,991 | 188,746 | 51-70y | ↑dementia risk, hippocampal atrophy, broader and faster cognitive impairment, worse in multisite chronic pain compared to single site or healthy controls |
| Riederer 2017[307] | Chronic pain with and without non- dermatomal sensory deficits | Grey matter volume (including cortical thickness) | 25 NDSDs, 23 pain only | 29 | 31-50y | ↑GMV in right and left insula with extension to the adjacent superior temporal gyrus |
| Valet 2009[308] | Pain disorder (DSM-IV); chronic pain at one or more body sites | Grey matter volume (including cortical thickness) | 14 | 25 | 51-70y | ↓GMV in prefrontal cortex, middle and superior frontal cortex, anterior and posterior cingulate cortex, insula, parahippocampal and inferior temporal cortex and cerebellum |
| Polli 2016[309] | Parkinson's disease with persistent pain | Resting state functional connectivity | 20 | 20 PD without pain, 15 HC | 51-70y | ↓↑FC in right nucleus accumbens and left hippocampus |
| Shen 2022[310] | Any chronic pain associated with Parkinson's disease | Resting state functional connectivity | 15 | 18 | 51-70y | ↓FC in prefrontal cortex and cingulum cortex |
| Zhou 2022[311] | Lung cancer patients with bone metastatic pain | Resting state functional connectivity | 27 | 27 cancer no pain, 27 HC | 51-70y | No difference in functional connectivity between groups |
| Wei 2024[312] | Lung cancer patients with cancer pain | Resting state functional connectivity | 50 | 50 cancer no pain, 31 HC | 51-70y | ↓FC particularly in somatomotor and ventral attention, frontoparietal and default mode networks |
| Flowers 2021[313] | Mild traumatic brain injury headache | Resting state functional connectivity | 83 | 35 | 31-50y | Mild pain had ↑FC in premotor cortices, moderate pain and severe pain had ↓FC in parietal cortex, right insula, premotor cortices and secondary somatosensory cortex |
| Karafin 2019[314] | Sickle cell disease with pain | Resting state functional connectivity | 15 | 7 SCD no chronic pain, 10 HC | 18-30y | ↓FC between periaqueductal grey and anterior cingulate cortex, ↑FC between the periaqueductal grey and occipital and parietal lobes |
| You 2024[315] | Chronic primary pain | EEG | 20 | 20 | 31-50y | ↓transition to different microstates at rest |
| Jensen 2013[316] | Spinal cord injury chronic pain | EEG | 38 | 16 SCI no pain, 28 HC | 31-50y; 51-70y | ↓alpha activity and ↑theta activity |
| Lopes 2024[317] | Hip osteonecrosis secondary to sickle cell disease | EEG | 24 | 19 | 31-50y | ↑delta and theta activity |

GMV: grey matter volume; FC: functional connectivity; FA: fractional anisotropy; MD: mean diffusivity; EEG: electroencephalograph

# **References**

1. Hong J-Y, Kilpatrick LA, Labus J, Gupta A, Jiang Z, Ashe-McNalley C, et al. Patients with chronic visceral pain show sex-related alterations in intrinsic oscillations of the resting brain. *The Journal of neuroscience : the official journal of the Society for Neuroscience* 2013;**33**: 11994-2002.<https://dx.doi.org/10.1523/JNEUROSCI.5733-12.2013>

2. Chen JY-W, Blankstein U, Diamant NE, Davis KD. White matter abnormalities in irritable bowel syndrome and relation to individual factors. *Brain research* 2011;**1392**: 121-31.<https://dx.doi.org/10.1016/j.brainres.2011.03.069>

3. Frokjaer JB, Olesen SS, Gram M, Yavarian Y, Bouwense SAW, Wilder-Smith OHG, et al. Altered brain microstructure assessed by diffusion tensor imaging in patients with chronic pancreatitis. *Gut* 2011;**60**: 1554-62.<https://dx.doi.org/10.1136/gut.2010.236620>

4. Ellingson BM, Mayer E, Harris RJ, Ashe-McNally C, Naliboff BD, Labus JS, et al. Diffusion tensor imaging detects microstructural reorganization in the brain associated with chronic irritable bowel syndrome. *Pain* 2013;**154**: 1528-41.<https://dx.doi.org/10.1016/j.pain.2013.04.010>

5. Chua CS, Bai C-H, Shiao C-Y, Hsu C-Y, Cheng C-W, Yang K-C, et al. Negative correlation of cortical thickness with the severity and duration of abdominal pain in Asian women with irritable bowel syndrome. *PloS one* 2017;**12**: e0183960.<https://dx.doi.org/10.1371/journal.pone.0183960>

6. Barazanji N, Paul Hamilton J, Icenhour A, Simon RA, Bednarska O, Tapper S, et al. Irritable bowel syndrome in women: Association between decreased insular subregion volumes and gastrointestinal symptoms. *NeuroImage Clinical* 2022;**35**: 103128.<https://dx.doi.org/10.1016/j.nicl.2022.103128>

7. Frokjaer JB, Bouwense SAW, Olesen SS, Lundager FH, Eskildsen SF, van Goor H, et al. Reduced cortical thickness of brain areas involved in pain processing in patients with chronic pancreatitis. *Clinical gastroenterology and hepatology : the official clinical practice journal of the American Gastroenterological Association* 2012;**10**: 434-8.e1.<https://dx.doi.org/10.1016/j.cgh.2011.11.024>

8. Jiang Z, Dinov ID, Labus J, Shi Y, Zamanyan A, Gupta A, et al. Sex-related differences of cortical thickness in patients with chronic abdominal pain. *PloS one* 2013;**8**: e73932.<https://dx.doi.org/10.1371/journal.pone.0073932>

9. Ohlmann H, Koenen LR, Labrenz F, Engler H, Theysohn N, Langhorst J, et al. Altered Brain Structure in Chronic Visceral Pain: Specific Differences in Gray Matter Volume and Associations With Visceral Symptoms and Chronic Stress. *Frontiers in neurology* 2021;**12**: 733035.<https://dx.doi.org/10.3389/fneur.2021.733035>

10. Grinsvall C, Ryu HJ, Van Oudenhove L, Labus JS, Gupta A, Ljungberg M, et al. Association between pain sensitivity and gray matter properties in the sensorimotor network in women with irritable bowel syndrome. *Neurogastroenterology and motility* 2021;**33**: e14027.<https://dx.doi.org/10.1111/nmo.14027>

11. Prus MS, Bayer A, Bayer K-E, Schumann M, Atreya R, Mekle R, et al. Functional Brain Changes Due to Chronic Abdominal Pain in Inflammatory Bowel Disease: A Case-Control Magnetic Resonance Imaging Study. *Clinical and translational gastroenterology* 2022;**13**: e00453.<https://dx.doi.org/10.14309/ctg.0000000000000453>

12. Chen F, Zhang S, Li P, Xu K, Liu C, Geng B, et al. Disruption of Periaqueductal Gray-default Mode Network Functional Connectivity in Patients with Crohn's Disease with Abdominal Pain. *Neuroscience* 2023;**517**: 96-104.<https://dx.doi.org/10.1016/j.neuroscience.2023.03.002>

13. Qi R, Shi Z, Weng Y, Yang Y, Zhou Y, Surento W, et al. Similarity and diversity of spontaneous brain activity in functional dyspepsia subtypes. *Acta radiologica (Stockholm, Sweden : 1987)* 2020;**61**: 927-35.<https://dx.doi.org/10.1177/0284185119883391>

14. Hong J-Y, Kilpatrick LA, Labus JS, Gupta A, Katibian D, Ashe-McNalley C, et al. Sex and disease-related alterations of anterior insula functional connectivity in chronic abdominal pain. *The Journal of neuroscience : the official journal of the Society for Neuroscience* 2014;**34**: 14252-9.<https://dx.doi.org/10.1523/JNEUROSCI.1683-14.2014>

15. Bao C-H, Liu P, Liu H-R, Wu L-Y, Jin X-M, Wang S-Y, et al. Differences in regional homogeneity between patients with Crohn's disease with and without abdominal pain revealed by resting-state functional magnetic resonance imaging. *Pain* 2016;**157**: 1037-44.<https://dx.doi.org/10.1097/j.pain.0000000000000479>

16. de Vries M, Wilder-Smith OH, Jongsma MLA, van den Broeke EN, Arns M, van Goor H, et al. Altered resting state EEG in chronic pancreatitis patients: toward a marker for chronic pain. *Journal of pain research* 2013;**6**: 815-24.<https://dx.doi.org/10.2147/JPR.S50919>

17. Olesen SS, Hansen TM, Graversen C, Steimle K, Wilder-Smith OHG, Drewes AM. Slowed EEG rhythmicity in patients with chronic pancreatitis: evidence of abnormal cerebral pain processing? *European journal of gastroenterology & hepatology* 2011;**23**: 418-24.<https://dx.doi.org/10.1097/MEG.0b013e3283457b09>

18. Gu S-Y, Shi F-C, Wang S, Wang C-Y, Yao X-X, Sun Y-F, et al. Altered cortical thickness and structural covariance networks in chronic low back pain. *Brain research bulletin* 2024;**212**: 110968.<https://dx.doi.org/10.1016/j.brainresbull.2024.110968>

19. Apkarian AV, Sosa Y, Sonty S, Levy RM, Harden RN, Parrish TB, et al. Chronic back pain is associated with decreased prefrontal and thalamic gray matter density. *The Journal of neuroscience : the official journal of the Society for Neuroscience* 2004;**24**: 10410-5.<https://dx.doi.org/10.1523/JNEUROSCI.2541-04.2004>

20. Schmidt-Wilcke T, Leinisch E, Ganssbauer S, Draganski B, Bogdahn U, Altmeppen J, et al. Affective components and intensity of pain correlate with structural differences in gray matter in chronic back pain patients. *Pain* 2006;**125**: 89-97.<https://dx.doi.org/10.1016/j.pain.2006.05.004>

21. Fritz H-C, McAuley JH, Wittfeld K, Hegenscheid K, Schmidt CO, Langner S, et al. Chronic Back Pain Is Associated With Decreased Prefrontal and Anterior Insular Gray Matter: Results From a Population-Based Cohort Study. *The journal of pain* 2016;**17**: 111-8.<https://dx.doi.org/10.1016/j.jpain.2015.10.003>

22. Mansour ZM, Lepping RJ, Honea RA, Brooks WM, Yeh H-W, Burns JM, et al. Structural Brain Imaging in People With Low Back Pain. *Spine* 2017;**42**: 726-32.<https://dx.doi.org/10.1097/BRS.0000000000001915>

23. Murray K, Lin Y, Makary MM, Whang PG, Geha P. Brain Structure and Function of Chronic Low Back Pain Patients on Long-Term Opioid Analgesic Treatment: A Preliminary Study. *Molecular pain* 2021;**17**: 1744806921990938.<https://dx.doi.org/10.1177/1744806921990938>

24. Buckalew N, Haut MW, Morrow L, Weiner D. Chronic pain is associated with brain volume loss in older adults: preliminary evidence. *Pain medicine (Malden, Mass)* 2008;**9**: 240-8.<https://dx.doi.org/10.1111/j.1526-4637.2008.00412.x>

25. Ivo R, Nicklas A, Dargel J, Sobottke R, Delank K-S, Eysel P, et al. Brain structural and psychometric alterations in chronic low back pain. *European spine journal : official publication of the European Spine Society, the European Spinal Deformity Society, and the European Section of the Cervical Spine Research Society* 2013;**22**: 1958-64.<https://dx.doi.org/10.1007/s00586-013-2692-x>

26. Sacca V, Chai-Zhang TC, Hodges S, Amores J, Guler S, Todorova N, et al. Morphological changes of the limbic system associated with acute and chronic low-back pain: A UK biobank imaging study. *European journal of pain (London, England)* 2024;**28**: 608-19.<https://dx.doi.org/10.1002/ejp.2206>

27. Luchtmann M, Steinecke Y, Baecke S, Lützkendorf R, Bernarding J, Kohl J, et al. Structural Brain Alterations in Patients with Lumbar Disc Herniation: A Preliminary Study. *PLOS ONE* 2014;**9**: e90816.10.1371/journal.pone.0090816

28. Nees F, Ruttorf M, Fuchs X, Rance M, Beyer N. Volumetric brain correlates of approach-avoidance behavior and their relation to chronic back pain. *Brain imaging and behavior* 2020;**14**: 1758-68.<https://dx.doi.org/10.1007/s11682-019-00110-x>

29. Kong J, Spaeth RB, Wey H-Y, Cheetham A, Cook AH, Jensen K, et al. S1 is associated with chronic low back pain: a functional and structural MRI study. *Molecular pain* 2013;**9**: 43.<https://dx.doi.org/10.1186/1744-8069-9-43>

30. Lamichhane B, Jayasekera D, Jakes R, Ray WZ, Leuthardt EC, Hawasli AH. Functional Disruptions of the Brain in Low Back Pain: A Potential Imaging Biomarker of Functional Disability. *Frontiers in neurology* 2021;**12**: 669076.<https://dx.doi.org/10.3389/fneur.2021.669076>

31. Wei X, Wang L, Yu F, Lee C, Liu N, Ren M, et al. Identifying the neural marker of chronic sciatica using multimodal neuroimaging and machine learning analyses. *Frontiers in neuroscience* 2022;**16**: 1036487.<https://dx.doi.org/10.3389/fnins.2022.1036487>

32. Zhang B, Jung M, Tu Y, Gollub R, Lang C, Ortiz A, et al. Identifying brain regions associated with the neuropathology of chronic low back pain: a resting-state amplitude of low-frequency fluctuation study. *British journal of anaesthesia* 2019;**123**: e303-e11.<https://dx.doi.org/10.1016/j.bja.2019.02.021>

33. Dolman AJ, Loggia ML, Edwards RR, Gollub RL, Kong J, Napadow V, et al. Phenotype matters: the absence of a positive association between cortical thinning and chronic low back pain when controlling for salient clinical variables. *The Clinical journal of pain* 2014;**30**: 839-45.<https://dx.doi.org/10.1097/AJP.0000000000000043>

34. Ung H, Brown JE, Johnson KA, Younger J, Hush J, Mackey S. Multivariate classification of structural MRI data detects chronic low back pain. *Cerebral cortex (New York, NY : 1991)* 2014;**24**: 1037-44.<https://dx.doi.org/10.1093/cercor/bhs378>

35. Li H, Song Q, Zhang R, Zhou Y, Kong Y. Enhanced Temporal Coupling between Thalamus and Dorsolateral Prefrontal Cortex Mediates Chronic Low Back Pain and Depression. *Neural plasticity* 2021;**2021**: 7498714.<https://dx.doi.org/10.1155/2021/7498714>

36. Baumbach P, Meisner W, Reichenbach JR, Gussew A. Functional connectivity and neurotransmitter impairments of the salience brain network in chronic low back pain patients: a combined resting-state functional magnetic resonance imaging and 1 H-MRS study. *Pain* 2022;**163**: 2337-47.<https://dx.doi.org/10.1097/j.pain.0000000000002626>

37. Ng SK, Urquhart DM, Fitzgerald PB, Cicuttini FM, Kirkovski M, Maller JJ, et al. Examining resting-state functional connectivity in key hubs of the default mode network in chronic low back pain. *Scandinavian journal of pain* 2021;**21**: 839-46.<https://dx.doi.org/10.1515/sjpain-2020-0184>

38. Tu Y, Jung M, Gollub RL, Napadow V, Gerber J, Ortiz A, et al. Abnormal medial prefrontal cortex functional connectivity and its association with clinical symptoms in chronic low back pain. *Pain* 2019;**160**: 1308-18.<https://dx.doi.org/10.1097/j.pain.0000000000001507>

39. Chen Y, Yang Y, Gong Z, Kang Y, Zhang Y, Chen H, et al. Altered effective connectivity from cerebellum to motor cortex in chronic low back pain: A multivariate pattern analysis and spectral dynamic causal modeling study. *Brain research bulletin* 2023;**204**: 110794.<https://dx.doi.org/10.1016/j.brainresbull.2023.110794>

40. Liu J, Zhang F, Liu X, Zhuo Z, Wei J, Du M, et al. Altered small-world, functional brain networks in patients with lower back pain. *Science China Life sciences* 2018;**61**: 1420-4.<https://dx.doi.org/10.1007/s11427-017-9108-6>

41. Zhu K, Chang J, Zhang S, Li Y, Zuo J, Ni H, et al. The enhanced connectivity between the frontoparietal, somatomotor network and thalamus as the most significant network changes of chronic low back pain. *NeuroImage* 2024;**290**: 120558.<https://dx.doi.org/10.1016/j.neuroimage.2024.120558>

42. Shen W, Tu Y, Gollub RL, Ortiz A, Napadow V, Yu S, et al. Visual network alterations in brain functional connectivity in chronic low back pain: A resting state functional connectivity and machine learning study. *NeuroImage Clinical* 2019;**22**: 101775.<https://dx.doi.org/10.1016/j.nicl.2019.101775>

43. Jiang Y, Oathes D, Hush J, Darnall B, Charvat M, Mackey S, et al. Perturbed connectivity of the amygdala and its subregions with the central executive and default mode networks in chronic pain. *Pain* 2016;**157**: 1970-8.<https://dx.doi.org/10.1097/j.pain.0000000000000606>

44. Fan N, Chen J, Zhao B, Liu L, Yang W, Chen X, et al. Neural correlates of central pain sensitization in chronic low back pain: a resting-state fMRI study. *Neuroradiology* 2023;**65**: 1767-76.<https://dx.doi.org/10.1007/s00234-023-03237-3>

45. Zhang S, Zhang G, Bao S, Tan J, He R, Wang H, et al. Resting-State Functional Connectivity of the Cerebellum in Patients with Chronic Low Back Pain. *American journal of physical medicine & rehabilitation* 2024.<https://dx.doi.org/10.1097/PHM.0000000000002583>

46. Tagliazucchi E, Balenzuela P, Fraiman D, Chialvo DR. Brain resting state is disrupted in chronic back pain patients. *Neuroscience letters* 2010;**485**: 26-31.<https://dx.doi.org/10.1016/j.neulet.2010.08.053>

47. Hotta J, Zhou G, Harno H, Forss N, Hari R. Complex regional pain syndrome: The matter of white matter? *Brain and behavior* 2017;**7**: e00647.<https://dx.doi.org/10.1002/brb3.647>

48. Im JJ, Kim J, Jeong H, Oh JK, Lee S, Lyoo IK, et al. Prefrontal White Matter Abnormalities Associated With Pain Catastrophizing in Patients With Complex Regional Pain Syndrome. *Archives of physical medicine and rehabilitation* 2021;**102**: 216-24.<https://dx.doi.org/10.1016/j.apmr.2020.07.006>

49. Pleger B, Draganski B, Schwenkreis P, Lenz M, Nicolas V, Maier C, et al. Complex regional pain syndrome type I affects brain structure in prefrontal and motor cortex. *PloS one* 2014;**9**: e85372.<https://dx.doi.org/10.1371/journal.pone.0085372>

50. Barad MJ, Ueno T, Younger J, Chatterjee N, Mackey S. Complex regional pain syndrome is associated with structural abnormalities in pain-related regions of the human brain. *The journal of pain* 2014;**15**: 197-203.<https://dx.doi.org/10.1016/j.jpain.2013.10.011>

51. Lee D-H, Lee K-J, Cho KIK, Noh EC, Jang JH, Kim YC, et al. Brain alterations and neurocognitive dysfunction in patients with complex regional pain syndrome. *The journal of pain* 2015;**16**: 580-6.<https://dx.doi.org/10.1016/j.jpain.2015.03.006>

52. Domin M, Strauss S, McAuley JH, Lotze M. Complex Regional Pain Syndrome: Thalamic GMV Atrophy and Associations of Lower GMV With Clinical and Sensorimotor Performance Data. *Frontiers in neurology* 2021;**12**: 722334.<https://dx.doi.org/10.3389/fneur.2021.722334>

53. Shokouhi M, Clarke C, Morley-Forster P, Moulin DE, Davis KD, St Lawrence K. Structural and Functional Brain Changes at Early and Late Stages of Complex Regional Pain Syndrome. *The journal of pain* 2018;**19**: 146-57.<https://dx.doi.org/10.1016/j.jpain.2017.09.007>

54. Geha PY, Baliki MN, Harden RN, Bauer WR, Parrish TB, Apkarian AV. The brain in chronic CRPS pain: abnormal gray-white matter interactions in emotional and autonomic regions. *Neuron* 2008;**60**: 570-81.<https://dx.doi.org/10.1016/j.neuron.2008.08.022>

55. van Velzen GAJ, Rombouts SARB, van Buchem MA, Marinus J, van Hilten JJ. Is the brain of complex regional pain syndrome patients truly different? *European journal of pain (London, England)* 2016;**20**: 1622-33.<https://dx.doi.org/10.1002/ejp.882>

56. Di Pietro F, Lee B, Henderson LA. Altered resting activity patterns and connectivity in individuals with complex regional pain syndrome. *Human brain mapping* 2020;**41**: 3781-93.<https://dx.doi.org/10.1002/hbm.25087>

57. Bolwerk A, Seifert F, Maihofner C. Altered resting-state functional connectivity in complex regional pain syndrome. *The journal of pain* 2013;**14**: 1107-15.e8.<https://dx.doi.org/10.1016/j.jpain.2013.04.007>

58. Hong H, Suh C, Namgung E, Ha E, Lee S, Kim RY, et al. Aberrant Resting-state Functional Connectivity in Complex Regional Pain Syndrome: A Network-based Statistics Analysis. *Experimental neurobiology* 2023;**32**: 110-8.<https://dx.doi.org/10.5607/en23003>

59. Kim J-H, Choi S-H, Jang JH, Lee D-H, Lee K-J, Lee WJ, et al. Impaired insula functional connectivity associated with persistent pain perception in patients with complex regional pain syndrome. *PloS one* 2017;**12**: e0180479.<https://dx.doi.org/10.1371/journal.pone.0180479>

60. Hok P, Strauss S, McAuley J, Domin M, Wang AP, Rae C, et al. Functional connectivity in complex regional pain syndrome: A bicentric study. *NeuroImage* 2024;**301**: 120886.<https://dx.doi.org/10.1016/j.neuroimage.2024.120886>

61. Sano M, Iwatsuki K, Hirata H, Hoshiyama M. Imbalance in positive and negative acceleration ratio of alpha oscillation in patients with complex regional pain syndrome. *Heliyon* 2024;**10**: e36463.<https://dx.doi.org/10.1016/j.heliyon.2024.e36463>

62. Cauda F, Sacco K, Duca S, Cocito D, D'Agata F, Geminiani GC, et al. Altered resting state in diabetic neuropathic pain. *PloS one* 2009;**4**: e4542.<https://dx.doi.org/10.1371/journal.pone.0004542>

63. Cauda F, Sacco K, D'Agata F, Duca S, Cocito D, Geminiani G, et al. Low-frequency BOLD fluctuations demonstrate altered thalamocortical connectivity in diabetic neuropathic pain. *BMC neuroscience* 2009;**10**: 138.<https://dx.doi.org/10.1186/1471-2202-10-138>

64. Cauda F, D'Agata F, Sacco K, Duca S, Cocito D, Paolasso I, et al. Altered resting state attentional networks in diabetic neuropathic pain. *Journal of neurology, neurosurgery, and psychiatry* 2010;**81**: 806-11.<https://dx.doi.org/10.1136/jnnp.2009.188631>

65. Croosu SS, Roikjer J, Morch CD, Ejskjaer N, Frokjaer JB, Hansen TM. Alterations in Functional Connectivity of Thalamus and Primary Somatosensory Cortex in Painful and Painless Diabetic Peripheral Neuropathy. *Diabetes care* 2023;**46**: 173-82.<https://dx.doi.org/10.2337/dc22-0587>

66. Chao C-C, Hsieh P-C, Janice Lin C-H, Huang S-L, Hsieh S-T, Chiang M-C. Impaired brain network architecture as neuroimaging evidence of pain in diabetic neuropathy. *Diabetes research and clinical practice* 2022;**186**: 109833.<https://dx.doi.org/10.1016/j.diabres.2022.109833>

67. Moayedi M, Weissman-Fogel I, Salomons TV, Crawley AP, Goldberg MB, Freeman BV, et al. White matter brain and trigeminal nerve abnormalities in temporomandibular disorder. *Pain* 2012;**153**: 1467-77.<https://dx.doi.org/10.1016/j.pain.2012.04.003>

68. Budd AS, Huynh TKT, Seres P, Beaulieu C, Armijo-Olivo S, Cummine J. White Matter Diffusion Properties in Chronic Temporomandibular Disorders: An Exploratory Analysis. *Frontiers in pain research (Lausanne, Switzerland)* 2022;**3**: 880831.<https://dx.doi.org/10.3389/fpain.2022.880831>

69. Gerstner G, Ichesco E, Quintero A, Schmidt-Wilcke T. Changes in regional gray and white matter volume in patients with myofascial-type temporomandibular disorders: a voxel-based morphometry study. *Journal of orofacial pain* 2011;**25**: 99-106

70. Schmidt-Wilcke T, Hierlmeier S, Leinisch E. Altered regional brain morphology in patients with chronic facial pain. *Headache* 2010;**50**: 1278-85.<https://dx.doi.org/10.1111/j.1526-4610.2010.01637.x>

71. Wilcox SL, Gustin SM, Macey PM, Peck CC, Murray GM, Henderson LA. Anatomical changes within the medullary dorsal horn in chronic temporomandibular disorder pain. *NeuroImage* 2015;**117**: 258-66.<https://dx.doi.org/10.1016/j.neuroimage.2015.05.014>

72. Gustin SM, Peck CC, Macey PM, Murray GM, Henderson LA. Unraveling the effects of plasticity and pain on personality. *The journal of pain* 2013;**14**: 1642-52.<https://dx.doi.org/10.1016/j.jpain.2013.08.005>

73. Moayedi M, Weissman-Fogel I, Crawley AP, Goldberg MB, Freeman BV, Tenenbaum HC, et al. Contribution of chronic pain and neuroticism to abnormal forebrain gray matter in patients with temporomandibular disorder. *NeuroImage* 2011;**55**: 277-86.<https://dx.doi.org/10.1016/j.neuroimage.2010.12.013>

74. Gustin SM, Peck CC, Wilcox SL, Nash PG, Murray GM, Henderson LA. Different pain, different brain: thalamic anatomy in neuropathic and non-neuropathic chronic pain syndromes. *The Journal of neuroscience : the official journal of the Society for Neuroscience* 2011;**31**: 5956-64.<https://dx.doi.org/10.1523/JNEUROSCI.5980-10.2011>

75. Younger JW, Shen YF, Goddard G, Mackey SC. Chronic myofascial temporomandibular pain is associated with neural abnormalities in the trigeminal and limbic systems. *Pain* 2010;**149**: 222-8.<https://dx.doi.org/10.1016/j.pain.2010.01.006>

76. Domin M, Grimm NK, Klepzig K, Schmidt CO, Kordass B, Lotze M. Gray Matter Brain Alterations in Temporomandibular Disorder Tested in a Population Cohort and Three Clinical Samples. *The journal of pain* 2021;**22**: 739-47.<https://dx.doi.org/10.1016/j.jpain.2021.01.003>

77. Lim M, Nascimento TD, Kim DJ, Ellingrod VL, DaSilva AF. Aberrant Brain Signal Variability and COMT Genotype in Chronic TMD Patients. *Journal of dental research* 2021;**100**: 714-22.<https://dx.doi.org/10.1177/0022034521994089>

78. Mills EP, Akhter R, Di Pietro F, Murray GM, Peck CC, Macey PM, et al. Altered Brainstem Pain Modulating Circuitry Functional Connectivity in Chronic Painful Temporomandibular Disorder. *The journal of pain* 2021;**22**: 219-32.<https://dx.doi.org/10.1016/j.jpain.2020.08.002>

79. Kucyi A, Moayedi M, Weissman-Fogel I, Goldberg MB, Freeman BV, Tenenbaum HC, et al. Enhanced medial prefrontal-default mode network functional connectivity in chronic pain and its association with pain rumination. *The Journal of neuroscience : the official journal of the Society for Neuroscience* 2014;**34**: 3969-75.<https://dx.doi.org/10.1523/JNEUROSCI.5055-13.2014>

80. Chen X-F, He P, Xu K-H, Jin Y-H, Chen Y, Wang B, et al. Disrupted Spontaneous Neural Activity and Its Interaction With Pain and Emotion in Temporomandibular Disorders. *Frontiers in neuroscience* 2022;**16**: 941244.<https://dx.doi.org/10.3389/fnins.2022.941244>

81. Jasim H, Ghafouri B, Gerdle B, Hedenberg-Magnusson B, Ernberg M. Altered levels of salivary and plasma pain related markers in temporomandibular disorders. *The journal of headache and pain* 2020;**21**: 105.<https://dx.doi.org/10.1186/s10194-020-01160-z>

82. Zhou Q, Li M, Fan Q, Chen F, Jiang G, Wang T, et al. Cerebral perfusion alterations in patients with trigeminal neuralgia as measured by pseudo-continuous arterial spin labeling. *Frontiers in neuroscience* 2022;**16**: 1065411.<https://dx.doi.org/10.3389/fnins.2022.1065411>

83. Li M, Yan J, Wen H, Lin J, Liang L, Li S, et al. Cortical thickness, gyrification and sulcal depth in trigeminal neuralgia. *Scientific reports* 2021;**11**: 16322.<https://dx.doi.org/10.1038/s41598-021-95811-z>

84. Liu J, Zhu J, Yuan F, Zhang X, Zhang Q. Abnormal brain white matter in patients with right trigeminal neuralgia: a diffusion tensor imaging study. *The journal of headache and pain* 2018;**19**: 46.<https://dx.doi.org/10.1186/s10194-018-0871-1>

85. Li X, Zhao H, Wang M, Li L, Wang X, Ma Z, et al. Thalamic segmentation based on diffusion tensor imaging in patients with trigeminal neuralgia. *Brain research* 2024;**1830**: 148832.<https://dx.doi.org/10.1016/j.brainres.2024.148832>

86. Xiong Y, Xiong X, Yang S, Tian T, Zhu W, Zhang Q. Bilateral brain microstructural alterations in patients with left-sided classic trigeminal neuralgia: a diffusion kurtosis imaging study. *Journal of neurosurgery* 2024;**140**: 498-506.<https://dx.doi.org/10.3171/2023.6.JNS232>

87. Filimonova E, Pashkov A, Moysak G, Martirosyan A, Zaitsev B, Rzaev J. Diffusion tensor imaging reveals distributed white matter abnormalities in primary trigeminal neuralgia: Tract-based spatial statistics study. *Clinical neurology and neurosurgery* 2024;**236**: 108080.<https://dx.doi.org/10.1016/j.clineuro.2023.108080>

88. DeSouza DD, Moayedi M, Chen DQ, Davis KD, Hodaie M. Sensorimotor and Pain Modulation Brain Abnormalities in Trigeminal Neuralgia: A Paroxysmal, Sensory-Triggered Neuropathic Pain. *PLOS ONE* 2013;**8**: e66340.10.1371/journal.pone.0066340

89. Sun B, Zhang C, Huang K, Bhetuwal A, Yang X, Jing C, et al. The white matter characteristic of the genu of corpus callosum coupled with pain intensity and negative emotion scores in patients with trigeminal neuralgia: a multivariate analysis. *Frontiers in neuroscience* 2024;**18**: 1381085.<https://dx.doi.org/10.3389/fnins.2024.1381085>

90. Mammadkhanli O, Niftaliyev S, Simsek O. Involvement of the cingulate cortex and insula in patients with trigeminal neuralgia: A clinical and volumetric study. *Clinical neurology and neurosurgery* 2024;**243**: 108394.<https://dx.doi.org/10.1016/j.clineuro.2024.108394>

91. Parise M, Kubo TTA, Doring TM, Tukamoto G, Vincent M, Gasparetto EL. Cuneus and fusiform cortices thickness is reduced in trigeminal neuralgia. *The journal of headache and pain* 2014;**15**: 17.<https://dx.doi.org/10.1186/1129-2377-15-17>

92. Li M, Yan J, Li S, Wang T, Zhan W, Wen H, et al. Reduced volume of gray matter in patients with trigeminal neuralgia. *Brain Imaging and Behavior* 2017;**11**: 486-92.10.1007/s11682-016-9529-2

93. Li R, Chang N, Liu Y, Zhang Y, Luo Y, Zhang T, et al. The Integrity of the Substructure of the Corpus Callosum in Patients With Right Classic Trigeminal Neuralgia. *The Journal of craniofacial surgery* 2021;**32**: 632-6.<https://dx.doi.org/10.1097/SCS.0000000000007082>

94. Albano L, Agosta F, Basaia S, Castellano A, Messina R, Parisi V, et al. Alterations of brain structural MRI are associated with outcome of surgical treatment in trigeminal neuralgia. *European journal of neurology* 2022;**29**: 305-17.<https://dx.doi.org/10.1111/ene.15105>

95. Wang Y, Yang Q, Cao D, Seminowicz D, Remeniuk B, Gao L, et al. Correlation between nerve atrophy, brain grey matter volume and pain severity in patients with primary trigeminal neuralgia. *Cephalalgia : an international journal of headache* 2019;**39**: 515-25.<https://dx.doi.org/10.1177/0333102418793643>

96. Obermann M, Rodriguez-Raecke R, Naegel S, Holle D, Mueller D, Yoon M-S, et al. Gray matter volume reduction reflects chronic pain in trigeminal neuralgia. *NeuroImage* 2013;**74**: 352-8.<https://dx.doi.org/10.1016/j.neuroimage.2013.02.029>

97. Ge X, Wang L, Pan L, Ye H, Zhu X, Fan S, et al. Alteration of the cortical morphology in classical trigeminal neuralgia: voxel-, deformation-, and surface-based analysis. *The journal of headache and pain* 2023;**24**: 17.<https://dx.doi.org/10.1186/s10194-023-01544-x>

98. Wilcox SL, Gustin SM, Macey PM, Peck CC, Murray GM, Henderson LA. Anatomical changes at the level of the primary synapse in neuropathic pain: evidence from the spinal trigeminal nucleus. *The Journal of neuroscience : the official journal of the Society for Neuroscience* 2015;**35**: 2508-15.<https://dx.doi.org/10.1523/JNEUROSCI.3756-14.2015>

99. Hayes DJ, Chen DQ, Zhong J, Lin A, Behan B, Walker M, et al. Affective Circuitry Alterations in Patients with Trigeminal Neuralgia. *Frontiers in neuroanatomy* 2017;**11**: 73.<https://dx.doi.org/10.3389/fnana.2017.00073>

100. Latypov TH, So MC, Hung PS-P, Tsai P, Walker MR, Tohyama S, et al. Brain imaging signatures of neuropathic facial pain derived by artificial intelligence. *Scientific reports* 2023;**13**: 10699.<https://dx.doi.org/10.1038/s41598-023-37034-y>

101. Zhang Y, Mao Z, Pan L, Ling Z, Liu X, Zhang J, et al. Dysregulation of Pain- and Emotion-Related Networks in Trigeminal Neuralgia. *Frontiers in human neuroscience* 2018;**12**: 107.<https://dx.doi.org/10.3389/fnhum.2018.00107>

102. Wang Y, Zhang Y, Zhang J, Wang J, Xu J, Li J, et al. Structural and functional abnormalities of the insular cortex in trigeminal neuralgia: a multimodal magnetic resonance imaging analysis. *Pain* 2018;**159**: 507-14.<https://dx.doi.org/10.1097/j.pain.0000000000001120>

103. Wang Y, Cao D-Y, Remeniuk B, Krimmel S, Seminowicz DA, Zhang M. Altered brain structure and function associated with sensory and affective components of classic trigeminal neuralgia. *Pain* 2017;**158**: 1561-70.<https://dx.doi.org/10.1097/j.pain.0000000000000951>

104. Wu M, Jiang X, Qiu J, Fu X, Niu C. Gray and white matter abnormalities in primary trigeminal neuralgia with and without neurovascular compression. *The journal of headache and pain* 2020;**21**: 136.<https://dx.doi.org/10.1186/s10194-020-01205-3>

105. Wang Y, Zhang X, Guan Q, Wan L, Yi Y, Liu C-F. Altered regional homogeneity of spontaneous brain activity in idiopathic trigeminal neuralgia. *Neuropsychiatric disease and treatment* 2015;**11**: 2659-66.<https://dx.doi.org/10.2147/NDT.S94877>

106. Yuan J, Cao S, Huang Y, Zhang Y, Xie P, Zhang Y, et al. Altered Spontaneous Brain Activity in Patients With Idiopathic Trigeminal Neuralgia: A Resting-state Functional MRI Study. *The Clinical journal of pain* 2018;**34**: 600-9.<https://dx.doi.org/10.1097/AJP.0000000000000578>

107. Tsai Y-H, Liang X, Yang J-T, Hsu L-M. Modular organization of brain resting state networks in patients with classical trigeminal neuralgia. *NeuroImage Clinical* 2019;**24**: 102027.<https://dx.doi.org/10.1016/j.nicl.2019.102027>

108. Zhang Y, Mao Z, Pan L, Ling Z, Liu X, Zhang J, et al. Frequency-specific alterations in cortical rhythms and functional connectivity in trigeminal neuralgia. *Brain imaging and behavior* 2019;**13**: 1497-509.<https://dx.doi.org/10.1007/s11682-019-00105-8>

109. Zhang P, Jiang Y, Liu G, Han J, Wang J, Ma L, et al. Altered brain functional network dynamics in classic trigeminal neuralgia: a resting-state functional magnetic resonance imaging study. *The journal of headache and pain* 2021;**22**: 147.<https://dx.doi.org/10.1186/s10194-021-01354-z>

110. Xu H, Seminowicz DA, Krimmel SR, Zhang M, Gao L, Wang Y. Altered Structural and Functional Connectivity of Salience Network in Patients with Classic Trigeminal Neuralgia. *The journal of pain* 2022;**23**: 1389-99.<https://dx.doi.org/10.1016/j.jpain.2022.02.012>

111. Puri BK, Agour M, Gunatilake KD, Fernando KA, Gurusinghe AI, Treasaden IH. Reduction in left supplementary motor area grey matter in adult female fibromyalgia sufferers with marked fatigue and without affective disorder: a pilot controlled 3-T magnetic resonance imaging voxel-based morphometry study. *The Journal of international medical research* 2010;**38**: 1468-72.<https://dx.doi.org/10.1177/147323001003800429>

112. Feraco P, Nigro S, Passamonti L, Grecucci A, Caligiuri ME, Gagliardo C, et al. Neurochemical Correlates of Brain Atrophy in Fibromyalgia Syndrome: A Magnetic Resonance Spectroscopy and Cortical Thickness Study. *Brain sciences* 2020;**10**.<https://dx.doi.org/10.3390/brainsci10060395>

113. Schmidt-Wilcke T, Luerding R, Weigand T, Jurgens T, Schuierer G, Leinisch E, et al. Striatal grey matter increase in patients suffering from fibromyalgia--a voxel-based morphometry study. *Pain* 2007;**132 Suppl 1**: S109-S16.<https://dx.doi.org/10.1016/j.pain.2007.05.010>

114. Leon-Llamas JL, Villafaina S, Murillo-Garcia A, Gusi N. Impact of Fibromyalgia in the Hippocampal Subfields Volumes of Women-An MRI Study. *International journal of environmental research and public health* 2021;**18**.<https://dx.doi.org/10.3390/ijerph18041549>

115. Niddam DM, Lee SH, Su YT, Chan RC. Brain structural changes in patients with chronic myofascial pain. *European journal of pain (London, England)* 2017;**21**: 148-58.<https://dx.doi.org/10.1002/ejp.911>

116. Mosch B, Hagena V, Herpertz S, Diers M. Brain morphometric changes in fibromyalgia and the impact of psychometric and clinical factors: a volumetric and diffusion-tensor imaging study. *Arthritis research & therapy* 2023;**25**: 81.<https://dx.doi.org/10.1186/s13075-023-03064-0>

117. Lutz J, Jager L, de Quervain D, Krauseneck T, Padberg F, Wichnalek M, et al. White and gray matter abnormalities in the brain of patients with fibromyalgia: a diffusion-tensor and volumetric imaging study. *Arthritis and rheumatism* 2008;**58**: 3960-9.<https://dx.doi.org/10.1002/art.24070>

118. Ceko M, Bushnell MC, Fitzcharles M-A, Schweinhardt P. Fibromyalgia interacts with age to change the brain. *NeuroImage Clinical* 2013;**3**: 249-60.<https://dx.doi.org/10.1016/j.nicl.2013.08.015>

119. Hsu MC, Harris RE, Sundgren PC, Welsh RC, Fernandes CR, Clauw DJ, et al. No consistent difference in gray matter volume between individuals with fibromyalgia and age-matched healthy subjects when controlling for affective disorder. *Pain* 2009;**143**: 262-7.<https://dx.doi.org/10.1016/j.pain.2009.03.017>

120. Oliveria Neto PGd, Rego Ramos L, DosSantos MF. Behavioral Changes and Long-Term Cortical Thickness Alterations in Women with Fibromyalgia. *Journal of manipulative and physiological therapeutics* 2024.<https://dx.doi.org/10.1016/j.jmpt.2024.08.018>

121. Diaz-Piedra C, Guzman MA, Buela-Casal G, Catena A. The impact of fibromyalgia symptoms on brain morphometry. *Brain imaging and behavior* 2016;**10**: 1184-97.<https://dx.doi.org/10.1007/s11682-015-9485-2>

122. Nhu NT, Chen DY-T, Kang J-H. Functional Connectivity and Structural Signatures of the Visual Cortical System in Fibromyalgia: A Magnetic Resonance Imaging Study. *The Journal of rheumatology* 2023;**50**: 1063-70.<https://dx.doi.org/10.3899/jrheum.2022-1309>

123. Izuno S, Yoshihara K, Hosoi M, Eto S, Hirabayashi N, Todani T, et al. Psychological characteristics associated with the brain volume of patients with fibromyalgia. *BioPsychoSocial medicine* 2023;**17**: 36.<https://dx.doi.org/10.1186/s13030-023-00293-2>

124. Sundgren PC, Petrou M, Harris RE, Fan X, Foerster B, Mehrotra N, et al. Diffusion-weighted and diffusion tensor imaging in fibromyalgia patients: a prospective study of whole brain diffusivity, apparent diffusion coefficient, and fraction anisotropy in different regions of the brain and correlation with symptom severity. *Academic radiology* 2007;**14**: 839-46.<https://dx.doi.org/10.1016/j.acra.2007.03.015>

125. Tu Y, Wang J, Xiong F, Gao F. Disrupted White Matter Microstructure in Patients With Fibromyalgia Owing Predominantly to Psychological Factors: A Diffusion Tensor Imaging Study. *Pain physician* 2022;**25**: E1305-E13

126. Cifre I, Sitges C, Fraiman D, Munoz MA, Balenzuela P, Gonzalez-Roldan A, et al. Disrupted functional connectivity of the pain network in fibromyalgia. *Psychosomatic medicine* 2012;**74**: 55-62.<https://dx.doi.org/10.1097/PSY.0b013e3182408f04>

127. Coulombe M-A, Lawrence KS, Moulin DE, Morley-Forster P, Shokouhi M, Nielson WR, et al. Lower Functional Connectivity of the Periaqueductal Gray Is Related to Negative Affect and Clinical Manifestations of Fibromyalgia. *Frontiers in neuroanatomy* 2017;**11**: 47.<https://dx.doi.org/10.3389/fnana.2017.00047>

128. Napadow V, LaCount L, Park K, As-Sanie S, Clauw DJ, Harris RE. Intrinsic brain connectivity in fibromyalgia is associated with chronic pain intensity. *Arthritis and rheumatism* 2010;**62**: 2545-55.<https://dx.doi.org/10.1002/art.27497>

129. Ichesco E, Schmidt-Wilcke T, Bhavsar R, Clauw DJ, Peltier SJ, Kim J, et al. Altered resting state connectivity of the insular cortex in individuals with fibromyalgia. *The journal of pain* 2014;**15**: 815-26.e1.<https://dx.doi.org/10.1016/j.jpain.2014.04.007>

130. Kim J-Y, Kim S-H, Seo J, Kim S-H, Han SW, Nam EJ, et al. Increased power spectral density in resting-state pain-related brain networks in fibromyalgia. *Pain* 2013;**154**: 1792-7.<https://dx.doi.org/10.1016/j.pain.2013.05.040>

131. Truini A, Tinelli E, Gerardi MC, Calistri V, Iannuccelli C, La Cesa S, et al. Abnormal resting state functional connectivity of the periaqueductal grey in patients with fibromyalgia. *Clinical and experimental rheumatology* 2016;**34**: S129-33

132. Kaplan CM, Schrepf A, Vatansever D, Larkin TE, Mawla I, Ichesco E, et al. Functional and neurochemical disruptions of brain hub topology in chronic pain. *Pain* 2019;**160**: 973-83.<https://dx.doi.org/10.1097/j.pain.0000000000001480>

133. Park SH, Baker AK, Krishna V, Mackey SC, Martucci KT. Altered resting-state functional connectivity within corticostriatal and subcortical-striatal circuits in chronic pain. *Scientific reports* 2022;**12**: 12683.<https://dx.doi.org/10.1038/s41598-022-16835-7>

134. Larkin TE, Kaplan CM, Schrepf A, Ichesco E, Mawla I, Harte SE, et al. Altered network architecture of functional brain communities in chronic nociplastic pain. *NeuroImage* 2021;**226**: 117504.<https://dx.doi.org/10.1016/j.neuroimage.2020.117504>

135. Alves RL, Zortea M, Serrano PV, Brugnera Tomedi R, Pereira de Almeida R, Torres ILS, et al. High-beta oscillations at EEG resting state and hyperconnectivity of pain circuitry in fibromyalgia: an exploratory cross-sectional study. *Frontiers in neuroscience* 2023;**17**: 1233979.<https://dx.doi.org/10.3389/fnins.2023.1233979>

136. Gonzalez-Villar AJ, Trinanes Y, Gomez-Perretta C, Carrillo-de-la-Pena MT. Patients with fibromyalgia show increased beta connectivity across distant networks and microstates alterations in resting-state electroencephalogram. *NeuroImage* 2020;**223**: 117266.<https://dx.doi.org/10.1016/j.neuroimage.2020.117266>

137. Fallon N, Chiu Y, Nurmikko T, Stancak A. Altered theta oscillations in resting EEG of fibromyalgia syndrome patients. *European journal of pain (London, England)* 2018;**22**: 49-57.<https://dx.doi.org/10.1002/ejp.1076>

138. Makowka S, Mory L-N, Mouthon M, Mancini C, Guggisberg AG, Chabwine JN. EEG Beta functional connectivity decrease in the left amygdala correlates with the affective pain in fibromyalgia: A pilot study. *PloS one* 2023;**18**: e0281986.<https://dx.doi.org/10.1371/journal.pone.0281986>

139. Gonzalez-Roldan AM, Cifre I, Sitges C, Montoya P. Altered Dynamic of EEG Oscillations in Fibromyalgia Patients at Rest. *Pain medicine (Malden, Mass)* 2016;**17**: 1058-68.<https://dx.doi.org/10.1093/pm/pnw023>

140. Villafaina S, Collado-Mateo D, Fuentes-Garcia JP, Cano-Plasencia R, Gusi N. Impact of Fibromyalgia on Alpha-2 EEG Power Spectrum in the Resting Condition: A Descriptive Correlational Study. *BioMed research international* 2019;**2019**: 7851047.<https://dx.doi.org/10.1155/2019/7851047>

141. Martin-Brufau R, Gomez MN, Sanchez-Sanchez-Rojas L, Nombela C. Fibromyalgia Detection Based on EEG Connectivity Patterns. *Journal of clinical medicine* 2021;**10**.<https://dx.doi.org/10.3390/jcm10153277>

142. Vanneste S, Ost J, Van Havenbergh T, De Ridder D. Resting state electrical brain activity and connectivity in fibromyalgia. *PloS one* 2017;**12**: e0178516.<https://dx.doi.org/10.1371/journal.pone.0178516>

143. Gomez-Beldarrain M, Oroz I, Zapirain BG, Ruanova BF, Fernandez YG, Cabrera A, et al. Right fronto-insular white matter tracts link cognitive reserve and pain in migraine patients. *The journal of headache and pain* 2015;**17**: 4.<https://dx.doi.org/10.1186/s10194-016-0593-1>

144. Tantik Pak A, Nacar Dogan S, Sengul Y. Structural integrity of corpus callosum in patients with migraine: a diffusion tensor imaging study. *Acta neurologica Belgica* 2023;**123**: 385-90.<https://dx.doi.org/10.1007/s13760-021-01863-3>

145. Coppola G, Di Renzo A, Tinelli E, Petolicchio B, Di Lorenzo C, Parisi V, et al. Patients with chronic migraine without history of medication overuse are characterized by a peculiar white matter fiber bundle profile. *The journal of headache and pain* 2020;**21**: 92.<https://dx.doi.org/10.1186/s10194-020-01159-6>

146. Naegel S, Holle D, Desmarattes N, Theysohn N, Diener H-C, Katsarava Z, et al. Cortical plasticity in episodic and chronic cluster headache. *NeuroImage Clinical* 2014;**6**: 415-23.<https://dx.doi.org/10.1016/j.nicl.2014.10.003>

147. Hubbard CS, Khan SA, Keaser ML, Mathur VA, Goyal M, Seminowicz DA. Altered Brain Structure and Function Correlate with Disease Severity and Pain Catastrophizing in Migraine Patients. *eNeuro* 2014;**1**: e20.14.<https://dx.doi.org/10.1523/ENEURO.0006-14.2014>

148. Schmidt-Wilcke T, Ganssbauer S, Neuner T, Bogdahn U, May A. Subtle grey matter changes between migraine patients and healthy controls. *Cephalalgia : an international journal of headache* 2008;**28**: 1-4.<https://dx.doi.org/10.1111/j.1468-2982.2007.01428.x>

149. Mammadkhanli O, Kehaya S, Solak S, Yagmurlu K. Insular cortex involvement in migraine patients with chronic pain: A volumetric radiological and clinical study. *Journal of clinical neuroscience : official journal of the Neurosurgical Society of Australasia* 2024;**123**: 157-61.<https://dx.doi.org/10.1016/j.jocn.2024.03.034>

150. Schmidt-Wilcke T, Leinisch E, Straube A, Kampfe N, Draganski B, Diener HC, et al. Gray matter decrease in patients with chronic tension type headache. *Neurology* 2005;**65**: 1483-6.<https://dx.doi.org/10.1212/01.wnl.0000183067.94400.80>

151. Neeb L, Bastian K, Villringer K, Israel H, Reuter U, Fiebach JB. Structural Gray Matter Alterations in Chronic Migraine: Implications for a Progressive Disease? *Headache* 2017;**57**: 400-16.<https://dx.doi.org/10.1111/head.13012>

152. Ferraro S, Medina JP, Nigri A, Giani L, Demichelis G, Pinardi C, et al. Mesocorticolimbic system abnormalities in chronic cluster headache patients: A neural signature? *Cephalalgia : an international journal of headache* 2022;**42**: 1039-49.<https://dx.doi.org/10.1177/03331024221092416>

153. Chen B, He Y, Xia L, Guo L-L, Zheng J-L. Cortical plasticity between the pain and pain-free phases in patients with episodic tension-type headache. *The journal of headache and pain* 2016;**17**: 105.<https://dx.doi.org/10.1186/s10194-016-0698-6>

154. De Ridder D, Adhia D, Vanneste S. The brain's duck test in phantom percepts: Multisensory congruence in neuropathic pain and tinnitus. *Brain research* 2024;**1844**: 149137.<https://dx.doi.org/10.1016/j.brainres.2024.149137>

155. Ferraro S, Nigri A, Bruzzone MG, Brivio L, Proietti Cecchini A, Verri M, et al. Defective functional connectivity between posterior hypothalamus and regions of the diencephalic-mesencephalic junction in chronic cluster headache. *Cephalalgia : an international journal of headache* 2018;**38**: 1910-8.<https://dx.doi.org/10.1177/0333102418761048>

156. Liu K, Cheng J, Cao Y, Chen K, Li Y, Zhang X, et al. Abnormally Increased Effective Connectivity of the Periaqueductal Gray in Migraine Without Aura Patients. *The Clinical journal of pain* 2023;**39**: 175-9.<https://dx.doi.org/10.1097/AJP.0000000000001099>

157. Zhang D, Huang X, Su W, Chen Y, Wang P, Mao C, et al. Altered lateral geniculate nucleus functional connectivity in migraine without aura: a resting-state functional MRI study. *The journal of headache and pain* 2020;**21**: 17.<https://dx.doi.org/10.1186/s10194-020-01086-6>

158. Gecse K, Dobos D, Aranyi CS, Galambos A, Baksa D, Kocsel N, et al. Association of plasma tryptophan concentration with periaqueductal gray matter functional connectivity in migraine patients. *Scientific reports* 2022;**12**: 739.<https://dx.doi.org/10.1038/s41598-021-04647-0>

159. Liu J, Zhao L, Lei F, Zhang Y, Yuan K, Gong Q, et al. Disrupted resting-state functional connectivity and its changing trend in migraine suffers. *Human brain mapping* 2015;**36**: 1892-907.<https://dx.doi.org/10.1002/hbm.22744>

160. Zhang L, Yu W, Zhang Z, Xu M, Cui F, Song W, et al. Altered brain activity and functional connectivity in migraine without aura during and outside attack. *Neurological research* 2023;**45**: 603-9.<https://dx.doi.org/10.1080/01616412.2023.2170938>

161. Huang X, Zhang D, Chen Y, Wang P, Mao C, Miao Z, et al. Altered functional connectivity of the red nucleus and substantia nigra in migraine without aura. *The journal of headache and pain* 2019;**20**: 104.<https://dx.doi.org/10.1186/s10194-019-1058-0>

162. Schwedt TJ, Larson-Prior L, Coalson RS, Nolan T, Mar S, Ances BM, et al. Allodynia and descending pain modulation in migraine: a resting state functional connectivity analysis. *Pain medicine (Malden, Mass)* 2014;**15**: 154-65.<https://dx.doi.org/10.1111/pme.12267>

163. Qin Z, Su J, He X-W, Ban S, Zhu Q, Cui Y, et al. Disrupted functional connectivity between sub-regions in the sensorimotor areas and cortex in migraine without aura. *The journal of headache and pain* 2020;**21**: 47.<https://dx.doi.org/10.1186/s10194-020-01118-1>

164. Gao Q, Xu F, Jiang C, Chen Z, Chen H, Liao H, et al. Decreased functional connectivity density in pain-related brain regions of female migraine patients without aura. *Brain research* 2016;**1632**: 73-81.<https://dx.doi.org/10.1016/j.brainres.2015.12.007>

165. Maki-Marttunen V, Kies DA, Pijpers JA, Louter MA, van der Wee NJ, Rombouts SARB, et al. Functional connectivity of the visual cortex in chronic migraine before and after medication withdrawal therapy. *NeuroImage Clinical* 2023;**40**: 103543.<https://dx.doi.org/10.1016/j.nicl.2023.103543>

166. Li Z, Liu M, Lan L, Zeng F, Makris N, Liang Y, et al. Altered periaqueductal gray resting state functional connectivity in migraine and the modulation effect of treatment. *Scientific reports* 2016;**6**: 20298.<https://dx.doi.org/10.1038/srep20298>

167. Chen Z, Chen X, Liu M, Liu S, Shu S, Ma L, et al. Altered functional connectivity of the marginal division in migraine: a resting-state fMRI study. *The journal of headache and pain* 2016;**17**: 89.<https://dx.doi.org/10.1186/s10194-016-0682-1>

168. Feng S, Li B, Li G, Hua X, Zhu B, Li X, et al. Abnormal Spatial Patterns of Intrinsic Brain Activity in Osteonecrosis of the Femoral Head: A Resting-State Functional Magnetic Resonance Imaging Study. *Frontiers in human neuroscience* 2020;**14**: 551470.<https://dx.doi.org/10.3389/fnhum.2020.551470>

169. Barroso J, Vigotsky AD, Branco P, Reis AM, Schnitzer TJ, Galhardo V, et al. Brain gray matter abnormalities in osteoarthritis pain: a cross-sectional evaluation. *Pain* 2020;**161**: 2167-78.<https://dx.doi.org/10.1097/j.pain.0000000000001904>

170. Gwilym SE, Filippini N, Douaud G, Carr AJ, Tracey I. Thalamic atrophy associated with painful osteoarthritis of the hip is reversible after arthroplasty: a longitudinal voxel-based morphometric study. *Arthritis and rheumatism* 2010;**62**: 2930-40.<https://dx.doi.org/10.1002/art.27585>

171. Johnson AJ, Cole J, Fillingim RB, Cruz-Almeida Y. Persistent Non-pharmacological Pain Management and Brain-Predicted Age Differences in Middle-Aged and Older Adults With Chronic Knee Pain. *Frontiers in pain research (Lausanne, Switzerland)* 2022;**3**: 868546.<https://dx.doi.org/10.3389/fpain.2022.868546>

172. Johnson AJ, Buchanan T, Laffitte Nodarse C, Valdes Hernandez PA, Huo Z, Cole JH, et al. Cross-Sectional Brain-Predicted Age Differences in Community-Dwelling Middle-Aged and Older Adults with High Impact Knee Pain. *Journal of pain research* 2022;**15**: 3575-87.<https://dx.doi.org/10.2147/JPR.S384229>

173. Valdes-Hernandez PA, Laffitte Nodarse C, Johnson AJ, Montesino-Goicolea S, Bashyam V, Davatzikos C, et al. Brain-predicted age difference estimated using DeepBrainNet is significantly associated with pain and function-a multi-institutional and multiscanner study. *Pain* 2023;**164**: 2822-38.<https://dx.doi.org/10.1097/j.pain.0000000000002984>

174. Iwabuchi SJ, Xing Y, Cottam WJ, Drabek MM, Tadjibaev A, Fernandes GS, et al. Brain perfusion patterns are altered in chronic knee pain: a spatial covariance analysis of arterial spin labelling MRI. *Pain* 2020;**161**: 1255-63.<https://dx.doi.org/10.1097/j.pain.0000000000001829>

175. Cottam WJ, Condon L, Alshuft H, Reckziegel D, Auer DP. Associations of limbic-affective brain activity and severity of ongoing chronic arthritis pain are explained by trait anxiety. *NeuroImage Clinical* 2016;**12**: 269-76.<https://dx.doi.org/10.1016/j.nicl.2016.06.022>

176. Sanchis-Alfonso V, Beser-Robles M, Ten-Esteve A, Ramirez-Fuentes C, Alberich-Bayarri A, Espert R, et al. Brain network functional connectivity changes in patients with anterior knee pain: a resting-state fMRI exploratory study. *European radiology experimental* 2023;**7**: 60.<https://dx.doi.org/10.1186/s41747-023-00378-1>

177. Iwabuchi SJ, Drabek MM, Cottam WJ, Tadjibaev A, Mohammadi-Nejad A-R, Sotiropoulos S, et al. Medio-dorsal thalamic dysconnectivity in chronic knee pain: A possible mechanism for negative affect and pain comorbidity. *The European journal of neuroscience* 2023;**57**: 373-87.<https://dx.doi.org/10.1111/ejn.15880>

178. Mao CP, Bai ZL, Zhang XN, Zhang QJ, Zhang L. Abnormal Subcortical Brain Morphology in Patients with Knee Osteoarthritis: A Cross-sectional Study. *Frontiers in Aging Neuroscience* 2016;**8**.10.3389/fnagi.2016.00003

179. Liao X, Mao C, Wang Y, Zhang Q, Cao D, Seminowicz DA, et al. Brain gray matter alterations in Chinese patients with chronic knee osteoarthritis pain based on voxel-based morphometry. *Medicine* 2018;**97**: e0145.<https://dx.doi.org/10.1097/MD.0000000000010145>

180. Zeng P, Zhao B, Li M, Wang Y, Cai G, Chen R, et al. The volumes of amygdala subregions and peripheral programmed cell death protein-1 levels are associated with cognitive decline in individuals with knee osteoarthritis. *Brain and behavior* 2024;**14**: e70042.<https://dx.doi.org/10.1002/brb3.70042>

181. Kang B-X, Ma J, Shen J, Xu H, Wang H-Q, Zhao C, et al. Altered brain activity in end-stage knee osteoarthritis revealed by resting-state functional magnetic resonance imaging. *Brain and behavior* 2022;**12**: e2479.<https://dx.doi.org/10.1002/brb3.2479>

182. Ushio K, Nakanishi K, Yoshino A, Takamura M, Akiyama Y, Shimada N, et al. Changed resting-state connectivity of anterior insular cortex affects subjective pain reduction after knee arthroplasty: A longitudinal study. *Brain research bulletin* 2024;**217**: 111073.<https://dx.doi.org/10.1016/j.brainresbull.2024.111073>

183. Lieberman G, Shpaner M, Watts R, Andrews T, Filippi CG, Davis M, et al. White matter involvement in chronic musculoskeletal pain. *The journal of pain* 2014;**15**: 1110-9.<https://dx.doi.org/10.1016/j.jpain.2014.08.002>

184. Van Riper SM, Alexander AL, Koltyn KF, Stegner AJ, Ellingson LD, Destiche DJ, et al. Cerebral white matter structure is disrupted in Gulf War Veterans with chronic musculoskeletal pain. *Pain* 2017;**158**: 2364-75.<https://dx.doi.org/10.1097/j.pain.0000000000001038>

185. Ninneman JV, Gretzon NP, Stegner AJ, Lindheimer JB, Falvo MJ, Wylie GR, et al. Pain, But Not Physical Activity, Is Associated with Gray Matter Volume Differences in Gulf War Veterans with Chronic Pain. *The Journal of neuroscience : the official journal of the Society for Neuroscience* 2022;**42**: 5605-16.<https://dx.doi.org/10.1523/JNEUROSCI.2394-21.2022>

186. Bishop JH, Shpaner M, Kubicki A, Clements S, Watts R, Naylor MR. Structural network differences in chronic muskuloskeletal pain: Beyond fractional anisotropy. *NeuroImage* 2018;**182**: 441-55.<https://dx.doi.org/10.1016/j.neuroimage.2017.12.021>

187. Duke Han S, Buchman AS, Arfanakis K, Fleischman DA, Bennett DA. Functional connectivity networks associated with chronic musculoskeletal pain in old age. *International journal of geriatric psychiatry* 2013;**28**: 858-67.<https://dx.doi.org/10.1002/gps.3898>

188. Conboy V, Edwards C, Ainsworth R, Natusch D, Burcham C, Danisment B, et al. Chronic musculoskeletal impairment is associated with alterations in brain regions responsible for the production and perception of movement. *The Journal of physiology* 2021;**599**: 2255-72.<https://dx.doi.org/10.1113/JP281273>

189. Chatterjee I, Baumgartner L, Cho M. Detection of brain regions responsible for chronic pain in osteoarthritis: an fMRI-based neuroimaging study using deep learning. *Frontiers in neurology* 2023;**14**: 1195923.<https://dx.doi.org/10.3389/fneur.2023.1195923>

190. Song G, Zhang Y, Qin B, Zeng J, Zhang T, Xie P. Altered Neurovascular Coupling in Patients with Chronic Myofascial Pain. *Pain physician* 2021;**24**: E601-E10

191. Wang Y, Li Q, Xue Xa, Xu X, Tao W, Liu S, et al. Neuroplasticity of pain processing and motor control in CAI patients: A UK Biobank study with clinical validation. *Frontiers in molecular neuroscience* 2023;**16**: 1096930.<https://dx.doi.org/10.3389/fnmol.2023.1096930>

192. Meneses FM, Queiros FC, Montoya P, Miranda JGV, Dubois-Mendes SM, Sa KN, et al. Patients with Rheumatoid Arthritis and Chronic Pain Display Enhanced Alpha Power Density at Rest. *Frontiers in human neuroscience* 2016;**10**: 395.<https://dx.doi.org/10.3389/fnhum.2016.00395>

193. Niddam DM, Lee S-H, Su Y-T, Chan R-C. Altered cortical morphology in patients with chronic shoulder pain. *Neuroscience letters* 2019;**712**: 134515.<https://dx.doi.org/10.1016/j.neulet.2019.134515>

194. Li J, Zhao R, Wang C, Guo X, Song J, Chu X. Abnormal preoperative fMRI signal variability in the pain ascending pathway is associated with the postoperative axial pain intensity in degenerative cervical myelopathy patients. *The spine journal : official journal of the North American Spine Society* 2024;**24**: 78-86.<https://dx.doi.org/10.1016/j.spinee.2023.09.003>

195. Li J, Zhao R, Wang C, Song J, Guo X, Ge Y, et al. Structural and functional abnormalities in the medial prefrontal cortex were associated with pain and depressive symptoms in patients with adhesive capsulitis. *Pain reports* 2024;**9**: e1139.<https://dx.doi.org/10.1097/PR9.0000000000001139>

196. Wei X, Shi G, Tu J, Zhou H, Duan Y, Lee CK, et al. Structural and Functional Asymmetry in Precentral and Postcentral Gyrus in Patients With Unilateral Chronic Shoulder Pain. *Front Neurol* 2022;**13**: 792695.10.3389/fneur.2022.792695

197. Li J-L, Yan C-Q, Wang X, Zhang S, Zhang N, Hu S-Q, et al. Brain Functional Alternations of the Pain-related Emotional and Cognitive Regions in Patients with Chronic Shoulder Pain. *Journal of pain research* 2020;**13**: 575-83.<https://dx.doi.org/10.2147/JPR.S220370>

198. Russell MD, Barrick TR, Howe FA, Sofat N. Reduced anterior cingulate grey matter volume in painful hand osteoarthritis. *Rheumatology international* 2018;**38**: 1429-35.<https://dx.doi.org/10.1007/s00296-018-4085-2>

199. Hung PS-P, Zhang JY, Noorani A, Walker MR, Huang M, Zhang JW, et al. Differential expression of a brain aging biomarker across discrete chronic pain disorders. *Pain* 2022;**163**: 1468-78.<https://dx.doi.org/10.1097/j.pain.0000000000002613>

200. Cruz-Almeida Y, Fillingim RB, Riley JL, 3rd, Woods AJ, Porges E, Cohen R, et al. Chronic pain is associated with a brain aging biomarker in community-dwelling older adults. *Pain* 2019;**160**: 1119-30.<https://dx.doi.org/10.1097/j.pain.0000000000001491>

201. Malinen S, Vartiainen N, Hlushchuk Y, Koskinen M, Ramkumar P, Forss N, et al. Aberrant temporal and spatial brain activity during rest in patients with chronic pain. *Proceedings of the National Academy of Sciences of the United States of America* 2010;**107**: 6493-7.<https://dx.doi.org/10.1073/pnas.1001504107>

202. Huang L, Kutch JJ, Ellingson BM, Martucci KT, Harris RE, Clauw DJ, et al. Brain white matter changes associated with urological chronic pelvic pain syndrome: multisite neuroimaging from a MAPP case-control study. *Pain* 2016;**157**: 2782-91.<https://dx.doi.org/10.1097/j.pain.0000000000000703>

203. Woodworth DC, Holly LT, Mayer EA, Salamon N, Ellingson BM. Alterations in Cortical Thickness and Subcortical Volume are Associated With Neurological Symptoms and Neck Pain in Patients With Cervical Spondylosis. *Neurosurgery* 2019;**84**: 588-98.<https://dx.doi.org/10.1093/neuros/nyy066>

204. Gupta A, Woodworth DC, Ellingson BM, Rapkin AJ, Naliboff B, Kilpatrick LA, et al. Disease-Related Microstructural Differences in the Brain in Women With Provoked Vestibulodynia. *The journal of pain* 2018;**19**: 528.e1-.e15.<https://dx.doi.org/10.1016/j.jpain.2017.12.269>

205. Bell TR, Franz CE, Eyler LT, Fennema-Notestine C, Puckett OK, Dorros SM, et al. Probable chronic pain, brain structure, and Alzheimer's plasma biomarkers in older men. *The journal of pain* 2024;**25**: 104463.<https://dx.doi.org/10.1016/j.jpain.2024.01.006>

206. Baliki MN, Schnitzer TJ, Bauer WR, Apkarian AV. Brain morphological signatures for chronic pain. *PloS one* 2011;**6**: e26010.<https://dx.doi.org/10.1371/journal.pone.0026010>

207. Ruscheweyh R, Deppe M, Lohmann H, Stehling C, Floel A, Ringelstein BE, et al. Pain is associated with regional grey matter reduction in the general population. *Pain* 2011;**152**: 904-11.<https://dx.doi.org/10.1016/j.pain.2011.01.013>

208. Sundermann B, Dehghan Nayyeri M, Pfleiderer B, Stahlberg K, Junke L, Baie L, et al. Subtle changes of gray matter volume in fibromyalgia reflect chronic musculoskeletal pain rather than disease-specific effects. *The European journal of neuroscience* 2019;**50**: 3958-67.<https://dx.doi.org/10.1111/ejn.14558>

209. Ezzati A, Zammit AR, Lipton ML, Lipton RB. The relationship between hippocampal volume, chronic pain, and depressive symptoms in older adults. *Psychiatry research Neuroimaging* 2019;**289**: 10-2.<https://dx.doi.org/10.1016/j.pscychresns.2019.05.003>

210. Bhatt RR, Haddad E, Zhu AH, Thompson PM, Gupta A, Mayer EA, et al. Mapping Brain Structure Variability in Chronic Pain: The Role of Widespreadness and Pain Type and Its Mediating Relationship With Suicide Attempt. *Biological psychiatry* 2024;**95**: 473-81.<https://dx.doi.org/10.1016/j.biopsych.2023.07.016>

211. Neumann N, Domin M, Schmidt C-O, Lotze M. Chronic pain is associated with less grey matter volume in the anterior cingulum, anterior and posterior insula and hippocampus across three different chronic pain conditions. *European journal of pain (London, England)* 2023;**27**: 1239-48.<https://dx.doi.org/10.1002/ejp.2153>

212. Yang Q, Wang Z, Yang L, Xu Y, Chen LM. Cortical thickness and functional connectivity abnormality in chronic headache and low back pain patients. *Human brain mapping* 2017;**38**: 1815-32.<https://dx.doi.org/10.1002/hbm.23484>

213. Ikeda E, Li T, Kobinata H, Zhang S, Kurata J. Anterior insular volume decrease is associated with dysfunction of the reward system in patients with chronic pain. *European journal of pain (London, England)* 2018;**22**: 1170-9.<https://dx.doi.org/10.1002/ejp.1205>

214. Kutch JJ, Ichesco E, Hampson JP, Labus JS, Farmer MA, Martucci KT, et al. Brain signature and functional impact of centralized pain: a multidisciplinary approach to the study of chronic pelvic pain (MAPP) network study. *Pain* 2017;**158**: 1979-91.<https://dx.doi.org/10.1097/j.pain.0000000000001001>

215. McConnell PA, Garland EL, Zubieta J-K, Newman-Norlund R, Powers S, Froeliger B. Impaired frontostriatal functional connectivity among chronic opioid using pain patients is associated with dysregulated affect. *Addiction biology* 2020;**25**: e12743.<https://dx.doi.org/10.1111/adb.12743>

216. Lam J, Martensson J, Westergren H, Svensson P, Sundgren PC, Alstergren P. Structural MRI findings in the brain related to pain distribution in chronic overlapping pain conditions: An explorative case-control study in females with fibromyalgia, temporomandibular disorder-related chronic pain and pain-free controls. *Journal of oral rehabilitation* 2024;**51**: 2415-26.<https://dx.doi.org/10.1111/joor.13842>

217. Davis DA, Ghantous ME, Farmer MA, Baria AT, Apkarian AV. Identifying brain nociceptive information transmission in patients with chronic somatic pain. *Pain reports* 2016;**1**: e575.<https://dx.doi.org/10.1097/PR9.0000000000000575>

218. Pujol J, Blanco-Hinojo L, Doreste A, Ojeda F, Martinez-Vilavella G, Perez-Sola V, et al. Distinctive alterations in the functional anatomy of the cerebral cortex in pain-sensitized osteoarthritis and fibromyalgia patients. *Arthritis research & therapy* 2022;**24**: 252.<https://dx.doi.org/10.1186/s13075-022-02942-3>

219. Gupta A, Rapkin AJ, Gill Z, Kilpatrick L, Fling C, Stains J, et al. Disease-related differences in resting-state networks: a comparison between localized provoked vulvodynia, irritable bowel syndrome, and healthy control subjects. *Pain* 2015;**156**: 809-19.<https://dx.doi.org/10.1097/01.j.pain.0000461289.65571.54>

220. Delgado-Gallen S, Soler MD, Cabello-Toscano M, Abellaneda-Perez K, Solana-Sanchez J, Espana-Irla G, et al. Brain system segregation and pain catastrophizing in chronic pain progression. *Frontiers in neuroscience* 2023;**17**: 1148176.<https://dx.doi.org/10.3389/fnins.2023.1148176>

221. Hadjikhani N, Ward N, Boshyan J, Napadow V, Maeda Y, Truini A, et al. The missing link: enhanced functional connectivity between amygdala and visceroceptive cortex in migraine. *Cephalalgia : an international journal of headache* 2013;**33**: 1264-8.<https://dx.doi.org/10.1177/0333102413490344>

222. Michels L, Christidi F, Steiger VR, Sandor PS, Gantenbein AR, Landmann G, et al. Pain modulation is affected differently in medication-overuse headache and chronic myofascial pain - A multimodal MRI study. *Cephalalgia : an international journal of headache* 2017;**37**: 764-79.<https://dx.doi.org/10.1177/0333102416652625>

223. Schwenkreis P, Scherens A, Ronnau A-K, Hoffken O, Tegenthoff M, Maier C. Cortical disinhibition occurs in chronic neuropathic, but not in chronic nociceptive pain. *BMC neuroscience* 2010;**11**: 73.<https://dx.doi.org/10.1186/1471-2202-11-73>

224. Klug S, Anderer P, Saletu-Zyhlarz G, Freidl M, Saletu B, Prause W, et al. Dysfunctional pain modulation in somatoform pain disorder patients. *European archives of psychiatry and clinical neuroscience* 2011;**261**: 267-75.<https://dx.doi.org/10.1007/s00406-010-0148-4>

225. Schuurman BB, Vossen CJ, van Amelsvoort TAMJ, Lousberg RL. Does baseline EEG activity differ in the transition to or from a chronic pain state? A longitudinal study. *Pain practice : the official journal of World Institute of Pain* 2023;**23**: 479-92.<https://dx.doi.org/10.1111/papr.13204>

226. Ta Dinh S, Nickel MM, Tiemann L, May ES, Heitmann H, Hohn VD, et al. Brain dysfunction in chronic pain patients assessed by resting-state electroencephalography. *Pain* 2019;**160**: 2751-65.<https://dx.doi.org/10.1097/j.pain.0000000000001666>

227. Dimmek DJ, Korallus C, Buyny S, Christoph G, Lichtinghagen R, Jacobs R, et al. Brain-Derived Neurotrophic Factor and Immune Cells in Osteoarthritis, Chronic Low Back Pain, and Chronic Widespread Pain Patients: Association with Anxiety and Depression. *Medicina (Kaunas, Lithuania)* 2021;**57**.<https://dx.doi.org/10.3390/medicina57040327>

228. Sarchielli P, Mancini ML, Floridi A, Coppola F, Rossi C, Nardi K, et al. Increased levels of neurotrophins are not specific for chronic migraine: evidence from primary fibromyalgia syndrome. *The journal of pain* 2007;**8**: 737-45.<https://dx.doi.org/10.1016/j.jpain.2007.05.002>

229. Stefani LC, Leite FM, da Graca L Tarrago M, Zanette SA, de Souza A, Castro SM, et al. BDNF and serum S100B levels according the spectrum of structural pathology in chronic pain patients. *Neuroscience letters* 2019;**706**: 105-9.<https://dx.doi.org/10.1016/j.neulet.2019.05.021>

230. Deitos A, Dussan-Sarria JA, Souza Ad, Medeiros L, Tarrago MdG, Sehn F, et al. Clinical Value of Serum Neuroplasticity Mediators in Identifying the Central Sensitivity Syndrome in Patients With Chronic Pain With and Without Structural Pathology. *The Clinical journal of pain* 2015;**31**: 959-67.<https://dx.doi.org/10.1097/AJP.0000000000000194>

231. Caumo W, Antunes LC, Elkfury JL, Herbstrith EG, Busanello Sipmann R, Souza A, et al. The Central Sensitization Inventory validated and adapted for a Brazilian population: psychometric properties and its relationship with brain-derived neurotrophic factor. *Journal of pain research* 2017;**10**: 2109-22.<https://dx.doi.org/10.2147/JPR.S131479>

232. de Zoete RMJ, Stanwell P, Weber KA, Snodgrass SJ. Differences in Structural Brain Characteristics Between Individuals with Chronic Nonspecific Neck Pain and Asymptomatic Controls: A Case-Control Study. *Journal of pain research* 2022;**15**: 521-31.<https://dx.doi.org/10.2147/JPR.S345365>

233. Murillo C, Lopez-Sola M, Cagnie B, Sunol M, Smeets RJEM, Coppieters I, et al. Gray Matter Adaptations to Chronic Pain in People with Whiplash-Associated Disorders are Partially Reversed After Treatment: A Voxel-based Morphometry Study. *The journal of pain* 2024;**25**: 104471.<https://dx.doi.org/10.1016/j.jpain.2024.01.336>

234. De Pauw R, Coppieters I, Caeyenberghs K, Kregel J, Aerts H, Lenoir D, et al. Associations between brain morphology and motor performance in chronic neck pain: A whole-brain surface-based morphometry approach. *Human brain mapping* 2019;**40**: 4266-78.<https://dx.doi.org/10.1002/hbm.24700>

235. Coppieters I, De Pauw R, Caeyenberghs K, Danneels L, Kregel J, Pattyn A, et al. Decreased Regional Grey Matter Volume in Women with Chronic Whiplash-Associated Disorders: Relationships with Cognitive Deficits and Disturbed Pain Processing. *Pain physician* 2017;**20**: E1025-E51

236. Yang Q, Xu H, Zhang M, Wang Y, Li D. Volumetric and functional connectivity alterations in patients with chronic cervical spondylotic pain. *Neuroradiology* 2020;**62**: 995-1001.<https://dx.doi.org/10.1007/s00234-020-02413-z>

237. Coppieters I, De Pauw R, Caeyenberghs K, Lenoir D, DeBlaere K, Genbrugge E, et al. Differences in white matter structure and cortical thickness between patients with traumatic and idiopathic chronic neck pain: Associations with cognition and pain modulation? *Human brain mapping* 2018;**39**: 1721-42.<https://dx.doi.org/10.1002/hbm.23947>

238. Ihara N, Wakaizumi K, Nishimura D, Kato J, Yamada T, Suzuki T, et al. Aberrant resting-state functional connectivity of the dorsolateral prefrontal cortex to the anterior insula and its association with fear avoidance belief in chronic neck pain patients. *PloS one* 2019;**14**: e0221023.<https://dx.doi.org/10.1371/journal.pone.0221023>

239. Ma M, Zhang H, Liu R, Liu H, Yang X, Yin X, et al. Static and Dynamic Changes of Amplitude of Low-Frequency Fluctuations in Cervical Discogenic Pain. *Frontiers in neuroscience* 2020;**14**: 733.<https://dx.doi.org/10.3389/fnins.2020.00733>

240. Ni X, Zhang J, Sun M, Wang L, Xu T, Zeng Q, et al. Abnormal Dynamics of Functional Connectivity Density Associated With Chronic Neck Pain. *Frontiers in molecular neuroscience* 2022;**15**: 880228.<https://dx.doi.org/10.3389/fnmol.2022.880228>

241. Coppieters I, Cagnie B, De Pauw R, Meeus M, Timmers I. Enhanced amygdala-frontal operculum functional connectivity during rest in women with chronic neck pain: Associations with impaired conditioned pain modulation. *NeuroImage Clinical* 2021;**30**: 102638.<https://dx.doi.org/10.1016/j.nicl.2021.102638>

242. De Pauw R, Aerts H, Siugzdaite R, Meeus M, Coppieters I, Caeyenberghs K, et al. Hub disruption in patients with chronic neck pain: a graph analytical approach. *Pain* 2020;**161**: 729-41.<https://dx.doi.org/10.1097/j.pain.0000000000001762>

243. Yu C-X, Ji T-T, Song H, Li B, Han Q, Li L, et al. Abnormality of spontaneous brain activities in patients with chronic neck and shoulder pain: A resting-state fMRI study. *The Journal of international medical research* 2017;**45**: 182-92.<https://dx.doi.org/10.1177/0300060516679345>

244. Yu C-X, Li B, Xu Y-K, Ji T-T, Li L, Zhao C-J, et al. Altered functional connectivity of the periaqueductal gray in chronic neck and shoulder pain. *Neuroreport* 2017;**28**: 720-5.<https://dx.doi.org/10.1097/WNR.0000000000000819>

245. Bai L, Zhang L, Chen Y, Li Y, Ma D, Li W, et al. Middle cingulate cortex function contributes to response to non-steroidal anti-inflammatory drug in cervical spondylosis patients: a preliminary resting-state fMRI study. *Neuroradiology* 2022;**64**: 1401-10.<https://dx.doi.org/10.1007/s00234-022-02964-3>

246. Chen J, Wang Z, Tu Y, Liu X, Jorgenson K, Ye G, et al. Regional Homogeneity and Multivariate Pattern Analysis of Cervical Spondylosis Neck Pain and the Modulation Effect of Treatment. *Frontiers in neuroscience* 2018;**12**: 900.<https://dx.doi.org/10.3389/fnins.2018.00900>

247. Pandey S, Jain N, Singh A, Paliwal VK, Kumar S. MRI Evaluation of Microstructural and Perfusion Changes in Patients with Hemsensory Neurological Syndromes. *Neurology India* 2024;**72**: 553-60.<https://dx.doi.org/10.4103/neuroindia.NI_1050_20>

248. Park JG, Hong BY, Park H-Y, Yoo YJ, Yoon M-J, Kim J-S, et al. Alteration of White Matter in Patients with Central Post-Stroke Pain. *Journal of personalized medicine* 2021;**11**.<https://dx.doi.org/10.3390/jpm11050417>

249. Scheliga S, Dohrn MF, Habel U, Lampert A, Rolke R, Lischka A, et al. Reduced Gray Matter Volume and Cortical Thickness in Patients With Small-Fiber Neuropathy. *The Journal of Pain* 2024;**25**.10.1016/j.jpain.2024.01.001

250. van Gool R, Far A, Drenthen GS, Jansen JFA, Goijen CP, Backes WH, et al. Peripheral Pain Captured Centrally: Altered Brain Morphology on MRI in Small Fiber Neuropathy Patients With and Without an SCN9A Gene Variant. *The journal of pain* 2024;**25**: 730-41.<https://dx.doi.org/10.1016/j.jpain.2023.10.002>

251. Mole TB, MacIver K, Sluming V, Ridgway GR, Nurmikko TJ. Specific brain morphometric changes in spinal cord injury with and without neuropathic pain. *NeuroImage Clinical* 2014;**5**: 28-35.<https://dx.doi.org/10.1016/j.nicl.2014.05.014>

252. Yoon EJ, Kim YK, Shin HI, Lee Y, Kim SE. Cortical and white matter alterations in patients with neuropathic pain after spinal cord injury. *Brain research* 2013;**1540**: 64-73.<https://dx.doi.org/10.1016/j.brainres.2013.10.007>

253. Gustin SM, Wrigley PJ, Siddall PJ, Henderson LA. Brain anatomy changes associated with persistent neuropathic pain following spinal cord injury. *Cerebral cortex (New York, NY : 1991)* 2010;**20**: 1409-19.<https://dx.doi.org/10.1093/cercor/bhp205>

254. Liu R, Qiao N, Shi S, Li S, Wang Y, Song J, et al. Deficits in ascending pain modulation pathways in breast cancer survivors with chronic neuropathic pain: A resting-state fMRI study. *Frontiers in neurology* 2022;**13**: 959122.<https://dx.doi.org/10.3389/fneur.2022.959122>

255. Chao C-C, Hsieh P-C, Janice Lin C-H, Huang S-L, Hsieh S-T, Chiang M-C. Limbic Connectivity Underlies Pain Treatment Response in Small-Fiber Neuropathy. *Annals of neurology* 2023;**93**: 655-67.<https://dx.doi.org/10.1002/ana.26577>

256. Park E, Park JW, Kim E, Min Y-S, Lee HJ, Jung T-D, et al. Effects of Alterations in Resting-State Neural Networks on the Severity of Neuropathic Pain after Spinal Cord Injury. *Bioengineering (Basel, Switzerland)* 2023;**10**.<https://dx.doi.org/10.3390/bioengineering10070860>

257. Teixeira M, Mancini C, Wicht CA, Maestretti G, Kuntzer T, Cazzoli D, et al. Beta Electroencephalographic Oscillation Is a Potential GABAergic Biomarker of Chronic Peripheral Neuropathic Pain. *Frontiers in neuroscience* 2021;**15**: 594536.<https://dx.doi.org/10.3389/fnins.2021.594536>

258. Stern J, Jeanmonod D, Sarnthein J. Persistent EEG overactivation in the cortical pain matrix of neurogenic pain patients. *NeuroImage* 2006;**31**: 721-31.<https://dx.doi.org/10.1016/j.neuroimage.2005.12.042>

259. Rajan J, Gaur GS, Shanmugavel K, S A. Relation between heart rate variability and spectral analysis of electroencephalogram in chronic neuropathic pain patients. *The Korean journal of physiology & pharmacology : official journal of the Korean Physiological Society and the Korean Society of Pharmacology* 2024;**28**: 253-64.<https://dx.doi.org/10.4196/kjpp.2024.28.3.253>

260. Di Pietro F, Macey PM, Rae CD, Alshelh Z, Macefield VG, Vickers ER, et al. The relationship between thalamic GABA content and resting cortical rhythm in neuropathic pain. *Human brain mapping* 2018;**39**: 1945-56.<https://dx.doi.org/10.1002/hbm.23973>

261. Liu J, Liu H, Mu J, Xu Q, Chen T, Dun W, et al. Altered white matter microarchitecture in the cingulum bundle in women with primary dysmenorrhea: A tract-based analysis study. *Human brain mapping* 2017;**38**: 4430-43.<https://dx.doi.org/10.1002/hbm.23670>

262. Farmer MA, Huang L, Martucci K, Yang CC, Maravilla KR, Harris RE, et al. Brain White Matter Abnormalities in Female Interstitial Cystitis/Bladder Pain Syndrome: A MAPP Network Neuroimaging Study. *The Journal of urology* 2015;**194**: 118-26.<https://dx.doi.org/10.1016/j.juro.2015.02.082>

263. Lan X, Zhu X-Y, Bai W-X, Liu H-P, Wang H, Dun W-H, et al. White matter changes in young and middle-aged males with chronic prostatitis/chronic pelvic pain syndrome: Tract-based spatial statistics analysis. *The European journal of neuroscience* 2023;**58**: 3892-902.<https://dx.doi.org/10.1111/ejn.16154>

264. Dun W, Yang J, Yang L, Ma S, Guo C, Zhang X, et al. Abnormal white matter integrity during pain-free periovulation is associated with pain intensity in primary dysmenorrhea. *Brain imaging and behavior* 2017;**11**: 1061-70.<https://dx.doi.org/10.1007/s11682-016-9582-x>

265. As-Sanie S, Harris RE, Napadow V, Kim J, Neshewat G, Kairys A, et al. Changes in regional gray matter volume in women with chronic pelvic pain: a voxel-based morphometry study. *Pain* 2012;**153**: 1006-14.<https://dx.doi.org/10.1016/j.pain.2012.01.032>

266. Mordasini L, Weisstanner C, Rummel C, Thalmann GN, Verma RK, Wiest R, et al. Chronic pelvic pain syndrome in men is associated with reduction of relative gray matter volume in the anterior cingulate cortex compared to healthy controls. *The Journal of urology* 2012;**188**: 2233-7.<https://dx.doi.org/10.1016/j.juro.2012.08.043>

267. Bagarinao E, Johnson KA, Martucci KT, Ichesco E, Farmer MA, Labus J, et al. Preliminary structural MRI based brain classification of chronic pelvic pain: A MAPP network study. *Pain* 2014;**155**: 2502-9.<https://dx.doi.org/10.1016/j.pain.2014.09.002>

268. Kairys AE, Schmidt-Wilcke T, Puiu T, Ichesco E, Labus JS, Martucci K, et al. Increased brain gray matter in the primary somatosensory cortex is associated with increased pain and mood disturbance in patients with interstitial cystitis/painful bladder syndrome. *The Journal of urology* 2015;**193**: 131-7.<https://dx.doi.org/10.1016/j.juro.2014.08.042>

269. Maulitz L, Nehls S, Stickeler E, Ignatov A, Kupec T, Henn AT, et al. Psychological characteristics and structural brain changes in women with endometriosis and endometriosis-independent chronic pelvic pain. *Human reproduction (Oxford, England)* 2024;**39**: 2473-84.<https://dx.doi.org/10.1093/humrep/deae207>

270. Schweinhardt P, Kuchinad A, Pukall CF, Bushnell MC. Increased gray matter density in young women with chronic vulvar pain. *Pain* 2008;**140**: 411-9.<https://dx.doi.org/10.1016/j.pain.2008.09.014>

271. Farmer MA, Chanda ML, Parks EL, Baliki MN, Apkarian AV, Schaeffer AJ. Brain functional and anatomical changes in chronic prostatitis/chronic pelvic pain syndrome. *The Journal of urology* 2011;**186**: 117-24.<https://dx.doi.org/10.1016/j.juro.2011.03.027>

272. Kilpatrick LA, Kutch JJ, Tillisch K, Naliboff BD, Labus JS, Jiang Z, et al. Alterations in resting state oscillations and connectivity in sensory and motor networks in women with interstitial cystitis/painful bladder syndrome. *The Journal of urology* 2014;**192**: 947-55.<https://dx.doi.org/10.1016/j.juro.2014.03.093>

273. Yu Z, Yang H, Liu L-Y, Chen L, Su M-H, Yang L, et al. Altered cognitive control network mediates the association between long-term pain and anxiety symptoms in primary dysmenorrhea. *Neuroreport* 2024;**35**: 9-16.<https://dx.doi.org/10.1097/WNR.0000000000001971>

274. Lee L-C, Chen Y-H, Lin C-S, Li W-C, Low I, Tu C-H, et al. Unaltered intrinsic functional brain architecture in young women with primary dysmenorrhea. *Scientific reports* 2018;**8**: 12971.<https://dx.doi.org/10.1038/s41598-018-30827-6>

275. As-Sanie S, Kim J, Schmidt-Wilcke T, Sundgren PC, Clauw DJ, Napadow V, et al. Functional Connectivity is Associated With Altered Brain Chemistry in Women With Endometriosis-Associated Chronic Pelvic Pain. *The journal of pain* 2016;**17**: 1-13.<https://dx.doi.org/10.1016/j.jpain.2015.09.008>

276. Ge S, Hu Q, Guo Y, Xu K, Xia G, Sun C. Potential Alterations of Functional Connectivity Analysis in the Patients with Chronic Prostatitis/Chronic Pelvic Pain Syndrome. *Neural plasticity* 2021;**2021**: 6690414.<https://dx.doi.org/10.1155/2021/6690414>

277. Kutch JJ, Yani MS, Asavasopon S, Kirages DJ, Rana M, Cosand L, et al. Altered resting state neuromotor connectivity in men with chronic prostatitis/chronic pelvic pain syndrome: A MAPP: Research Network Neuroimaging Study. *NeuroImage Clinical* 2015;**8**: 493-502.<https://dx.doi.org/10.1016/j.nicl.2015.05.013>

278. Lan X, Niu X, Bai W-X, Li H-N, Zhu X-Y, Ma W-J, et al. The functional connectivity of the basal ganglia subregions changed in mid-aged and young males with chronic prostatitis/chronic pelvic pain syndrome. *Frontiers in human neuroscience* 2022;**16**: 1013425.<https://dx.doi.org/10.3389/fnhum.2022.1013425>

279. Lin Y, Bai Y, Liu P, Yang X, Qin W, Gu J, et al. Alterations in regional homogeneity of resting-state cerebral activity in patients with chronic prostatitis/chronic pelvic pain syndrome. *PloS one* 2017;**12**: e0184896.<https://dx.doi.org/10.1371/journal.pone.0184896>

280. Martucci KT, Shirer WR, Bagarinao E, Johnson KA, Farmer MA, Labus JS, et al. The posterior medial cortex in urologic chronic pelvic pain syndrome: detachment from default mode network-a resting-state study from the MAPP Research Network. *Pain* 2015;**156**: 1755-64.<https://dx.doi.org/10.1097/j.pain.0000000000000238>

281. Wei S-Y, Chao H-T, Tu C-H, Li W-C, Low I, Chuang C-Y, et al. Changes in functional connectivity of pain modulatory systems in women with primary dysmenorrhea. *Pain* 2016;**157**: 92-102.<https://dx.doi.org/10.1097/j.pain.0000000000000340>

282. Wu T-H, Tu C-H, Chao H-T, Li W-C, Low I, Chuang C-Y, et al. Dynamic Changes of Functional Pain Connectome in Women with Primary Dysmenorrhea. *Scientific reports* 2016;**6**: 24543.<https://dx.doi.org/10.1038/srep24543>

283. Gupta A, Bhatt RR, Naliboff BD, Kutch JJ, Labus JS, Vora PP, et al. Impact of early adverse life events and sex on functional brain networks in patients with urological chronic pelvic pain syndrome (UCPPS): A MAPP Research Network study. *PloS one* 2019;**14**: e0217610.<https://dx.doi.org/10.1371/journal.pone.0217610>

284. Han F, Liu H, Wang K, Yang J, Yang L, Liu J, et al. Correlation Between Thalamus-Related Functional Connectivity and Serum BDNF Levels During the Periovulatory Phase of Primary Dysmenorrhea. *Frontiers in human neuroscience* 2019;**13**: 333.<https://dx.doi.org/10.3389/fnhum.2019.00333>

285. Ding S, Zhu T, Tian Y, Xu P, Chen Z, Huang X, et al. Role of Brain-Derived Neurotrophic Factor in Endometriosis Pain. *Reproductive sciences (Thousand Oaks, Calif)* 2018;**25**: 1045-57.<https://dx.doi.org/10.1177/1933719117732161>

286. Liu J, Gu L, Huang Q, Hong S, Zeng X, Zhang D, et al. Altered gray matter volume in patients with herpes zoster and postherpetic neuralgia. *Journal of pain research* 2019;**12**: 605-16.<https://dx.doi.org/10.2147/JPR.S183561>

287. Liu X, Gu L, Liu J, Hong S, Luo Q, Wu Y, et al. MRI Study of Cerebral Cortical Thickness in Patients with Herpes Zoster and Postherpetic Neuralgia. *Journal of pain research* 2022;**15**: 623-32.<https://dx.doi.org/10.2147/JPR.S352105>

288. Li H, Li X, Feng Y, Gao F, Kong Y, Hu L. Deficits in ascending and descending pain modulation pathways in patients with postherpetic neuralgia. *NeuroImage* 2020;**221**: 117186.<https://dx.doi.org/10.1016/j.neuroimage.2020.117186>

289. Niu L, Hu Y, Yuan C-D, Wu X-Y, Zheng L-L, Zhang Y. Cerebral structural alterations in the patients undergoing postherpetic neuralgia: A VBM-MRI study. *Ibrain* 2022;**8**: 119-26.<https://dx.doi.org/10.1002/ibra.12027>

290. Cao S, Li Y, Deng W, Qin B, Zhang Y, Xie P, et al. Local Brain Activity Differences Between Herpes Zoster and Postherpetic Neuralgia Patients: A Resting-State Functional MRI Study. *Pain physician* 2017;**20**: E687-E99

291. Cao S, Song G, Zhang Y, Xie P, Tu Y, Li Y, et al. Abnormal Local Brain Activity Beyond the Pain Matrix in Postherpetic Neuralgia Patients: A Resting-State Functional MRI Study. *Pain physician* 2017;**20**: E303-E14

292. Wu Y, Wang C, Yu L, Qian W, Xing X, Zhang M, et al. Abnormal within- and cross-networks functional connectivity in different outcomes of herpes zoster patients. *Brain imaging and behavior* 2022;**16**: 366-78.<https://dx.doi.org/10.1007/s11682-021-00510-y>

293. Dai H, Jiang C, Wu G, Huang R, Jin X, Zhang Z, et al. A combined DTI and resting state functional MRI study in patients with postherpetic neuralgia. *Japanese journal of radiology* 2020;**38**: 440-50.<https://dx.doi.org/10.1007/s11604-020-00926-4>

294. Magon S, Sprenger T, Otti A, Papadopoulou A, Gundel H, Noll-Hussong M. Cortical Thickness Alterations in Chronic Pain Disorder: An Exploratory MRI Study. *Psychosomatic medicine* 2018;**80**: 592-8.<https://dx.doi.org/10.1097/PSY.0000000000000605>

295. Yoshino A, Okamoto Y, Kunisato Y, Yoshimura S, Jinnin R, Hayashi Y, et al. Distinctive spontaneous regional neural activity in patients with somatoform pain disorder: a preliminary resting-state fMRI study. *Psychiatry research* 2014;**221**: 246-8.<https://dx.doi.org/10.1016/j.pscychresns.2013.12.006>

296. Huang T, Zhao Z, Yan C, Lu J, Li X, Tang C, et al. Altered Spontaneous Activity in Patients with Persistent Somatoform Pain Disorder Revealed by Regional Homogeneity. *PloS one* 2016;**11**: e0151360.<https://dx.doi.org/10.1371/journal.pone.0151360>

297. Sun X, Pan X, Ni K, Ji C, Wu J, Yan C, et al. Aberrant Thalamic-Centered Functional Connectivity in Patients with Persistent Somatoform Pain Disorder. *Neuropsychiatric disease and treatment* 2020;**16**: 273-81.<https://dx.doi.org/10.2147/NDT.S231555>

298. Otti A, Guendel H, Wohlschlager A, Zimmer C, Noll-Hussong M. Frequency shifts in the anterior default mode network and the salience network in chronic pain disorder. *BMC psychiatry* 2013;**13**: 84.<https://dx.doi.org/10.1186/1471-244X-13-84>

299. Otti A, Guendel H, Henningsen P, Zimmer C, Wohlschlaeger AM, Noll-Hussong M. Functional network connectivity of pain-related resting state networks in somatoform pain disorder: an exploratory fMRI study. *Journal of psychiatry & neuroscience : JPN* 2013;**38**: 57-65.<https://dx.doi.org/10.1503/jpn.110187>

300. Liu Q, Zeng X-C, Jiang X-M, Zhou Z-H, Hu X-F. Altered Brain Functional Hubs and Connectivity Underlie Persistent Somatoform Pain Disorder. *Frontiers in neuroscience* 2019;**13**: 415.<https://dx.doi.org/10.3389/fnins.2019.00415>

301. Yoshino A, Okamoto Y, Doi M, Otsuru N, Okada G, Takamura M, et al. Regional brain functions in the resting state indicative of potential differences between depression and chronic pain. *Scientific reports* 2017;**7**: 3003.<https://dx.doi.org/10.1038/s41598-017-03522-1>

302. Ye Q, Yan D, Yao M, Lou W, Peng W. Hyperexcitability of Cortical Oscillations in Patients with Somatoform Pain Disorder: A Resting-State EEG Study. *Neural plasticity* 2019;**2019**: 2687150.<https://dx.doi.org/10.1155/2019/2687150>

303. Soros P, Bantel C. Chronic noncancer pain is not associated with accelerated brain aging as assessed by structural magnetic resonance imaging in patients treated in specialized outpatient clinics. *Pain* 2020;**161**: 641-50.<https://dx.doi.org/10.1097/j.pain.0000000000001756>

304. Kim JH, Ahn SH, Cho YW, Kim SH, Jang SH. The Relation Between Injury of the Spinothalamocortical Tract and Central Pain in Chronic Patients With Mild Traumatic Brain Injury. *The Journal of head trauma rehabilitation* 2015;**30**: E40-6.<https://dx.doi.org/10.1097/HTR.0000000000000121>

305. Seo CH, Park C-H, Jung MH, Baek S, Song J, Cha E, et al. Increased white matter diffusivity associated with phantom limb pain. *The Korean journal of pain* 2019;**32**: 271-9.<https://dx.doi.org/10.3344/kjp.2019.32.4.271>

306. Zhao W, Zhao L, Chang X, Lu X, Tu Y. Elevated dementia risk, cognitive decline, and hippocampal atrophy in multisite chronic pain. *Proceedings of the National Academy of Sciences of the United States of America* 2023;**120**: e2215192120.<https://dx.doi.org/10.1073/pnas.2215192120>

307. Riederer F, Landmann G, Gantenbein AR, Stockinger L, Egloff N, Sprott H, et al. Nondermatomal somatosensory deficits in chronic pain are associated with cerebral grey matter changes. *The world journal of biological psychiatry : the official journal of the World Federation of Societies of Biological Psychiatry* 2017;**18**: 227-38.<https://dx.doi.org/10.3109/15622975.2015.1073356>

308. Valet M, Gundel H, Sprenger T, Sorg C, Muhlau M, Zimmer C, et al. Patients with pain disorder show gray-matter loss in pain-processing structures: a voxel-based morphometric study. *Psychosomatic medicine* 2009;**71**: 49-56.<https://dx.doi.org/10.1097/PSY.0b013e31818d1e02>

309. Polli A, Weis L, Biundo R, Thacker M, Turolla A, Koutsikos K, et al. Anatomical and functional correlates of persistent pain in Parkinson's disease. *Movement disorders : official journal of the Movement Disorder Society* 2016;**31**: 1854-64.<https://dx.doi.org/10.1002/mds.26826>

310. Shen Y, Wang J, Peng J, Wu X, Chen X, Liu J, et al. Abnormal connectivity model of raphe nuclei with sensory-associated cortex in Parkinson's disease with chronic pain. *Neurological sciences : official journal of the Italian Neurological Society and of the Italian Society of Clinical Neurophysiology* 2022;**43**: 3175-85.<https://dx.doi.org/10.1007/s10072-022-05864-9>

311. Zhou X, Tan Y, Chen J, Wang C, Tang Y, Liu J, et al. Altered Functional Connectivity in Pain-Related Brain Regions and Its Correlation with Pain Duration in Bone Metastasis with Cancer Pain. *Disease markers* 2022;**2022**: 3044186.<https://dx.doi.org/10.1155/2022/3044186>

312. Wei X, Lai Y, Lan X, Tan Y, Zhang J, Liu J, et al. Uncovering brain functional connectivity disruption patterns of lung cancer-related pain. *Brain imaging and behavior* 2024;**18**: 576-87.<https://dx.doi.org/10.1007/s11682-023-00836-9>

313. Flowers M, Leung A, Schiehser DM, Metzger-Smith V, Delano-Wood L, Sorg S, et al. Severities in persistent mild traumatic brain injury related headache is associated with changes in supraspinal pain modulatory functions. *Molecular pain* 2021;**17**: 17448069211037881.<https://dx.doi.org/10.1177/17448069211037881>

314. Karafin MS, Chen G, Wandersee NJ, Brandow AM, Hurley RW, Simpson P, et al. Chronic pain in adults with sickle cell disease is associated with alterations in functional connectivity of the brain. *PloS one* 2019;**14**: e0216994.<https://dx.doi.org/10.1371/journal.pone.0216994>

315. You L, Yang B, Lu X, Yang A, Zhang Y, Bi X, et al. Similarities and differences between Chronic Primary Pain and Depression in brain activities: Evidence from Resting-State Microstates and auditory Oddball Task. *Behavioural brain research* 2024: 115319.<https://dx.doi.org/10.1016/j.bbr.2024.115319>

316. Jensen MP, Sherlin LH, Gertz KJ, Braden AL, Kupper AE, Gianas A, et al. Brain EEG activity correlates of chronic pain in persons with spinal cord injury: clinical implications. *Spinal cord* 2013;**51**: 55-8.<https://dx.doi.org/10.1038/sc.2012.84>

317. Lopes TS, Santana JE, Silva WS, Fraga FJ, Montoya P, Sa KN, et al. Increased Delta and Theta Power Density in Sickle Cell Disease Individuals with Chronic Pain Secondary to Hip Osteonecrosis: A Resting-State Eeg Study. *Brain topography* 2024;**37**: 859-73.<https://dx.doi.org/10.1007/s10548-023-01027-x>
