# Supplementary material for: A systematic review of brain health in adults with chronic pain
Source: Anaesthesia. 2025 Oct 14;81(2):248–62. doi: 10.1111/anae.70021 (PMC12803547; doi:10.1111/anae.70021)
Supplement: Supplementary file 4 — Appendix S1. Search strategy. Appendix S2. Data extraction form. [file ANAE-81-248-s001.docx]

**Appendix S1.** Search strategy.

The search strategy was developed with an information specialist (NR).

Medline (OvidSP)[1946-present]

1 ((persist* or intract* or chronic or longstanding or long standing or longterm or longterm or refractory or prolong* or long last* or sustain* or linger* or syndrome* or unremitting) adj3 pain*).ti,ab,kf.

2 Musculoskeletal Pain/ or Pain Insensitivity, Congenital/ or Pain, Postoperative/ or Eye Pain/ or Pelvic Girdle Pain/ or Back Pain/ or Pain, Intractable/ or Abdominal Pain/ or Neck Pain/ or Pain, Referred/ or Visceral Pain/ or Pelvic Pain/ or Patellofemoral Pain Syndrome/ or Complex Regional Pain Syndromes/ or Shoulder Pain/ or Cancer Pain/ or Myofascial Pain Syndromes/ or Pain Perception/ or Pain/ or Nociceptive Pain/ or Facial Pain/ or Chest Pain/ or Pain Threshold/ or Low Back Pain/ or Flank Pain/ or Chronic Pain/

3 brain health.mp.

4 (brain or white matter).mp.

5 (atroph* or alzheimer* or cogniti* or dement* or executive function or hippocamp* or memory or mental disorder or mental health or neurocogniti* or neurodegenerat* or stroke).mp.

6 ("Structural MRI" or "grey matter volume" or "grey matter volume" or "total brain volume" or "whole brain grey matter volume" or "hippocampus volume" or "white matter volume" or "white matter hyperintensities" or "WMH" or "functional MRI" or "magnetic resonance imaging" or "brain scan" or "brain imaging" or "cerebral blood flow" or "BOLD" or "resting state functional connectivity" or "task based functional connectivity" or "fractional anisotropy" or "mean diffusivity" or "axial diffusivity" or "radial diffusivity" or "Brain age" or "brain age gap" or "Brain atrophy and lesion index" or "BALI" or "PET" or "Positron emission tomography" or "amyloid" or "tau" or "transcranial doppler ultrasound" or "transcranial magnetic stimulation" or "functional near infrared spectroscopy" or "fNIRS" or "trail making test" or "TMT" or "mini mental state examination" or "MMSE" or "Stroop test" or "RAVLT" or "Rey Auditory verbal learning test" or "Montreal cognitive assessment" or "MoCA" or "Digit span" or "Digit symbol substitution test" or "DSST" or "Verbal fluency" or "Wechsler adult intelligence scale" or "California verbal learning test" or "CVLT" or "ApoE4" or "BDNF" or "Brain derived neurotrophic factor" or "Cerebrospinal fluid" or "neurofilament light" or "tau" or "EEG" or "Electroencephalography" or "LIBRA Lifestyle for brain health index" or "Mindreader" or "Barratt impulsiveness scale" or "Geriatric depression scale" or "PHQ-9" or "Patient health questionnaire" or "Hospital anxiety and depression scale" or "HADS" or "Beck depression inventory").mp. [mp=title, book title, abstract, original title, name of substance word, subject heading word, floating sub-heading word, keyword heading word, organism supplementary concept word, protocol supplementary concept word, rare disease supplementary concept word, unique identifier, synonyms, population supplementary concept word, anatomy supplementary concept word]

7 1 or 2

8 4 and 6

9 4 and 5

10 3 or 8 or 9

11 7 and 10

12 limit 11 to english language

13 (baboon$1 or bovine$1 or canine$1 or cat$1 or chimpanzee$1 or cow$1 or dog$1 or feline$1 or goat$1 or hens or macque$1 or mice or monkey$1 or (mouse adj2 model$1) or murine$1 or ovine or pig$1 or porcine or (non-human adj2 primate$1) or sheep or rabbit$1 or rat or rats or rattus or rhesus or rodent$1 or zebrafish).ti.

14 12 not 13

15 (review or systematic review).pt.

16 14 not 15

17 14 and 15

**Appendix S2.** Data extraction form.

General information

**Author last name and year (e.g. Yang 2021)**

**Title**

**Country in which the study was conducted**

1. United States
2. UK
3. France
4. Canada
5. Australia
6. New Zealand
7. China
8. Japan
9. Other

Characteristics of included studies

Methods

**Study design**

1. Randomised controlled trial
2. Non-randomised experimental study
3. Cohort study
4. Cross sectional study
5. Case control study
6. Other

Participants

**Chronic pain group studied (tick all that apply)**

1. Fibromyalgia
2. Chronic back pain
3. Abdominal pain
4. Burning mouth syndrome
5. Cervical spondylosis
6. Chest pain
7. Cluster headache
8. Complex regional pain syndrome (CRPS)
9. Diabetic neuropathy
10. Facial pain
11. Foot pain
12. Hand pain
13. Migraine
14. Hip pain
15. Irritable bowel syndrome (IBS)
16. Knee pain
17. Musculoskeletal pain
18. Neck pain
19. Neuropathic pain
20. Osteoarthritis
21. Pelvic pain
22. Phantom limb pain
23. Postherpetic neuralgia
24. Rheumatoid arthritis
25. Shoulder pain
26. Sickle cell disease
27. Trigeminal neuralgia (TN)
28. Vulvodynia
29. Other (mixed)

**Further detail about pain type**

E.g. Shoulder pain in osteoarthritis, or what the pain type is if you selected 'Other (mixed)'. If not applicable then leave blank.

**Number of participants in pain group (e.g. 20)**

**Number of healthy controls (e.g. 20)**

**Age group of participants (tick all that apply)**

1. 18-30y
2. 31-50y
3. 51-70y
4. >71y

Outcomes

Outcome measures (tick all that apply)

**Imaging**

1. Grey matter volume (including cortical thickness)
2. White matter hyperintensities
3. Total brain volume
4. Cerebral blood flow in specific regions
5. Resting state functional connectivity
6. Fractional anisotropy
7. Mean diffusivity
8. Brain age gap calculations
9. Brain atrophy and lesion index (BALI)
10. PET amyloid load or presence
11. PET tau
12. Transcranial doppler ultrasound
13. Transcranial magnetic stimulation (TMS)
14. Functional near infrared spectroscopy (fNIRS)

**Cognitive testing**

1. Trail making test (TMT) A or/and B
2. Mini mental status examination (MMSE)
3. Stroop test
4. Rey Auditory Verbal Learning Test (RAVLT)
5. Montreal cognitive assessment (MoCA)
6. Digit span
7. Digit symbol substitution test (DSST)
8. Verbal fluency
9. Wechsler adult intelligence scale
10. Hopkins verbal learning test (HVLT)

**Clinical**

1. EEG
2. Lifestyle for brain health (LIBRA) index
3. Mindreader (EEG software)
4. Diagnosis of dementia

**Biological**

1. Blood ApoE4 genotype
2. Blood brain derived neurotrophic factor (BDNF)
3. Blood ABeta 42 or 40 levels
4. CSF neurofilament light
5. CSF tau levels

**Mental health**

1. Barratt impulsiveness scale
2. Geriatric depression scale
3. Patient health questionnaire (PHQ)-9 depression scale
4. Hospital anxiety and depression (HADS) scales
5. Beck depression inventory (BDI)

**Main finding**

e.g. Pain group had lower hippocampal volumes than controls by 6% (p<0.0002)

**For imaging studies, brain region(s) implicated**

e.g. Hippocampus, substantia nigra, caudate nucleus
